# National and subnational burden of brain and central nervous system cancers in Iran, 1990–2019: Results from the global burden of disease study 2019

**DOI:** 10.1002/cam4.5553

**Published:** 2023-01-09

**Authors:** Mahdi Mahdavi, Sahar Saeedi Moghaddam, Mohsen Abbasi‐Kangevari, Esmaeil Mohammadi, Parnian Shobeiri, Guive Sharifi, Ali Jafari, Negar Rezaei, Narges Ebrahimi, Nazila Rezaei, Seyyed‐Hadi Ghamari, Mohammad‐Reza Malekpour, Majid Khalili, Bagher Larijani, Farzad Kompani

**Affiliations:** ^1^ Non‐Communicable Diseases Research Center, Endocrinology and Metabolism Population Sciences Institute Tehran University of Medical Sciences Tehran Iran; ^2^ Institute of Medical Science and Technology (IMSAT) Shahid Beheshti University Tehran Iran; ^3^ Kiel Institute for the World Economy Kiel Germany; ^4^ Department of Neurological Surgery University of Oklahoma Health Sciences Center Oklahoma Oklahoma USA; ^5^ Department of Neurosurgery, Loghman Hospital Shahid Beheshti University of Medical Sciences Tehran Iran; ^6^ Endocrinology and Metabolism Research Center, Endocrinology and Metabolism Clinical Sciences Institute Tehran University of Medical Sciences Tehran Iran; ^7^ Division of Hematology and Oncology, Children's Medical Center, Pediatrics Center of Excellence Tehran University of Medical Sciences Tehran Iran

**Keywords:** brain and central nervous system cancers, epidemiologic study, global burden of disease, Iran

## Abstract

**Introduction:**

Central nervous system cancers (CNS cancers) impose a significant burden upon healthcare systems worldwide. Currently, the lack of a comprehensive study to assess various epidemiological indexes of CNS cancers on national and subnational scales in Iran can hamper healthcare planning and resource allocation in this regard. This study aims to fill this gap by providing estimates of CNS cancer epidemiological measures on national and subnational levels in Iran from 1990 to 2019.

**Materials and Methods:**

This study is a part of Global Burden of Disease (GBD) 2019 that contains epidemiological measures including prevalence, incidence, mortality, Disability‐Adjusted Life Years (DALYs), Years Lived with Disability (YLDs), and Years of Life Lost (YLLs) of CNS cancers. Age standardization was utilized for comparing different provinces.

**Results:**

In 2019, 5811 (95% Uncertainty Interval: 2942–7046) national new cases and 3494 (1751–4173) deaths due to CNS cancers were reported. National age‐standardized incidence (ASIR), deaths (ASDR), and DALYs rates were 7.3 (3.7–8.8), 4.6 (2.3–5.5), and 156.4 (82.0–187.0) per 100,000 in 2019, respectively. Subnational results revealed that ASDR and ASIR have increased in the past 30 years in all provinces. Although incidence rates have increased in all age groups and genders since 1990, death rates have remained the same for most age groups and genders except for young patients aged under 15, where a decrease in mortality and YLLs can be observed.

**Conclusion:**

The incidence, deaths, and DALYs of CNS cancers increased at national and subnational levels. These findings should be considered for planning and resource allocation.

## 1 INTRODUCTION

Primary brain and spinal cord tumors, also known as central nervous system (CNS) cancers, are estimated to affect 7 to 11 individuals per 100,000 person‐years worldwide.^1^, ^2^ In a recent report by the Global Burden of Disease (GBD) brain and other CNS cancers collaboration, around 330,000 novel CNS cancer cases have emerged worldwide. With an estimate of 227,000 deaths and 7.7 million Disability‐Adjusted Life Years (DALYs), this fatal diagnosis imposes a significant burden on every age group.^1^, ^3^ The enhanced life expectancy of most age groups in the world,^4^ along with higher detection rates of CNS cancers, has inevitably caused an increase in the incidence of neoplasms in the geriatric population.^5^, ^6^, ^7^, ^8^ As a result, age‐standardized estimates of CNS cancer incidence rates have risen significantly from 1990 to 2017.^2^

Lack of efficient treatment along with significant invasion and late detection is the significant factor in patient morbidity and mortality. Early detection of CNS tumors is paramount to expected survival since less resection of brain tissue is required and, in most cases of early diagnosis, the tumor is resectable by surgery.^9^ However, except for some cases where relatives of patients are screened,^10^ the absence of a standard early detection method results in patients often being diagnosed when neurological signs have emerged, and the tumor has become large or has spread to other regions.^9^ Elongated diagnosis to expiration interval due to medical advances has caused exacerbation of the medical and psychological comorbidities,^6^ quality of life indices, and health expenditures^11^, ^12^ in patients with CNS cancers, making the disease a significant burden for healthcare systems. Previous studies have reported epidemiological characteristics of CNS cancers in Iran and the Middle East and North Africa (MENA) region. However, lack of subnational or national focus on Iran,^2^, ^13^, ^14^ absence of rigorous data sources, and being relatively outdated for the present time^15^, ^16^ prompt the need for up‐to‐date studies in this regard.

Studying the national and subnational burden characteristics of CNS cancers can provide valuable information for healthcare systems and health institutes to optimize resource allocation and draft population‐specific policies in fast senescing and transition community of Iran.^17^, ^18^ The GBD study aims to measure the health impact of all diseases from 1990 to the latest annual update. This study used data from GBD 2019 Study^19^ to evaluate Age‐Standardized Rates (ASR) of incidence, prevalence, deaths, DALYs, Years Lived with Disability (YLDs), and Years of Life Lost (YLLs) of CNS cancers in the Iranian population in 1990 and 2019, grouped by gender and 5‐year age groups. Furthermore, the changes in the aforementioned indices between 1990 and 2019 were also investigated to discover alteration trends.

## 2 MATERIALS AND METHODS

### 2.1 Data sources

The current study is executed as a part of GBD 2019, which evaluates epidemiological variables, including incidence, prevalence, deaths, YLDs, YLLs, and DALYs for 369 diseases and injuries, 286 mortality causes, and 87 risk factors in 204 countries and regions. GBD 2019 contains subnational data from 21 countries, including Iran.^20^, ^21^ Data on the brain and other central nervous system cancers (B.1.22) were obtained from an online query tool, the Global Health Data Exchange (GHDx), for 31 provinces of Iran. Detailed methods for the GBD 2019 study have been published previously.^22^ Twenty‐one sources were used to analyze CNS cancers metrics in Iran reporting deaths, incidence, and prevalence (Table S1).

### 2.2 Mortality estimates

Cause‐specific mortality was calculated from vital registration and cancer registry data using death to incidence ratios. To improve the comparability of mortality data sources, data were mapped to the GBD list of disease causes, and three methods were utilized; (a) reclassification and redistribution of codes that are non‐specific or unspecific; (b) Bayesian geospatial regression software (CODEm, Cause of Death Ensemble model); (c) CoDCorrect algorithm to adjust the single‐cause mortality estimates.

### 2.3 Incidence, prevalence, YLLs, YLDs, and DALYs estimates

Data from existing scientific reports on cohorts, registries, population surveys, micro‐data from the registry, cohort studies, and health system administrative systems were utilized to obtain estimates for the incidence and prevalence of CNS cancers. Survival (incidence) of CNS cancers was extracted from mortality to incidence ratios across various geographical regions and age categories. Afterward, the 10‐year prevalence was calculated for each incidence cohort and was divided into four intervals; diagnosis and treatment, remission, metastasis, and terminal. YLDs were obtained by multiplying the prevalence of each interval by its disability weight and by adding the procedure‐related morbidity associated with treatment. YLLs due to CNC cancers were calculated using normative global life expectancy and the number of deaths by age. Finally, DALYs were calculated by summing YLDs and YLLs.

### 2.4 Decomposition analysis

The contribution of different factors, including population growth, aging, and variations in age‐specific incidence rates to the witnessed CNS cancers new cases changes, was examined. In the first step, the age structure and age‐specific incidence rate of CNS cancers in 1990 were applied to the population size of 2019. In the second step, the aforementioned approaches were applied to the population size of 1990. The interpretation of the mentioned frameworks is as follows: (a) the difference between the incident cases in the first and *second* steps is considered as the contribution of age structure changes during the past 30 years; (b) the difference between the incident cases of the first step and the actual incident cases in 1990 is attributable to the population growth; (c) the differences between the incidence value of the second step and the actual incident values in 2019 is attributable to the age‐specific incidence rate changes.^23^

### 2.5 Data analysis

Age‐standardization was performed by applying a universal age structure from the year 2019. The ASRs of CNS cancers for all 31 provinces of Iran were calculated using the GBD world population standard and reported per 100,000 population. The 95% Uncertainty Interval (UI) was obtained for each metric from the 2.5th and 97.5th ranked percentiles of the uncertainty distribution by choosing 1000 random samples from the posterior distribution. Results are considered significant if the UI does not envelope zero. The differences in values of each metric from 1990 to 2019 were computed to calculate the total percent change.

Socio‐demographic Index (SDI), first introduced in GBD 2015, was applied to assess the ASRs of CNS cancers with each province's socioeconomic status based on an interpretable scale.^24^ The estimation of SDI was based on three factors: lag distributed income per capita, mean educational attainment (aged≥15), and total fertility rate (aged≤25). The SDI score ranges from zero to 1, with higher values associated with higher income per capita, educational attainment, and lower total fertility rate. Each province was categorized based on the SDI quintile. All data analysis and illustrations were performed using R statistical package v4.1 (http://www.r‐project.org, RRID: SCR_001905).

## 3 RESULTS

### 3.1 Incidence, prevalence, and deaths

In 2019, 5811 (95% UI: 2942–7046) new cases and 23,225 (12,066–29,504) prevalent cases of CNS cancers have occurred in Iran, showing a 2.3‐ and 3.2‐fold increase since 1990, respectively (Table 1). Moreover, the total number of CNS cancers death cases in Iran was 3494 (1751–4173), which has shown a 2.0‐fold increase since 1990. National Age‐Standardized Incidence Rates (ASIRs) and Age‐Standardized Prevalence Rates (ASPRs) of CNS cancers in 2019 were 7.3 (3.7–8.8) and 27.7 (14.4–35.1), respectively. Gender‐specific results showed an ASIR of 6.7 (3.4–8.6) for females and 7.8 (3.3–10.1) for males; regarding ASPR, the rates were 25.4 (12.9–34.9) for females and 30.0 (13.0–41.4) for males. The overall national Age‐standardized Death Rates (ASDRs) of CNS cancers were 4.6 (2.3–5.5), and reports by gender revealed an ASDR of 4.3(2.2–5.3) for females and 4.8 (2.0–6.2) for males (Table 1). National and subnational trends of ASPR, ASIR, and ASDR reveal an ascending trend in the recent 15 years after an initial steady trend from 1990. Moreover, the ASDR pattern seems to be reaching a plateau state as opposed to ASIR and ASPR (Figures [Link], [Link]).

**TABLE 1:** The age‐standardized incidence and deaths rates at national and subnational levels in 1990 and 2019, by sex

Location		1990						2019					
		ASIR (Per 100,000 population)			ASDR (Per 100,000 population)			ASIR (Per 100,000 population)			ASDR (Per 100,000 population)		
		T	F	M	T	F	M	T	F	M	T	F	M
Iran (Islamic Republic of)		5.6 (3.3–7.3)	4.7 (2.4–7.6)	6.4 (3.4–8.8)	4.6 (2.8–5.9)	3.9 (2.0–6.1)	5.3 (2.9–7.2)	7.3 (3.7–8.8)	6.7 (3.4–8.6)	7.8 (3.3–10.1)	4.6 (2.3–5.5)	4.3 (2.2–5.3)	4.8 (2–6.2)
Subnational	Alborz	5.7 (3.1–8.0)	4.8 (2.4–9.0)	6.4 (3.1–10.3)	4.6 (2.5–6.4)	4.0 (2.0–7.3)	5.1 (2.5–8.2)	8.5 (4.2–10.9)	7.6 (3.7–10.4)	9.3 (3.5–13.2)	4.7 (2.2–6.1)	4.3 (2.0–5.7)	5.0 (1.8–7.2)
	Ardebil	4.5 (3.2–6.4)	7.2 (3.3–15.7)	10.6 (5.7–18.4)	3.8 (2.7–5.3)	3.0 (1.8–5.5)	4.4 (2.8–6.7)	6.5 (3.8–8.1)	5.5 (3.3–7.3)	7.6 (3.6–10.3)	4.4 (2.5–5.5)	3.7 (2.2–4.7)	5.1 (2.4–6.9)
	Bushehr	5.4 (3.0–7.3)	4.7 (2.1–7.7)	6.1 (3.1–8.9)	4.6 (2.6–6.2)	3.9 (1.8–6.3)	5.3 (2.6–7.7)	7.1 (3.7–9.1)	6.7 (3.5–9.3)	7.5 (3.2–10.2)	4.6 (2.3–5.9)	4.3 (2.2–5.8)	4.9 (2.1–6.7)
	Chahar Mahaal and Bakhtiari	5.6 (3.2–7.7)	4.3 (1.9–7)	6.9 (3.3–10.5)	4.6 (2.6–6.3)	3.5 (1.6–5.7)	5.7 (2.7–8.8)	6.7 (3.5–8.8)	5.6 (2.9–8.3)	7.9 (3.4–11.3)	3.9 (2.0–5.2)	3.2 (1.7–4.7)	4.6 (1.9–6.7)
	East Azarbayejan	7.2 (3.4–10)	5.9 (2.2–9.9)	8.3 (3.2–12.8)	6.1 (3.0–8.5)	5.0 (2.0–8.1)	7.0 (2.9–11.0)	8.5 (3.9–11.3)	8.1 (3.4–11.7)	8.9 (3.6–13)	5.5 (2.5–7.3)	5.2 (2.3–7.2)	5.9 (2.2–8.6)
	Fars	5.7 (3.3–7.6)	4.8 (2.2–7.8)	6.5 (3.3–10)	4.6 (2.8–6.3)	3.9 (1.9–6.1)	5.4 (2.7–8.0)	8.8 (4.3–11.9)	8.1 (3.6–11.4)	9.6 (3.9–14.1)	5.2 (2.5–6.9)	4.8 (2.2–6.6)	5.6 (2.3–8.0)
	Gilan	3.8 (2.9–5.3)	3.1 (1.9–5.0)	4.6 (3.1–7.1)	3.1 (2.4–4.3)	2.6 (1.6–3.9)	3.8 (2.6–5.5)	5.9 (3.9–7.2)	5.2 (3.5–6.7)	6.6 (3.5–8.8)	3.7 (2.4–4.5)	3.3 (2.2–4.3)	4.0 (2.1–5.3)
	Golestan	4.4 (3.0–6.2)	3.6 (2.2–5.7)	5.2 (2.9–7.8)	3.8 (2.6–5.1)	3.1 (2.0–4.9)	4.5 (2.6–6.8)	5.9 (3.9–7.2)	5.1 (3.5–6.4)	6.7 (3.7–8.9)	4.1 (2.8–5.0)	3.6 (2.4–4.5)	4.6 (2.5–6.0)
	Hamadan	4.7 (3.2–6.6)	4.0 (2.1–6.7)	5.5 (3.2–8.9)	3.9 (2.8–5.3)	3.3 (1.9–5.4)	4.5 (2.8–6.7)	6.2 (3.9–7.7)	5.6 (3.4–7.4)	6.8 (3.7–9.2)	3.9 (2.5–4.9)	3.6 (2.2–4.6)	4.3 (2.3–5.8)
	Hormozgan	3.6 (2.3–5.7)	3.1 (1.7–5.5)	4.0 (2.3–7.1)	3.3 (2.1–4.8)	2.8 (1.6–4.8)	3.6 (2.2–5.9)	4.8 (3.5–5.9)	4.6 (3.1–6.1)	5.1 (3.3–6.8)	3.3 (2.3–4.1)	3.3 (2.1–4.3)	3.4 (2.1–4.6)
	Ilam	4.7 (2.9–6.2)	3.7 (1.8–6.2)	5.4 (2.9–7.8)	3.9 (2.5–5.2)	3.1 (1.6–5.0)	4.6 (2.5–6.7)	6.6 (3.9–8.3)	5.9 (3.5–7.9)	7.3 (3.7–9.9)	4.2 (2.4–5.2)	3.9 (2.2–5.0)	4.5 (2.1–6.0)
	Isfahan	5.7 (3.0–7.6)	4.9 (2.2–7.6)	6.4 (3.0–9.8)	4.5 (2.4–6.1)	3.8 (1.9–5.9)	5.1 (2.3–7.8)	8.4 (3.7–11.2)	8.0 (3.5–11.1)	8.9 (3.2–13)	5.0 (2.1–6.7)	4.9 (2.0–6.6)	5.2 (1.7–7.6)
	Kerman	6.0 (3.3–8.1)	5.0 (2.2–8.0)	6.9 (3.1–10.7)	5.2 (2.9–7.0)	4.3 (2.0–7.0)	6.0 (2.9–9.3)	7.0 (3.7–9.1)	6.7 (3.3–9.2)	7.3 (3.4–10.3)	4.8 (2.5–6.1)	4.6 (2.2–6.1)	5.0 (2.3–7.1)
	Kermanshah	7.0 (3.3–9.9)	5.5 (2.1–9.3)	8.4 (3.3–13.3)	6.1 (2.9–8.7)	4.7 (1.9–7.6)	7.2 (2.9–11.8)	8.0 (3.9–10.7)	7.1 (3.3–10)	8.9 (3.6–13.1)	5.2 (2.6–7.0)	4.7 (2.2–6.5)	5.8 (2.3–8.5)
	Khorasan–e–Razavi	5.9 (3.4–8.1)	4.7 (2.2–8.2)	7.0 (3.2–10.9)	5.0 (3–6.7)	4.0 (1.9–6.8)	6.0 (2.9–9.1)	7.3 (3.9–9.4)	6.6 (3.5–9.1)	8.0 (3.4–11.4)	4.9 (2.6–6.2)	4.3 (2.3–5.7)	5.4 (2.2–7.6)
	Khuzestan	4.2 (3.2–5.6)	3.4 (2.1–5.3)	4.9 (3.2–7.2)	3.6 (2.7–4.7)	2.9 (1.9–4.5)	4.2 (2.7–6.1)	6.6 (3.9–8.4)	6.0 (3.5–8.0)	7.2 (3.5–9.9)	4.3 (2.5–5.3)	3.9 (2.3–5.0)	4.7 (2.2–6.3)
	Kohgiluyeh and Boyer–Ahmad	4.6 (3.1–6.3)	3.5 (1.8–6.2)	5.5 (3–8.6)	3.8 (2.5–5.2)	2.9 (1.6–4.9)	4.5 (2.6–7.0)	7.4 (4.1–9.8)	6.3 (3.7–8.8)	8.5 (3.6–12.4)	4.2 (2.2–5.5)	3.5 (2.1–4.8)	4.8 (1.9–7.0)
	Kurdistan	6.0 (3.3–8.3)	5.0 (2.0–9.3)	6.8 (3.3–10.9)	5.0 (2.9–7.1)	4.1 (1.7–7.3)	5.8 (2.9–9.1)	6.6 (3.6–8.6)	6.1 (3.1–8.3)	7.1 (3.4–10.1)	4.4 (2.3–5.7)	4.2 (2.1–5.7)	4.7 (2.1–6.5)
	Lorestan	5.4 (3.1–7.6)	4.3 (2.1–7.6)	6.3 (3.1–9.9)	4.7 (2.7–6.5)	3.8 (1.8–6.5)	5.5 (2.7–8.5)	7.3 (3.7–9.8)	6.3 (3.0–9.0)	8.3 (3.7–12.0)	4.8 (2.4–6.5)	4.2 (1.9–5.9)	5.5 (2.3–8.1)
	Markazi	7.4 (3.5–10.4)	6.1 (2.3–10.2)	8.8 (3.4–13.9)	6.2 (3.0–8.8)	5.1 (2.0–8.7)	7.4 (3.0–11.8)	8.6 (3.5–11.8)	7.6 (3.2–10.7)	9.6 (3.1–14.6)	5.5 (2.2–7.3)	4.8 (2.0–6.6)	6.1 (1.9–9.0)
	Mazandaran	4.6 (3.3–5.9)	3.9 (2.3–6.1)	5.3 (3.2–7.3)	3.6 (2.5–4.6)	3.1 (1.9–4.8)	4.1 (2.5–5.8)	7.9 (4.0–10.2)	7.2 (3.8–9.8)	8.5 (3.6–12.0)	4.4 (2.2–5.7)	4.1 (2.1–5.5)	4.8 (1.9–6.7)
	North Khorasan	4.6 (3.0–6.9)	3.9 (2.0–7.3)	5.2 (2.9–8.6	4.0 (2.8–5.8)	3.5 (1.8–6.3)	4.6 (2.8–7.3)	6.2 (3.7–7.7)	6.1 (3.3–8.0)	6.3 (3.4–8.3)	4.3 (2.6–5.3)	4.3 (2.4–5.5)	4.4 (2.3–5.7)
	Qazvin	5.8 (3.0–8.0)	5.2 (2.1–9.5)	6.4 (3.0–9.8)	5.0 (2.6–7.0)	4.5 (1.8–7.9)	5.6 (2.6–8.4)	8.8 (3.5–11.8)	8 (3.0–11.8)	9.5 (3.2–13.9)	5.9 (2.2–8.1)	5.4 (1.8–7.6)	6.5 (2.1–9.6)
	Qom	6.9 (2.9–9.8)	6.4 (2.2–11.0)	7.3 (2.9–11.5)	6.1 (2.7–8.5)	5.7 (2.0–9.7)	6.4 (2.6–10.2)	8.6 (3.0–11.6)	8.5 (3.1–12.4)	8.7 (2.6–13.0)	5.7 (2.0–7.8)	5.9 (2.0–8.3)	5.6 (1.6–8.4)
	Semnan	5.3 (3.2–7.1)	4.4 (2.1–7.4)	6.3 (3.1–9.4)	4.6 (2.7–6.1)	3.8 (1.8–6)	5.4 (2.7–8.2)	6.7 (3.5–8.6)	6.2 (3.2–8.4)	7.3 (3.2–10.1)	4.3 (2.2–5.6)	4 (2–5.4)	4.7 (2.1–6.5)
	Sistan and Baluchistan	3.0 (1.5–5.8)	2.2 (1.1–4.3)	3.7 (1.7–7.9)	2.7 (1.5–5.2)	1.9 (1.0–4.0)	3.3 (1.7–6.9)	3.9 (3.1–5.1)	3.3 (2.4–4.9)	4.4 (3.2–6.3)	3.0 (2.4–4.0)	2.5 (1.9–3.9)	3.4 (2.4–4.8)
	South Khorasan	4.9 (3.1–6.9)	3.9 (2.1–7.1)	5.7 (3.1–8.9)	4.1 (2.8–5.7)	3.4 (1.8–5.9)	4.9 (2.8–7.6)	6.3 (3.4–8.0)	5.9 (3.0–8.0)	6.8 (3.0–9.3)	4.2 (2.3–5.3)	3.9 (2.1–5.1)	4.5 (1.9–6.1)
	Tehran	6.4 (2.9–9.4)	5.8 (3.0–11.2)	7.0 (2.4–11.7)	4.8 (2.2–7.1)	4.4 (2.2–8.1)	5.1 (1.7–8.7)	7.0 (2.7–9.0)	6.9 (2.8–9.1)	7.1 (1.9–10.1)	4.0 (1.5–5.2)	4.1 (1.7–5.4)	3.9 (1.0–5.5)
	West Azarbayejan	6.4 (3.2–8.6)	5.3 (2.2–8.7)	7.4 (3.2–11.3)	5.4 (2.9–7.4)	4.4 (2.0–7.2)	6.3 (2.9–9.6)	8.0 (3.6–10.6)	7.4 (3.3–10.5)	8.6 (3.3–12.4)	5.4 (2.5–7.1)	5.0 (2.3–6.7)	5.9 (2.2–8.3)
	Yazd	7.6 (3.1–10.8)	6.8 (2.3–11.4)	8.5 (3.0–13.3)	6.6 (2.7–9.3)	5.9 (2.1–9.7)	7.4 (2.6–11.8)	11.1 (3.9–15.5)	10.7 (3.5–15.8)	11.5 (3.4–18.1)	6.6 (2.2–9.2)	6.4 (2.1–9.1)	6.7 (1.9–10.6)
	Zanjan	4.8 (3.2–6.3)	3.9 (2.0–6.5)	5.7 (3.2–8.2)	4.1 (2.8–5.2)	3.3 (1.9–5.1)	4.9 (2.7–7.0)	5.7 (3.2–7.1)	5.0 (2.7–6.7)	6.5 (3.0–8.8)	3.9 (2.2–4.8)	3.4 (1.9–4.3)	4.4 (2.0–5.9)

*Note*: Data in parenthesis are 5% UI.

National total ASIR has increased by 29.6% (−25.3–57.5); female ASIR by 42.6% (−25.0–139.2); and male ASIR by 21.2% (−29.3–64.1). Although national total and male ASDR results showed a slight decrease in the abovementioned period (total: −1.1% (−43.8–22.9); male: −8.7% (−45.2–22.3)), female ASDR rate has increased in this interval (10.3% (−41.3–75.0)) (Table 2).

**TABLE 2:** Percent change in the age‐standardized incidence, deaths, and DALYs rates at national and subnational levels from 1990 to 2019, by sex

Location		Incidence (%)			Deaths (%)			DALYs (%)		
		T	F	M	T	F	M	T	F	M
Iran (Islamic Republic of)		29.6 (−25.3–57.5)	42.6 (−25.0–139.2)	21.2 (−29.3–64.1)	−1.1 (−43.8–22.9)	10.3 (−41.3–75.0)	−8.7 (−45.2–22.3)	−10.7 (−50.1–8.5)	−2.4 (−51.1–67.9)	−16.1 (−52.0–14.1)
Subnational	Alborz	49.8 (−9.6–123.2)	58.2 (−24.8–219)	45.3 (−17.5–137.5)	2.6 (−37.1–56.2)	9.5 (−44.7–108.3)	−1.2 (−41.4–65.0)	−5.7 (−43.1–37.8)	0.7 (−52.5–104.8)	−9.4 (−47.9–49.4)
	Ardebil	46.0 (−27.2–118.7)	53.3 (−36.0–215.9)	44.2 (−31.1–147.2)	16.0 (−40.4–70.2)	21.0 (−47.2–133.7)	15.6 (−42.8–93.4)	−2.9 (−55.2–45.0)	1.8 (−60.3–110.7)	−4.6 (−57.5–69.5)
	Bushehr	31.3 (−21.6–86.6)	44.7 (−28.5–175.0)	22.0 (−29.7–95.6)	−0.6 (−39.1–39.1)	9.8 (−42.4–96.1)	−7.8 (−44.8–44.0)	−10.3 (−48.7–30.2)	−1.6 (−52.8–94.2)	−16.5 (−53.3–35.6)
	Chahar Mahaal and Bakhtiari	19.7 (−26.9–66.7)	32.4 (−44.1–158.7)	14.5 (−35.4–87.6)	−15.1 (−47.0–18.2)	−6.5 (−60.7–75.1)	−18.3 (−51.4–25.5)	−22.2 (−54.6–12.2)	−14.9 (−66.1–75.5)	−25.3 (−58.7–23.6)
	East Azerbayejan	18.3 (−24.9–60.8)	36 (−32.3–157.5)	7.6 (−34.4–69.5)	−8.7 (−42.3–23.2)	4.9 (−44.4–85.0)	−16.7 (−50.2–27.9)	−20.3 (−52.6–9.7)	−10.2 (−56.7–78.0)	−26.8 (−58.9–18.9)
	Fars	55.0 (−13.6–120.2)	68.0 (−17–212.2)	46.2 (−25.8–139.6)	12.3 (−36.5–65.0)	25.2 (−35.5–129.3)	3.6 (−45.8–74.7)	3.1 (−40.2–48.4)	9.6 (−44.1–111.8)	−1.2 (−48.5–61.6)
	Gilan	53.8 (−14.8–123.3)	67.3 (−20.0–205.5)	43.8 (−25.9–133.5)	16.6 (−35.6–69.8)	30.5 (−36.0–135.8)	6.2 (−45.7–76.4)	4.8 (−41.9–49.5)	14.0 (−46.5–117.0)	−1.7 (−49.7–61.1)
	Golestan	33.5 (−16.7–90.3)	42.4 (−23.8–151.2)	28.8 (−25.3–100.6)	6.6 (−32.4–50.7)	15.6 (−35.8–93.1)	1.6 (−37.2–55.2)	−3.2 (−42.1–36.2)	2.3 (−47.4–86.1)	−6.2 (−46.2–45.3)
	Hamedan	30.0 (−22.4–81.8)	39.9 (−32.3–162.6)	24.3 (−32.7–105.1)	30.0 (−22.4–81.8)	9.7 (−42.6–94.2)	−4.2 (−47.7–54)	−10.4 (−49.3–22.8)	−4.7 (−53.7–81.1)	−13.8 (−54.9–40.0)
	Hormozgan	34.5 (−29.2–119.2)	47.9 (−39.6–207.2)	26.1 (−30.7–124.9)	2.7 (−46.7–64.9)	16.6 (−49.5–127.7)	−6.4 (−51.3–71.9)	−4.1 (−51.3–76.9)	4.5 (−59.8–131.7)	−9.7 (−53.8–79.3)
	Ilam	42.3 (−16.5–96.2)	59.2 (−21.3–228.4)	35.0 (−21.9–110.6)	5.9 (−35.8–50.1)	23.7 (−37.2–143.6)	−2.7 (−41.1–51.6)	−3.3 (−45.2–34)	6.9 (−49.5–129.3)	−8.4 (−48.5–44.2)
	Isfahan	49.2 (−13.9–114.6)	64.5 (−12.5–206.3)	37.7 (−25.1–120.9)	13.1 (−34.4–65.0)	26.5 (−32.9–131.2)	2.7 (−46.2–69.0)	1.6 (−41.0–44.4)	11.5 (−42.3–115.5)	−5.6 (−51.8–50.6)
	Kerman	17.9 (−26.5–60.1)	35.1 (−28.0–136)	6.7 (−41.4–71.3)	−7.8 (−43.7–23.8)	6.4 (−44.2–83.4)	−16.9 (−53.8–32.5)	−13.3 (−48.2–19.5)	−2.7 (−50.6–82.6)	−20.5 (−57.2–28.5)
	Kermanshah	13.2 (−29.2–59.3)	28.8 (−35.6–151.8)	6.2 (−39.5–70.1)	−13.5 (−46.1–21.7)	0.9 (−48.1–87)	−19.9 (−53.2–30.7)	−19.3 (−51.6–16.5)	−10.7 (−56.8–79.5)	−23.4 (−56.3–29.4)
	Khorasan‐e‐Razavi	22.9 (−33.3–73.1)	38.8 (−35.1–161.5)	14.2 (−47.3–87.2)	−3.9 (−50.2–33.6)	8.0 (−48.2–95.2)	−10.2 (−56.3–40.8)	−15.3 (−58.7–20.1)	−6.4 (−59.4–85.4)	−20.6 (−64.5–30.5)
	Khuzestan	57.8 (−11.1–119.8)	73.4 (−9.2–216.7)	47.9 (−23.7–137.3)	19.4 (−34.0–67.8)	31.5 (−30.1–133.4)	11.6 (−43.0–82.5)	13.1 (−38.3–58.8)	24.4 (−34.4–126.3)	5.9 (−46.7–70.3)
	Kohgiluyeh and Boyer‐Ahmad	61.7 (−10.5–137.7)	77.2 (−18.2–284.2)	52.9 (−25.6–164.3)	11.3 (−38.9–62.6)	21.6 (−41.7–140.9)	5.4 (−50.2–79.9)	2.0 (−45.6–49.3)	12.0 (−49–138.3)	−3.3 (−55.8–68.4)
	Kurdistan	10.3 (−40.8–58.1)	22.4 (−48.3–143.3)	3.9 (−44.0–70.9)	−12.0 (−50.9–18.5)	1.6 (−54.7–86.8)	−19.4 (−56.9–29.5)	−23.4 (−60.8–11.4)	−17.4 (−67.1–79.4)	−26.8 (−62.9–23.9)
	Lorestan	35.0 (−25.1–94.3)	45.9 (−38.9–181.3)	31.2 (−28.5–114.9)	2.2 (−41.0–46.9)	10.0 (−50.9–113.9)	−0.2 (−46.2–65.2)	−8.4 (−50.8–31.4)	−0.7 (−59.7–104.1)	−11.5 (−56.1–51.8)
	Markazi	15.6 (−32.4–59.3)	24.9 (−38.7–140.5)	9.9 (−46.4–75.9)	−12.6 (−48.5–17.5)	−4.5 (−50.9–73.6)	−17.5 (−59.2–28.8)	−19.6 (−54–13.5)	−15.3 (−58.5–66.5)	−22.5 (−63.3–23.4)
	Mazandaran	71.1 (−4.8–142.5)	84.1 (−4.2–252)	61.8 (−18.7–168.5)	23.4 (−31.7–75.3)	33.8 (−30.9–147.5)	15.6 (−39.5–87.9)	14.2 (−37.1–57.2)	23.6 (−36.9–136.7)	7.3 (−44.8–72.6)
	North Khorasan	155.1 (26.2–294.3)	56.1 (−27.3–200.7)	21.9 (−39.9–105.3)	6.5 (−44.4–50.8)	23.6 (−40–134.4)	−4.4 (−51.0–59.2)	−3.1 (−51.9–39.9)	10.5 (−51.3–124.8)	−12.4 (−57.7–49.7)
	Qazvin	51.1 (−23.1–118.8)	55.1 (−34.9–197.3)	49.3 (−25.9–137.8)	17.7 (−42.2–65.8)	20.8 (−47.1–123)	16.2 (−43.5–86)	1.5 (−52.9–41.2)	2.6 (−57.5–104.9)	1.4 (−55.1–67.6)
	Qom	24.2 (−31.0–91.0)	32.3 (−36.9–158.3)	18.4 (−39.4–99.1)	−6.3 (−44.9–36.8)	2.6 (−48–90.0)	−12.5 (−55.0–42.1)	−13.6 (−53.2–35.8)	−7.8 (−57–93.5)	−18 (−59.7–41.9)
	Semnan	26.1 (−23.0–79.1)	40.5 (−33.0–187.2)	16.0 (−30.8–79.9)	−5.8 (−39.7–29.9)	5.1 (−47.1–102.7)	−13.7 (−49.0–31.8)	−14.9 (−48.5–18.7)	−5.5 (−54.7–96.4)	−21.4 (−56.8–25.4)
	Sistan and Baluchistan	28.4 (−24.8–144.8)	51.9 (−19.8–220.4)	18.9 (−36.4–154.8)	11.4 (−34.6–103.1)	30.8 (−30.6–148.9)	3.9 (−45.3–115)	3.8 (−40.0–131.9)	23.3 (−40.0–187.3)	−4.9 (−51.9–144.4)
	South Khorasan	30.0 (−33.2–85.1)	50 (−34.0–196.9)	18.7 (−43.9–101.7)	1.6 (−44.8–40.4)	16.6 (−44.6–119.6)	−6.9 (−52.7–54.5)	−8.5 (−54.1–30.7)	6.4 (−54.4–112.6)	−17.5 (−61.2–44.8)
	Tehran	8.9 (−30.0–49.8)	17.8 (−41.5–141.9)	2.1 (−38.4–63.7)	−16.2 (−45.9–14.2)	−7.2 (−52.8–81.5)	−23.4 (−53.4–21.6)	−22.8 (−50.8–8.6)	−15.8 (−58.3–74.1)	−28.1 (−56.3–16.4)
	West Azerbayjan	25.1 (−30.2–69.8)	39.8 (−31.3–159.9)	16.5 (−38.9–81.7)	−0.2 (−41.9–33.5)	12.9 (−39.2–97.8)	−7.7 (−49.4–40.6)	−14.6 (−51.3–13.6)	−7.2 (−53.3–73.3)	−19.2 (−57.9–26.4)
	Yazd	45.3 (−13.2–101.0)	55.9 (−18.3–185.8)	35.0 (−24.6–113.7)	−0.4 (−38.8–36.4)	8.6 (−40.4–88.1)	−9.1 (−47.3–41.9)	−7.6 (−44.5–26.1)	−1.7 (−48.0–81.9)	−13.2 (−51.0–37.4)
	Zanjan	18.2 (−31.8–57.8)	28.4 (−40.1–147.0)	12.9 (−36.7–73.8)	−6.9 (−41.4–22.9)	1.5 (−47.0–79.5)	−11.4 (−47.4–33.9)	−19.9 (−55.3–7.8)	−13.8 (−60.5–72.3)	−23.2 (−58.7–22.7)

*Note*: Data in parenthesis are 5% UI.

**FIGURE 1:**
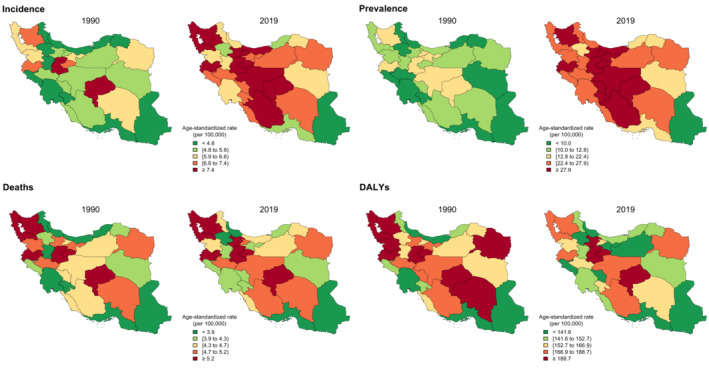
The subnational age‐standardized rates of epidemiological measures in 1990 and 2019, both sexes.

Subnational results revealed an interesting pattern; in 1990 and 2019, Sistan and Baluchistan had the lowest ASIR (**1990: total**: 3.0 (1.5–5.8); **2019: total**: 3.9 (3.1–5.1)); and ASDR (**1990: total**: 2.7 (1.5–5.2); **2019:** total: 3.0 (2.4–4.0)) among all provinces, both in total and gender‐specific scopes. On the other hand, Yazd had the highest ASIR (**total**: 11.1 (3.9–15.5)) and ASDR (**total**: 6.6 (2.2–9.2)) in 2019 in total and gender‐specific results. In 1990, Yazd again had the highest ASDR in total and gender‐specific results (**total**: 6.6 (2.7–9.3)) and the highest total ASIR (7.6 (3.1–10.8)). However, in 1990, Ardebil had the highest ASIR in females (7.2 (3.3–15.7)) and males (10.6 (5.7–18.4), but this pattern is not observed in 2019 results (Table 1, Figure 1, Figure S3A, B). The gap between subnational highest and lowest ASIRs has increased from 4.6 in 1990 to 7.2 in 2019, while the gap for ASDR has remained almost the same in 1990 and 2019.

**FIGURE 2:**
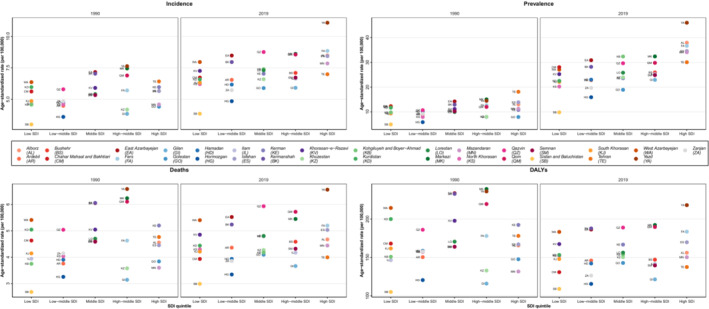
The subnational age‐standardized rates of epidemiological measures by socio‐demographic index (SDI) in 1990 and 2019, both sexes.

**FIGURE 3:**
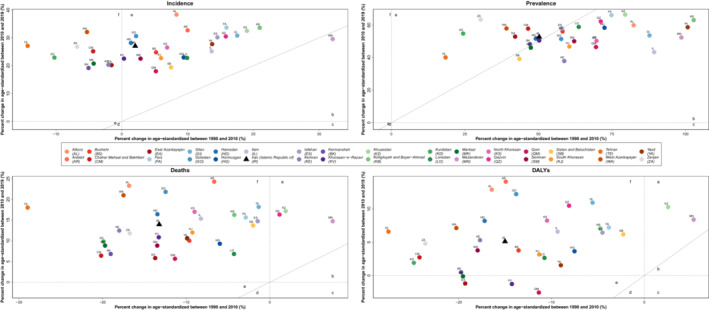
The distribution of national and subnational changes in age‐standardized rates of epidemiological measures, 1990 to 2010 vs 2010 to 2019, both sex.

ASR estimates for each SDI group revealed an increased gap between the highest and lowest ASPRs of provinces in the same SDI group in 2019 compared with 1990. Moreover, in the high‐middle SDI quantiles, the gap between provinces for ASDR has increased in 2019 compared with 1990 (Figure 2). Even though the ASIR rates of CNS cancers showed an increasing pattern during the 1990–2019 interval in total and gender‐specific results, ASDR patterns were less uniform. Subnational changes in ASDR and ASIR showed a uniform increasing pattern after 2010, with some provinces showing an accelerated increase after 2010 vs. before 2010 (Figure 3, region a) and some provinces showing an inverted pattern where the rate was decreasing before 2010 but has increased since 2010 (Figure 3, region f). Sex‐specific trends were similar for ASIR rates (Figure S4A) but showed more diversity in ASDR rates (Figure S4B).

The decomposition analysis of new cases at the national level revealed that 53.6% of 130.3% increase in CNS Cancers incident cases from 1990 to 2019 was due to a rise in the age‐specific incidence rate. Population growth and population aging accounted for 44.0% and 32.7% increase, respectively. The same pattern was observed for females (152.7%; Incidence rate change: 74.6%; Population growth: 44.9; Age structure change: 33.1%) but not males (114.4%; Incidence rate change: 40.0%; Population growth: 43.1; Age structure change: 31.3%). Decomposition results were more variable on the subnational scale, with some provinces showing the dominance of age‐specific incidence rate change (e.g., Alborz), age structure change (e.g., East Azerbayejan), or population growth (e.g., Hormozgan) toward the overall change in new cases. An interesting finding in gender‐specific subnational decomposition results was the negative impact of changes in male age structure on the percentage of new cases in Sistan and Baluchistan (Table 3, Table S2).

**TABLE 3:** Decomposition analysis of CNS cancers new cases between 1990 and 2019 at national and subnational levels, by sex

Location		1990–2019 new cases change cause									1990–2019 new cases overall change (%)		
		Population growth (%)			Age structure change (%)			Incidence rate change (%)					
		T	F	M	T	F	M	T	F	M	T	F	M
Iran (Islamic Republic of)		44.0	44.9	43.1	32.7	33.1	31.3	53.6	74.6	40.0	130.3	152.7	114.4
Subnational	Alborz	95.4	99.0	92.0	59.3	71.9	49.8	127.8	149.5	116.0	282.4	320.5	257.9
	Ardebil	10.9	10.9	10.8	19.3	24.1	14.2	62.6	73.6	53.8	92.8	108.5	82.8
	Bushehr	71.4	64.7	78.0	45.5	44.9	44.3	67.0	93.7	46.6	183.9	203.3	168.9
	Chahar Mahaal and Bakhtiari	35.3	36.0	34.8	36.4	36.2	33.8	36.8	58.8	26.4	108.6	130.9	94.9
	East Azarbayejan	18.2	18.1	18.2	27.7	27.1	26.0	26.0	49.3	11.8	72.0	94.5	56.0
	Fars	35.9	36.5	35.4	34.3	29.0	37.1	95.5	110.3	85.7	165.7	175.8	158.1
	Gilan	11.6	11.7	11.4	46.1	46.5	46.2	85.1	104.4	71.7	142.8	162.7	129.3
	Golestan	43.9	44.3	43.5	33.2	36.9	28.6	59.0	75.6	49.7	136.1	156.8	121.9
	Hamadan	3.4	5.1	1.8	22.9	21.7	22.8	41.2	52.5	34.5	67.5	79.4	59.1
	Hormozgan	105.0	105.1	104.9	24.2	32.0	16.0	88.2	115.9	70.7	217.4	253.0	191.6
	Ilam	32.0	33.7	30.4	33.4	35.0	28.5	77.9	101.8	66.9	143.4	170.5	125.8
	Isfahan	37.1	39.7	34.6	37.2	34.7	39.7	87.1	111.2	69.5	161.4	185.6	143.8
	Kerman	78.3	76.0	80.5	36.3	35.7	34.4	41.7	74.3	19.7	156.3	186.0	134.7
	Kermanshah	16.3	18.7	14.1	40.0	40.6	35.9	21.0	42.4	12.3	77.4	101.7	62.3
	Khorasan‐e‐Razavi	41.4	41.7	41.1	19.2	17.8	18.1	37.0	61.3	22.6	97.6	120.7	81.8
	Khuzestan	52.2	53.2	51.3	34.8	35.4	33.3	113.7	142.7	94.8	200.7	231.2	179.3
	Kohgiluyeh and Boyer‐Ahmad	51.9	51.9	52.0	16.1	14.6	16.1	116.2	141.8	101.4	184.3	208.3	169.5
	Kurdistan	33.8	34.2	33.5	22.3	21.2	20.1	18.4	34.3	10.1	74.5	89.7	63.7
	Lorestan	14.3	15.5	13.2	38.9	46.0	31.1	56.5	76.5	47.6	109.7	138.0	91.9
	Markazi	19.2	18.5	19.9	31.7	30.4	31.5	26.6	36.3	20.8	77.5	85.2	72.1
	Mazandaran	31.5	31.3	31.6	49.9	53.6	46.4	128.9	149.3	114.7	210.3	234.1	192.8
	North Khorasan	37.5	37.6	37.3	25.6	28.5	21.2	58.8	91.7	37.4	121.9	157.8	95.9
	Qazvin	39.9	40.4	39.3	36.4	36.8	34.7	92.7	98.0	89.8	169.0	175.3	163.9
	Qom	84.1	85.8	82.4	50.6	49.0	51.0	59.5	75.2	47.9	194.2	210.1	181.3
	Semnan	54.9	57.1	52.9	36.4	37.0	36.3	51.5	79.2	35.2	142.8	173.3	121.7
	Sistan and Baluchistan	101.9	102	101.9	1.9	13.5	−6.8	52.8	112.7	25.5	156.6	228.2	120.5
	South Khorasan	24.2	24.1	24.4	11.7	19.7	4.8	43.2	73.5	26.1	79.1	117.2	55.3
	Tehran	63.8	67.3	60.5	35.6	37.7	33.6	16.7	33.6	4.7	116.1	138.6	98.8
	West Azarbayejan	46.6	46.8	46.5	23.9	19.8	24.5	39.3	59.9	26.3	109.8	126.4	97.3
	Yazd	69.2	70.3	68.1	37.6	32.5	44.4	97.3	115.4	79.9	204.1	218.2	192.4
	Zanjan	21.6	22.1	21.0	28.6	28.7	27.1	29.0	44.1	20.2	79.1	94.9	68.3

### 3.2 Age distribution

**FIGURE 4:**
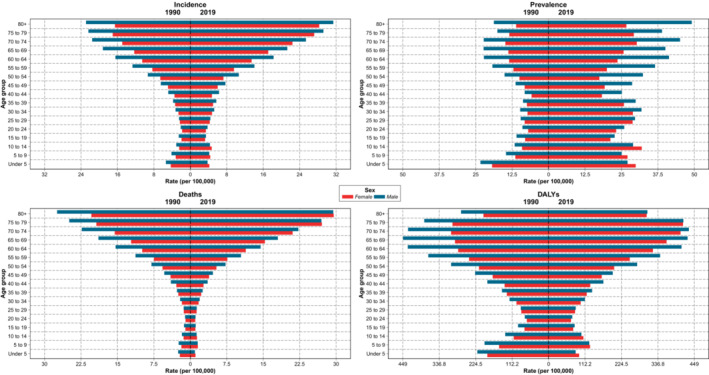
The rates of epidemiological measures by age composition at the national level in 1990 and 2019; the plot is further decomposed based on sex.

Regarding the age composition of CNS cancers, new and prevalent cases have increased in all age groups and genders since 1990. On the other hand, mortality rates have remained the same for most age groups and genders, with the exception of young patients aged under 15, where a decrease can be observed. The general age and gender pattern of incidence and expiration has mostly remained the same between 2019 and 1990. The prevalence pyramid, however, shows more structural changes in this interval (Figure 4, Figure S5).

In 2019, patients older than 55 accounted for 38.5% of all new and 17% of all prevalent cases. Consistently, almost 55.8% of CNS cancers mortality was among patients aged 55 or more. Incidence changes were more prominent during the past 30 years in patients over 55, where the rate was increased by 29.2%. Moreover, mortality rates have also increased by 15.1% (Figure 4).

### 3.3 YLLs, YLDs, and DALYs

**FIGURE 5:**
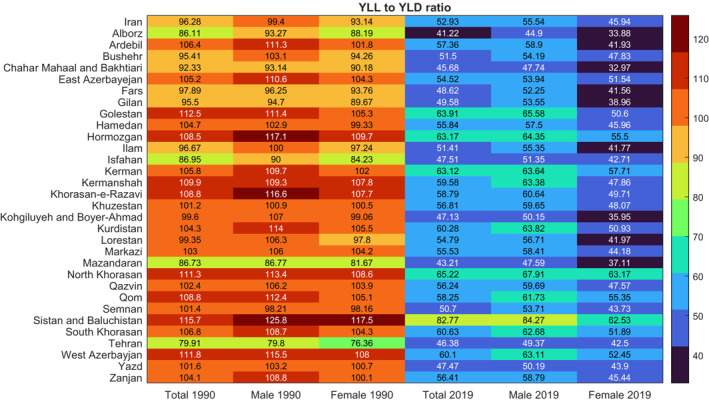
Subnational YLLs to YLDs ratios in 1990 and 2019, both sexes.

**FIGURE 6:**
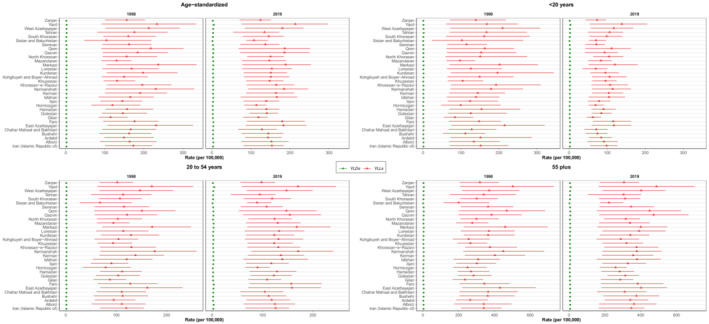
Subnational distribution of YLLs and YLDs rates. The diagrams are plotted in age‐standardized, under 20, 20–54, and above 55 modes.

On the national level, overall DALYs number of CNS cancers increased, albeit not statistically significant, by 40.5% (−27.9–81.4) from 1990 to 2019. In addition, 33.4% of the DALYs data occurred for patients aged ≥55 years. The increasing trend of DALYs is consistent with YLLs and YLDs with a 39.2% (−28.5–80.0) and 174.0% (49.3–259.1) increase since 1990, which implies a steeper slope for YLDs. The aforementioned dynamics can be observed in the decreasing course of YLL to YLD ratios over the 1990–2019 period in all provinces, pointing to a prominent increase in YLD rates (Figure 5). Despite this observation, however, the majority of DALYs still come from YLLs (Figure 6).

ASR DALYs for CNS cancers revealed a national rate of 156.4 (82.0–187.0) in the total population with a female‐specific rate of 147.0 (76.0–184.9) and a male rate of 165.7 (72.9–213.1) per 100,000 population in 2019. Subnational results showed that 11 provinces had a higher ASR of DALYs compared with the national results; Yazd had the highest value, 218.1 (77.1–302.9). Furthermore, compared with national values, gender‐specific subnational results revealed that 12 provinces had higher DALYs rates for females and 15 for males. In both genders, Yazd had the highest rates (Male: 220.8 (64.7–345.1); Female: 215.1 (74.6–310.3)) (Table 4).

**TABLE 4:** The age‐standardized DALYs rate at national and subnational levels in 1990 and 2019, by sex

Location		1990			2019		
		DALYs (Per 100,000 population)			DALYs (Per 100,000 population)		
		T	F	M	T	F	M
**Iran (Islamic Republic of)**		175.0 (102–223.7)	150.6 (70.4–246.8)	197.6 (104–275.6)	156.4 (82.0–187)	147 (76–184.9)	165.7 (72.9–213.1)
**Subnational**	Alborz	165.5 (88.5–235.8)	141.4 (66.8–268.0)	187.3 (88.9–303.4)	156.1 (76.8–199.8)	142.3 (67.4–186.9)	169.8 (64.6–238.7)
	Ardebil	150.4 (100.0–227.6)	123.5 (63.9–246.7)	174.6 (98.3–284.1)	146 (86.6–181.3)	125.8 (78–159)	166.5 (79.9–224.9)
	Bushehr	163.9 (87.2–221.1)	145.8 (62–244.7)	180.9 (86.7–267.5)	147.1 (77.4–185.6)	143.5 (75.5–193.5)	151 (67.7–208.1)
	Chahar Mahaal and Bakhtiari	168.0 (94.6–234)	131.8 (54.9–229.7)	200.6 (95.9–316.0)	130.8 (69.5–169.7)	112.1 (59.4–162.8)	149.8 (64.4–218.4)
	East Azerbayejan	233.7 (107.6–329.9)	200.9 (69.9–341.6)	263.2 (103.8–409.3)	186.3 (87.7–245.3)	180.4 (79.1–255.3)	192.6 (77.6–278.1)
	Fars	178 (97.1–239.8)	155.5 (65.2–253.1)	199.0 (95.8–293.7)	183.6 (91.9–241.8)	170.4 (77.7–234.7)	196.6 (86.6–282.3)
	Gilan	115.9 (85.2–160.3)	95.7 (57.0–151.0)	136.0 (91.4–207.4)	121.4 (81.4–145.7)	109.1 (73.0–141.4)	133.8 (73.0–177.0)
	Golestan	147.6 (93.6–209.8)	123.6 (67.8–206.4)	170.1 (91.3–276.0)	142.8 (97.2–171.7)	126.5 (85.3–158.8)	159.6 (88.9–207.3)
	Hamedan	158.6 (102.9–225.7)	135.1 (64.7–233.3)	180.5 (97.8–291.9)	142.1 (89.8–175.7)	128.7 (78.6–166.1)	155.6 (86.3–210.8
	Hormozgan	120.5 (66.1–192.9)	106.3 (49.8–197.0)	132.8 (70.0–243.0)	115.5 (84.2–140.5)	111.0 (71.6–146.3)	119.8 (79.1–159.9)
	Ilam	146.4 (90.3–197.2)	121.2 (54–210.9)	167.0 (89.1–248.3)	141.5 (83.1–175.0)	129.5 (76.7–169.4)	153 (75.6–204.3)
	Isfahan	167.0 (85.4–228)	145.6 (65.6–227.0)	187.5 (85.4–289.4)	169.8 (75.2–222.5)	162.3 (68.9–221.9)	177.0 (63.4–255.6)
	Kerman	192.3 (102.3–261.8)	166.0 (67.1–277.7)	216.3 (100.3–332.4)	166.7 (89.4–213.2)	161.6 (76.8–217.1)	171.9 (81.6–242.0)
	Kermanshah	232.8 (103.9–331.2)	187.5 (66.8–321.4)	271.9 (101–434.5)	187.8 (94.0–250.8)	167.5 (78.5–234.6)	208.4 (86.9–310.5)
	Khorasan‐e‐Razavi	197.7 (106.8–273.4)	164.6 (66.5–295.6)	228.3 (101.7–355.9)	167.4 (90.5–214.2)	154.1 (79.7–212.6	181.2 (77.8–254.8)
	Khuzestan	132.9 (98.0–180.1)	112.1 (68.5–176.5)	152.2 (97.5–228)	150.3 (89.3–186.5)	139.4 (82.0–181.6)	161.2 (78.5–217.4)
	Kohgiluyeh and Boyer‐Ahmad	150.9 (93.7–212.4)	118.8 (55.4–209.8)	180 (91.9–283.6)	153.9 (83.5–201.6)	133.0 (79.6–181.5	174 (72.7–252.9)
	Kurdistan	200.0 (106.0–288.5)	172.5 (61.0–342.0)	223.6 (101.5–356.6)	153.2 (82.3–195.3)	142.6 (73.0–193.1)	163.7 (77.4–229.2)
	Lorestan	170.5 (94.8–240.0)	139.5 (62.2–254.1)	197.6 (95.2–314.2)	156.3 (78.8–208.8)	138.5 (64.5–196.0)	174.9 (75.8–259.1)
	Markazi	239.1 (105.5–338.2)	203.4 (70.2–353.8)	273.5 (102.5–431.0)	192.2 (78.8–258.4)	172.3 (71.3–238.6)	212.0 (70.3–318.0)
	Mazandaran	131.6 (92.4–170.3)	114.0 (65.5–181.6)	148.8 (92.5–212.5)	150.3 (79.3–193.5)	141.0 (72.1–188.3)	159.7 (69.3–224.5)
	North Khorasan	157.2 (95.4–236.6)	137.2 (62.1–269.5)	175.2 (95.8–305.6)	152.3 (91.6–187)	151.6 (82.9–197.9)	153.5 (84.1–201.8)
	Qazvin	186.0 (95.8–263.9)	171.5 (61.5–331.8)	199.4 (91.6–307.6)	188.9 (74.1–253.8)	176.0 (65.3–246.5)	202.2 (67.1–297.6)
	Qom	219.6 (92.2–304.8)	204.2 (67.3–355.8)	233.4 (89.9–369.8)	189.7 (69.6–255.7)	188.2 (66.1–264.6)	191.5 (56.6–286.4)
	Semnan	163.9 (95.2–220.5)	138.9 (62.4–232.5)	188.4 (93.2–285.9)	139.6 (73.5–175.9)	131.2 (69–174.3)	148.1 (68.4–201.5)
	Sistan and Baluchistan	105.0 (47.7–221.0)	76.1 (31.6–163.1)	130.4 (54.5–299.2)	109.0 (89.2–144.8)	93.8 (70.8–134.8)	124.1 (89.6–173.9)
	South Khorasan	161.7 (97.7–232.9)	131.6 (62.1–244.2)	189.5 (96.5–311.1)	147.9 (80.2–185.9)	140.1 (75.5–188.1)	156.3 (70.2–214.3)
	Tehran	178.1 (81.7–263.5)	161.5 (80.4–307.4)	193.4 (68.9–326.6)	137.5 (55.5–175.3)	136.0(60.0–176.0)	139.0 (37.4–197.2)
	West Azerbayjan	214.5 (102.7–94.9)	186.5 (68.7–314.5)	239.9 (101.0–365.6)	183.3 (85.0–238)	173.1 (80.5–239.4)	193.8 (77.9–275.9)
	Yazd	236.0 (92.8–337.1)	218.8 (70.9–372.8)	254.2 (88.8–400.6)	218.1 (77.1–302.9)	215.1 (74.6–310.3)	220.8 (64.7–345.1)
	Zanjan	157.7 (101.7–204.3)	131.8 (66.5–227.4)	182.0 (99.8–263)	126.3 (72.1–154.2)	113.6 (64.2–149.3)	139.8 (63.7–186.6)

*Note*: Data in parenthesis are 5% UI.

ASR of DALYs trend displayed a steady pattern (Figure S5) with a percent change of −10.7% (−50.1–8.5) for the total population, −2.4% (−51.1–67.9) for females and − 16.1% (−52.0–14.1) for males between 1990 and 2019 (Table 2). Subnational results in the whole population showed that four provinces, East Azerbayejan, Khorasan‐e‐Razavi, Markazi, and Qom, had strictly decreasing DALYs during the past 30 years, although the slope has become blunted after 2010 compared with the 1990–2010 slope (Figure 3). Gender‐specific results, however, displayed a different pattern; only Qom had a decreasing DALYs rate in the female population while several provinces showed a decreasing rate in males (Figure S4).

ASR DALYs rates showed prominent changes in the under 15 population, in both males and females, where the rates decreased in 2019 compared with 1990 in most gender‐age groups. Moreover, individuals aged over 55 were the main contributors toward total national DALYs (Figure 4). The abovementioned pattern is also observable in subnational results (Figure S5). Gender‐specific results revealed that while female DALYs in all provinces, except Qom, still had a positive percent of change after 2010 compared with the 1990–2010 interval, the percent of change for male DALYs was negative in several provinces, such as Yazd, both before and after 2010 (Figure S6). Regarding further decomposition of DALYs results, the most contributing factor was YLLs, both in 1990 and 2019, with YLDs contributing slightly (Figure 6, Table S3). In spite of this observation, the YLDs have increased since 1990, as observed in the decreased YLLs to YLDs ratios in all provinces and both genders. Furthermore, Sistan and Baluchistan had the highest total YLLs to YLDs ratio both in 1990 and 2019. Further investigation of age structures revealed that, compared with 1990, in 2019, a significant YLLs decrease is observable in almost all provinces for patients under 20, while results for individuals aged 20–54 or older show only slight changes.

## 4 DISCUSSION

This study investigated the burden of CNS cancers in Iran at national and subnational levels. It was observed that the national burden of CNS cancers, conveyed by ASIR, ASDR, and ASPR rates, has been increasing during the past 15 years in both males and females. On the other hand, DALYs rates have slightly declined compared with rates in 1990. Our results are consistent with findings of previous GBD studies where global age‐standardized incidence rates for CNS cancers were increasing, but DALYs were decreasing.^1^, ^2^ Moreover, contrary to the results of these studies, the pattern mentioned above is observed in all Iranian province SDI groups. The increase in incidence is more prominent in high SDI regions, but the DALYs rate remains comparable to low SDI regions. The increase in incidence is most likely due to a general advancement of medical science and system in the country.^25^ Since patients in high SDI regions have access to more specialized health centers, the disease is more likely to be detected, and better treatment options are available. As a result, even though the incidence of CNS cancers shows a higher increase in high SDI regions, the DALYs rate remains similar to low SDI provinces, affirming previous results that CNS cancer prognosis is significantly affected by economic status.^26^

Although the gap between the highest and lowest ASIR among provinces has increased since 1990, the gap for death rates has remained approximately the same. In 2019, Sistan and Baluchistan had the lowest ASDR and ASIR among provinces, while Yazd had the highest rates for both males and females. The observation that Sistan and Baluchistan had the lowest ASIR and ASRD in both 1990 and 2019 is most likely due to the low socioeconomic status of this province, causing poor patient care and high patient missing rate. This can further be affirmed by the observed high rate of YLLs to YLDs ratio in this province. CNS cancers impose a significant burden on healthcare systems even though they are not among the top prevalent cancers of the human body. This seemingly paradoxical observation is due to the complicated nature of the disease and currently available treatments. Highly specialized medical and surgical interventions are required for diagnosis and long‐term management of the disease, where multidimensional approaches with a combination of biopsy, invasive surgical resection, radiotherapy, and chemotherapy are often required.^27^ Therefore, for proper treatment, access to advanced neurosurgical teams, intensive care units, and professional radiation and neuro‐oncologists is required, all of which are primarily located in urban and affluent areas with advanced medical services.^28^ Furthermore, low incidence of neoplasms such as CNS tumors, compared with other cancers, results in an attributed low priority in regions with limited resources, decreasing the probability of early diagnosis and patient survival. The aforementioned factors are supported by reports that improved socioeconomic status of a region causes an improvement in the prognosis of CNS and other organ system cancers.^29^, ^30^, ^31^

While gender decomposition of ASIRs revealed highly similar patterns of change for males and females over time, this pattern was more distinct in ASDR results, with males showing accelerated rates after 2010 compared with the 1990–2010 interval. Furthermore, dissimilarities were present for the DALYs percent of change results, when trends were compared after and before 2010, between females and males. Gender disparities are present in the incidence and mortality of cancers and other non‐communicable diseases. Overall, males are more prone to developing cancers and show more inadequate responses to treatments.^32^, ^33^, ^34^ Furthermore, Glioma, a frequent CNS cancer in adults, is more likely to occur in men and is accompanied by distinct oncogenic mechanisms.^35^ Moreover, gender disparities are also observed in pediatric CNS Cancers.^2^, ^36^ Observed disparities in different ASR trends in this study are probably from a mixture of aforementioned factors along with the improved social and economic status of females in Iran in the past 20 years.^2^

While a significant portion of CNS cancers mortality in Iran still comes from individuals older than 50, ASDR rates have decreased in the under 15 population in 2019 compared with 1990 in males and females on national as well as subnational data. Moreover, DALY rates decreased in 2019 compared with 1990 overall and in most subnational regions. Further decomposition revealed that YLLs comprise a significant portion of DALYs caused by CNS cancers, and they show changes similar to DALYs rates; YLLs have decreased for patients under 20 while remaining largely unchanged for other age groups. These results are consistent with a previous GBD study investigating the global burden of CNS cancers^1^ and with reports indicating survival has improved in younger individuals with most histological subtypes of CNS tumors in recent years.^37^, ^38^ Moreover, the majority of DALYs are from YLLs because even with advanced treatments, the course of CNS cancers, especially in adults, is often poor, and significant mortality is observed in these patients. Several initial symptoms of the disease, such as headache and seizures, are common and non‐specific. Along with this, the lack of a solid screening protocol adds to the complexity of early diagnosis of CNS cancers,^39^ and late discovery of the disease often leads to poor prognosis.^9^

One key point to note is the role of environmental variables and risk factors which are not yet completely clear for CNS cancers, and further studies are needed in this regard.^40^ These factors can cause some of the observed disparities in the burden of CNS cancers between different regions. Although a positive association between CNS cancers and ionizing radiation has been established,^41^, ^42^ the role of other potentially hazardous factors, such as mobile phone radiations, is not yet completely clarified. More studies are needed to discover other risk factors for CNS cancers and the degree to which environmental factors contribute to the incidence of CNS cancers.^1^

### 4.1 Strengths and limitations

The GBD data provide one of the most accurate estimates for the incidence, death, and burden of all cancers. Furthermore, for the first time, GBD 2019 has released subnational data from 21 countries, which provides the opportunity to assess cancer epidemiology aspects in different regions and provinces within a country. Despite all these strengths, the limitations of GBD studies cannot be overlooked. The GBD estimations, similar to the other estimation groups such as GLOBOCAN, primarily rely on the quality and quantity of modeling data. The incongruent variability of high‐ and low‐quality causes of death and cancer registry data are reflected in 95% uncertainty intervals. Moreover, the reliability of cancer registry data, especially those from low socioeconomic regions, is questionable. This is one of the main reasons for several GBD modeling processes, including redistribution of miscoded deaths, garbage codes, and modeling based on mortality trends. Considering the risk factor analysis of GBD studies, the data on risk factors of CNS cancers are not reported, mainly because these factors are not yet totally uncovered and understood well. More studies are needed to unravel possible regional and global risk factors for CNS cancers.

## 5 CONCLUSION

In this nationwide epidemiological study, metrics and the burden of CNS cancers over a 30‐ year period in Iran were discussed. Incidence, mortality, and prevalence of CNS cancers increased in the past 15 years, but DALYs showed a slight decrease, a trend also observed on a global scale. The DALYs burden of CNS cancers was mainly due to YLLs, resulting from the complex nature of the disease. With the improvement of socioeconomic status, the incidence of CNS cancers increased in most provinces, although the DALYs remained roughly on par with low SDI regions. This was probably due to a general medical advancement in the country and polarization of medical care in high SDI regions. Although the high rate of YLDs indicates the great need for rehabilitation programs and economic consequences. Noticeable gender disparities were present for several variables, such as ASDR. Finally, although a significant portion of the CNS cancers mortality still comes from patients above 50, mortality has decreased in young patients aged under 15.

## AUTHOR CONTRIBUTIONS

**Mahdi Mahdavi:** Data curation (equal); formal analysis (equal); investigation (equal); writing – original draft (lead); writing – review and editing (equal). **Sahar Saeedi Moghaddam:** Data curation (equal); formal analysis (lead); investigation (equal); visualization (lead); writing – original draft (equal); writing – review and editing (equal). **Mohsen Abbasi‐Kangevari:** Investigation (equal); visualization (equal); writing – original draft (equal); writing – review and editing (supporting). **Esmaeil Mohammadi:** Investigation (supporting); writing – original draft (supporting). **Parnian Shobeiri:** Investigation (supporting); writing – original draft (supporting). **Guive Sharifi:** Investigation (supporting); writing – original draft (supporting). **Ali Jafari:** Investigation (supporting). **Negar Rezaei:** Conceptualization (equal); investigation (supporting); writing – original draft (supporting); writing – review and editing (supporting). **Narges Ebrahimi:** Investigation (supporting). **Nazila Rezaei:** Conceptualization (equal); investigation (supporting); writing – original draft (supporting); writing – review and editing (supporting). **Seyyed‐Hadi Ghamari:** Investigation (supporting); writing – original draft (supporting). **Mohammad‐Reza Malekpour:** Data curation (equal); formal analysis (supporting); visualization (supporting). **Majid Khalili:** Investigation (supporting); writing – original draft (supporting). **Bagher Larijani:** Conceptualization (equal); investigation (supporting). **Farzad Kompani:** Conceptualization (equal); investigation (supporting); supervision (lead); writing – original draft (supporting); writing – review and editing (supporting).

## CONFLICT OF INTEREST

The authors have no conflicts of interest to declare.

## ETHICS STATEMENT

The current study protocol was reviewed and approved by the Ethics Committee of the Endocrinology and Metabolism Research Institute of Tehran University of Medical Sciences (Ethics code: IR.TUMS.EMRI.REC.1400.029).

## INFORMED CONSENT STATEMENT

The data used in this study were obtained from the GBD study. Thus, obtaining a consent form was not applicable.

## Supporting information


Figure S1.

Figure S2.

Figure S3.

Figure S4.

Figure S5.

Figure S6.

Table S1.

Table S2.

Table S3.

## Data Availability

The supporting data are available from the Non‐communicable Diseases Research Center, Endocrinology and Metabolism Population Sciences Institute, Tehran University of Medical Sciences, Tehran, Iran. However, restrictions apply to the availability of the data, which were used under license for the current study, and so are not publicly available. Authors can provide the data upon reasonable request and with permission of the Institute for Health Metrics and Evaluation.
