# Supplementary figures and images for: National and subnational burden of brain and central nervous system cancers in Iran, 1990–2019: Results from the global burden of disease study 2019
Source: Cancer Med. 2023 Jan 9;12(7):8614–28. doi: 10.1002/cam4.5553 (PMC10134290; doi:10.1002/cam4.5553)

Supplementary Figure 1

## Incidence

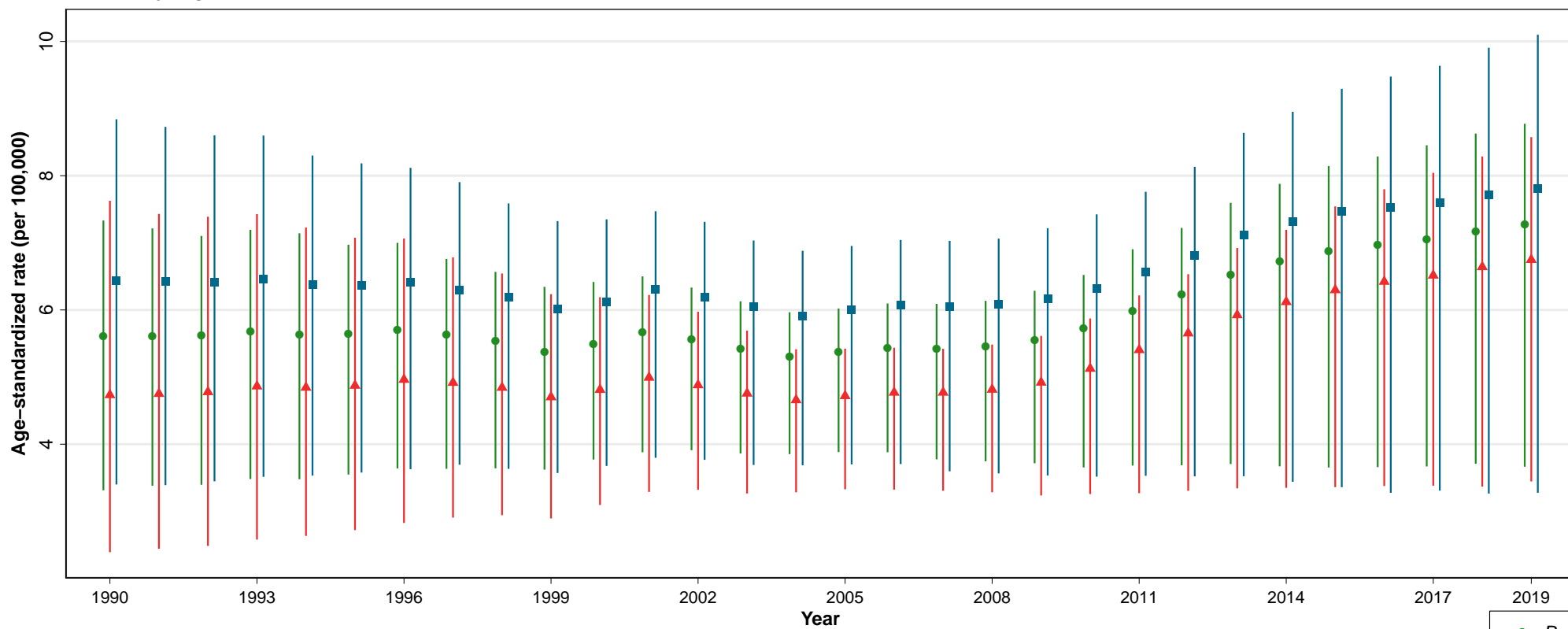

## Prevalence

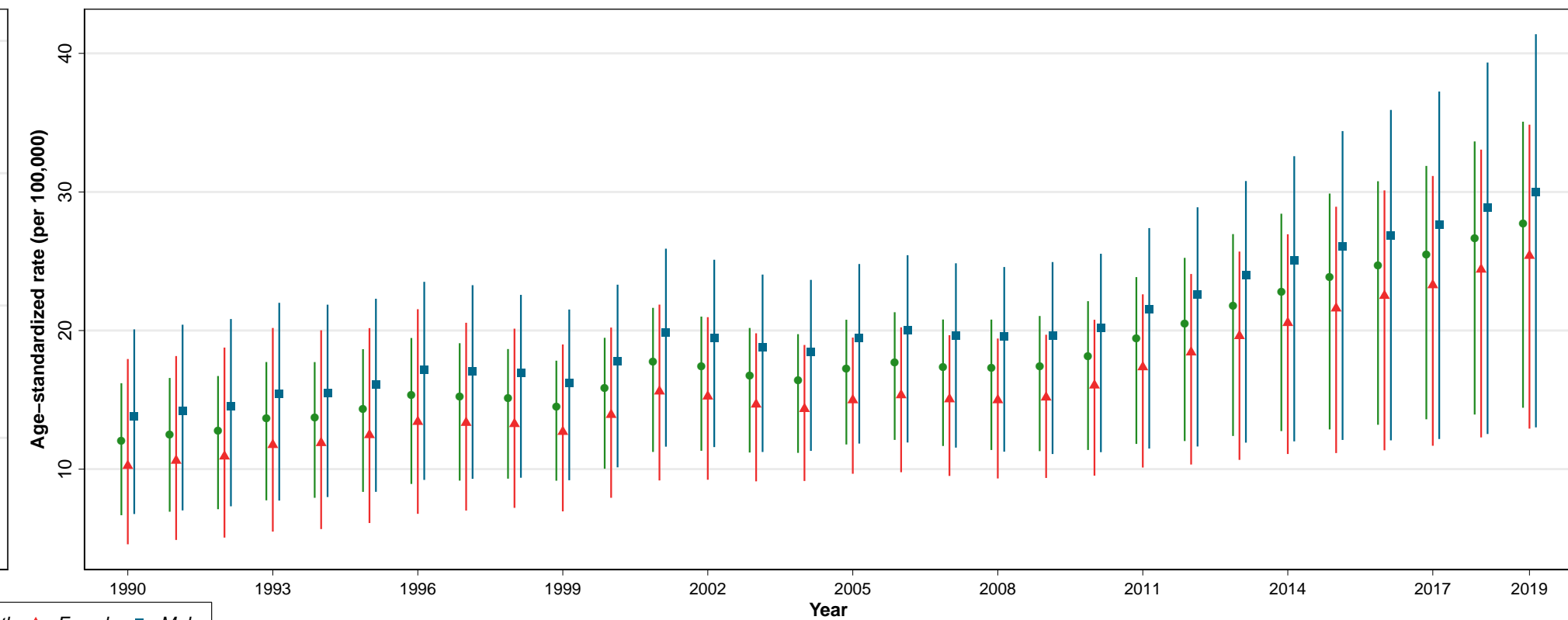

## Deaths

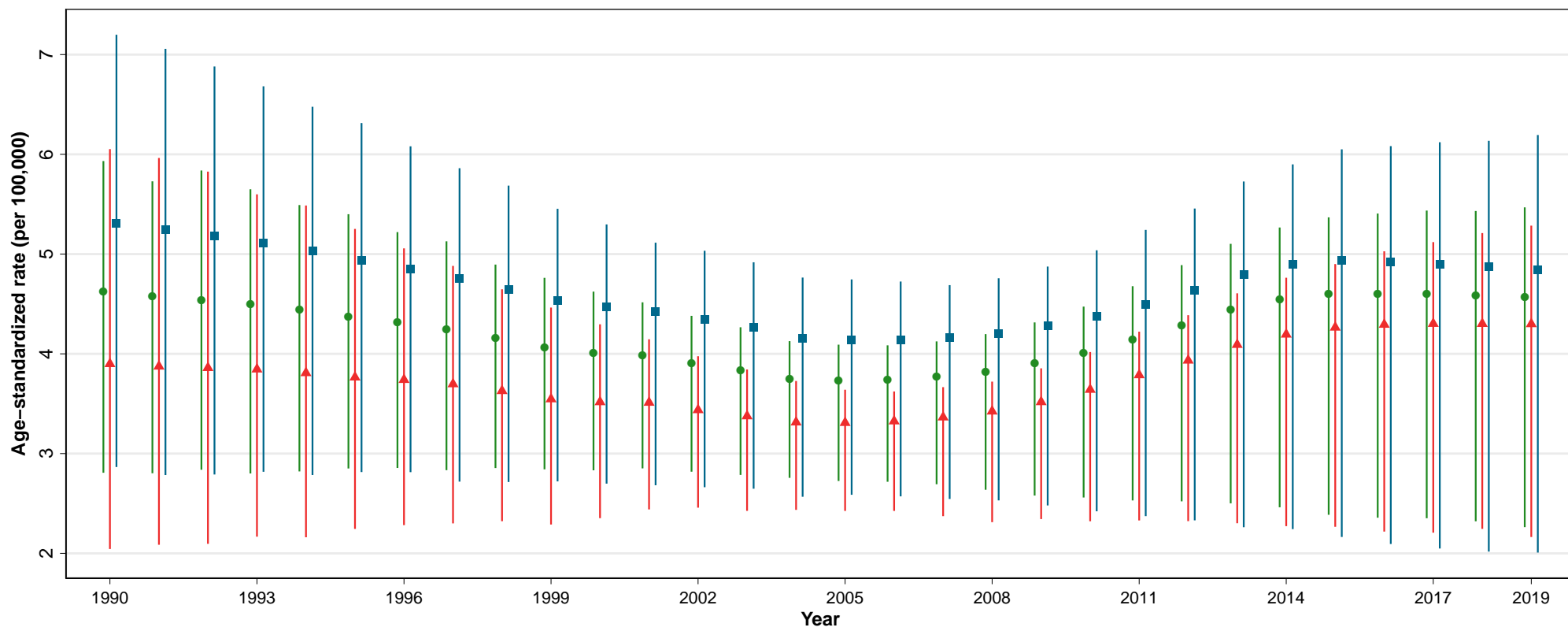

## DALYs

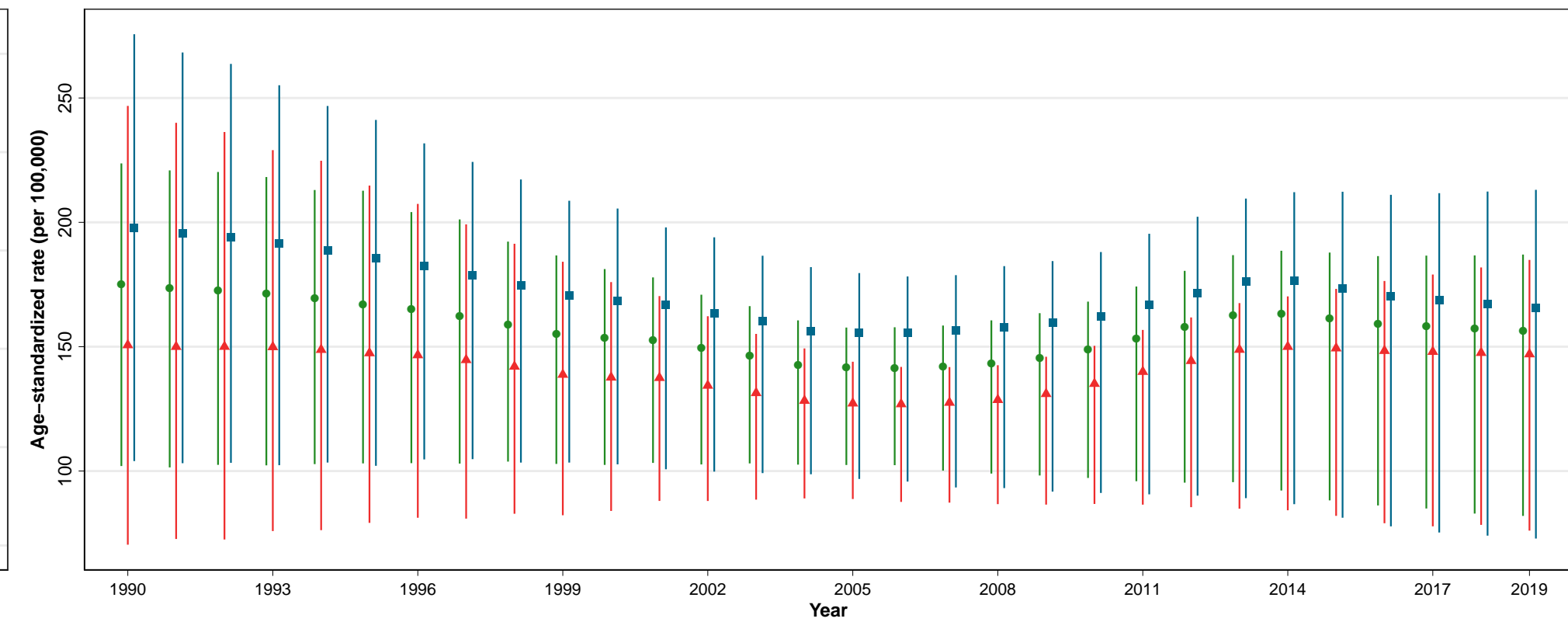

Supplement: Supplementary file 1 — Figure S1. [file CAM4-12-8614-s002.pdf]

Supplementary Figure 2

Incidence

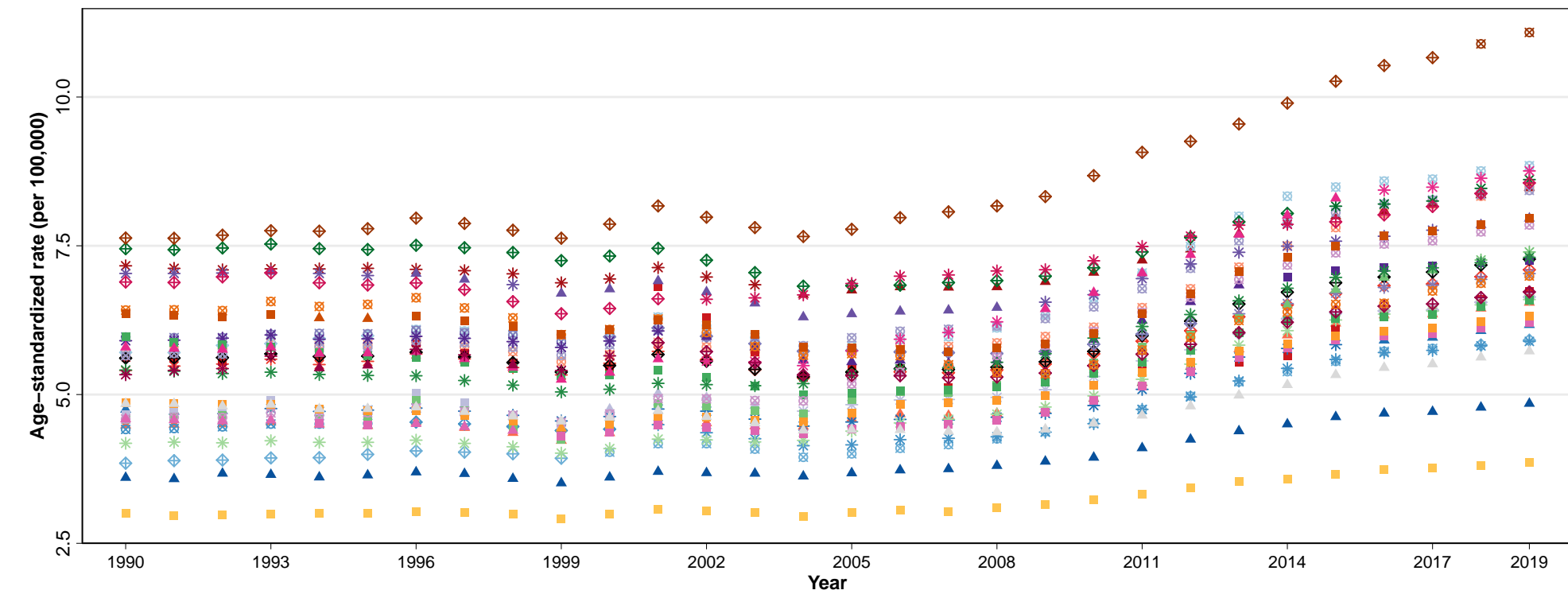

Prevalence

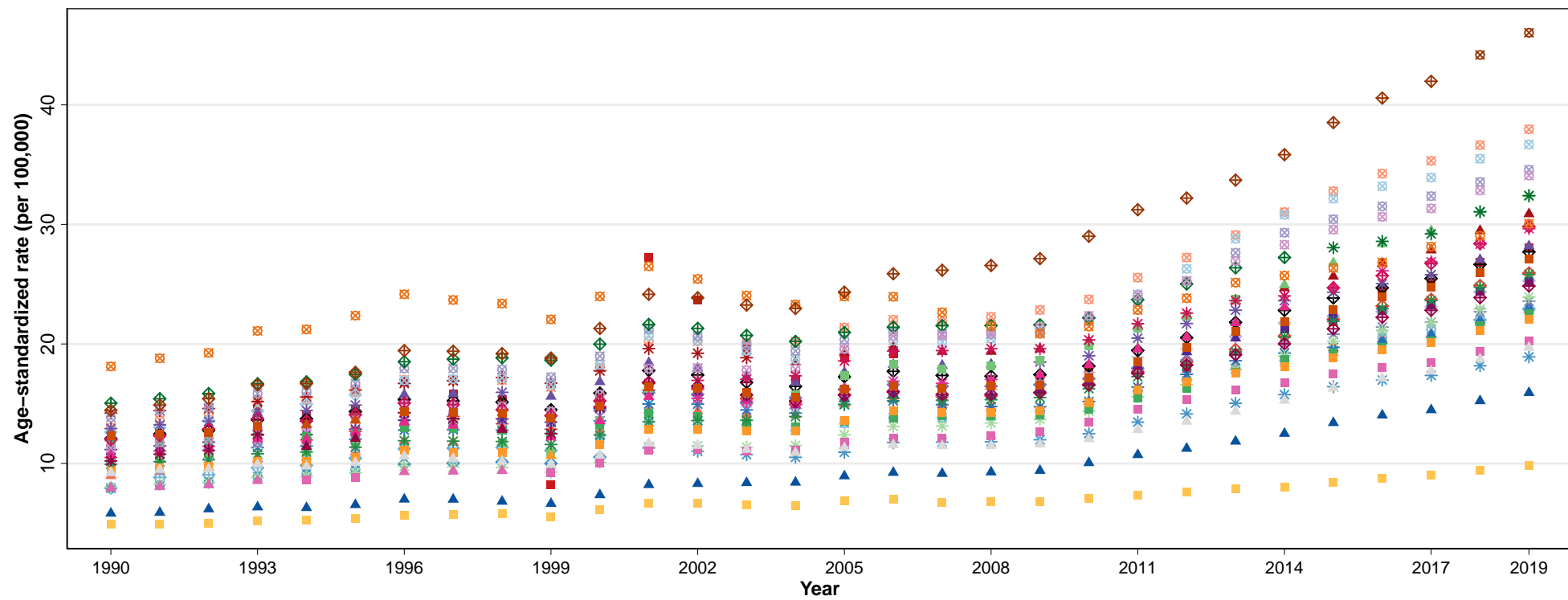

Deaths

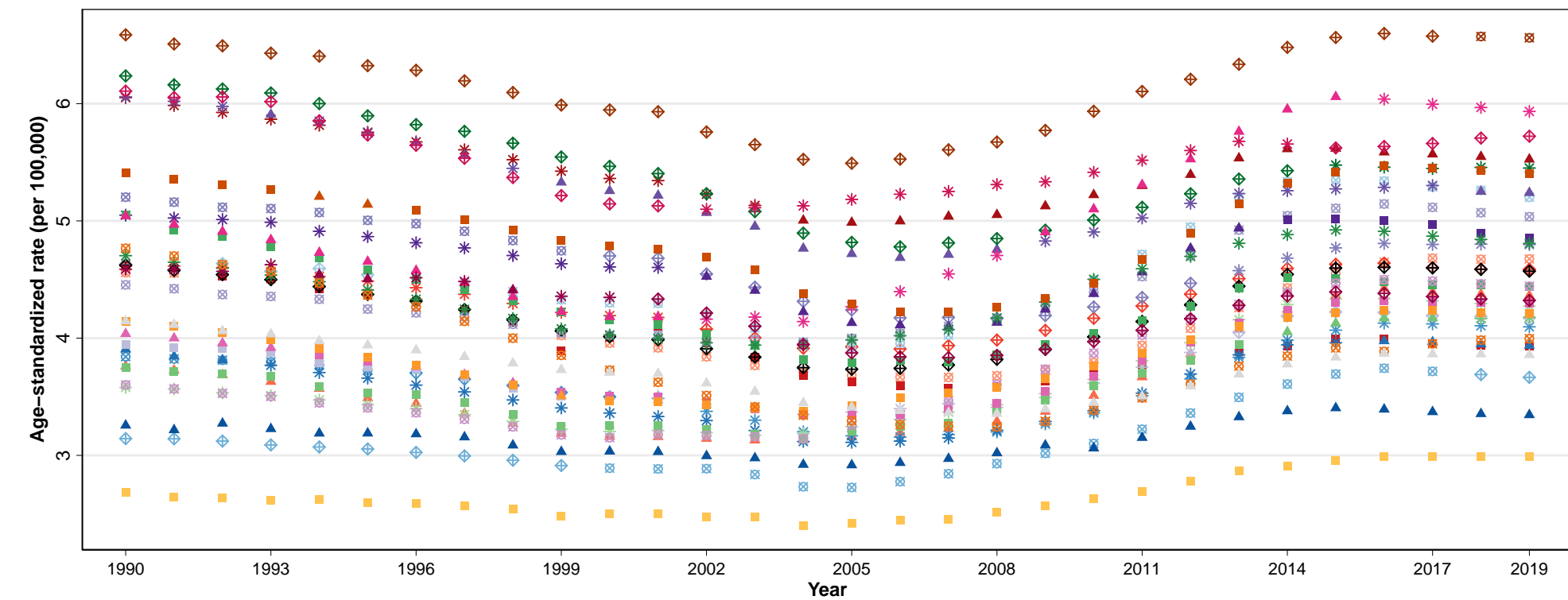

DALYs

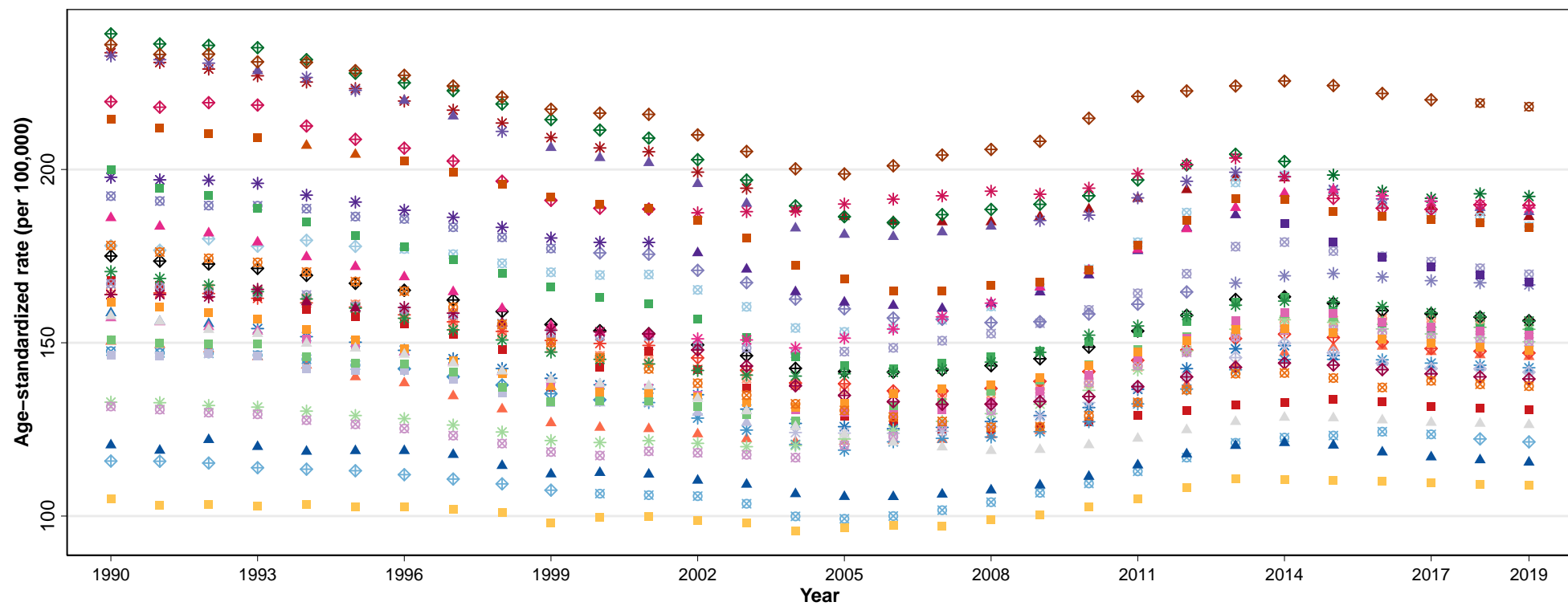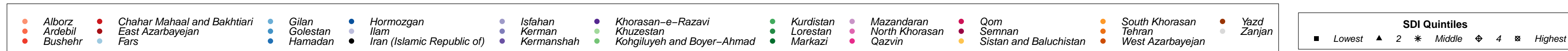

Supplement: Supplementary file 2 — Figure S2. [file CAM4-12-8614-s003.pdf]

Incidence

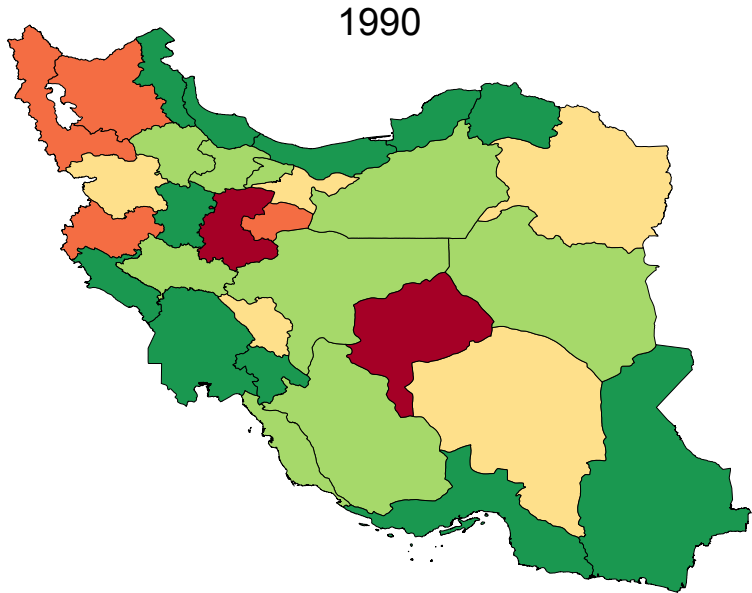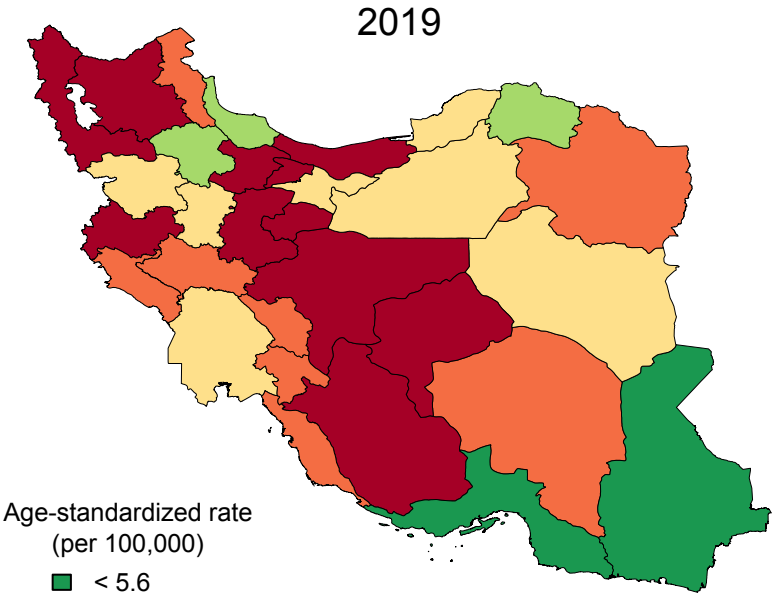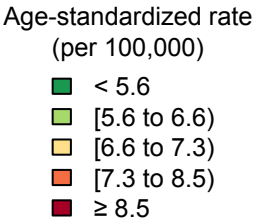

Prevalence

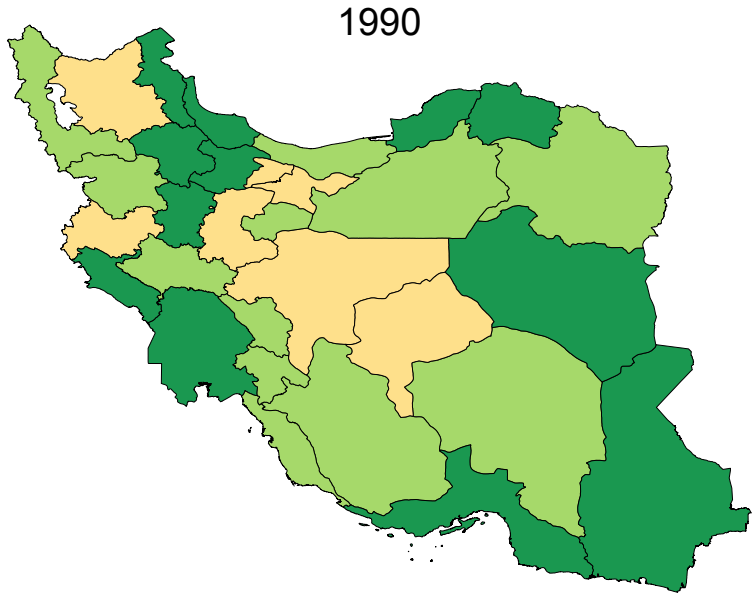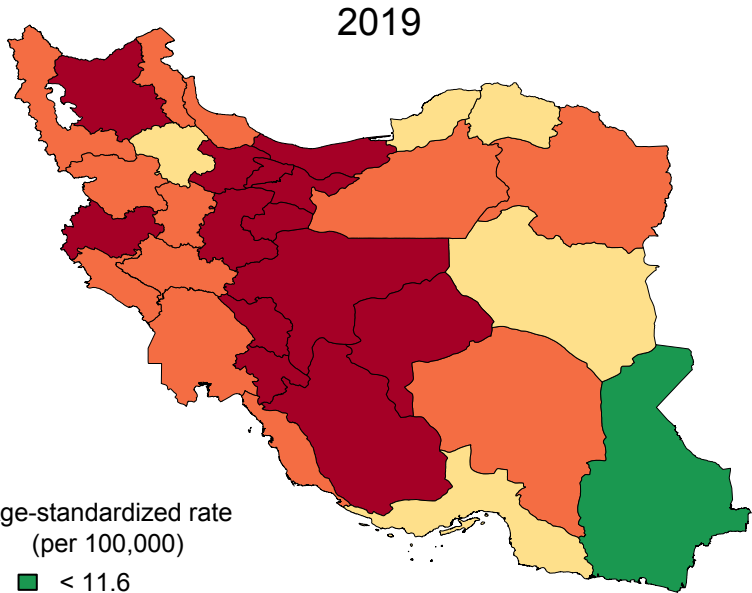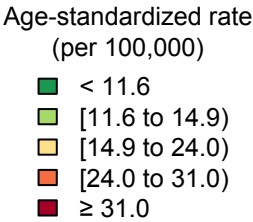

Deaths

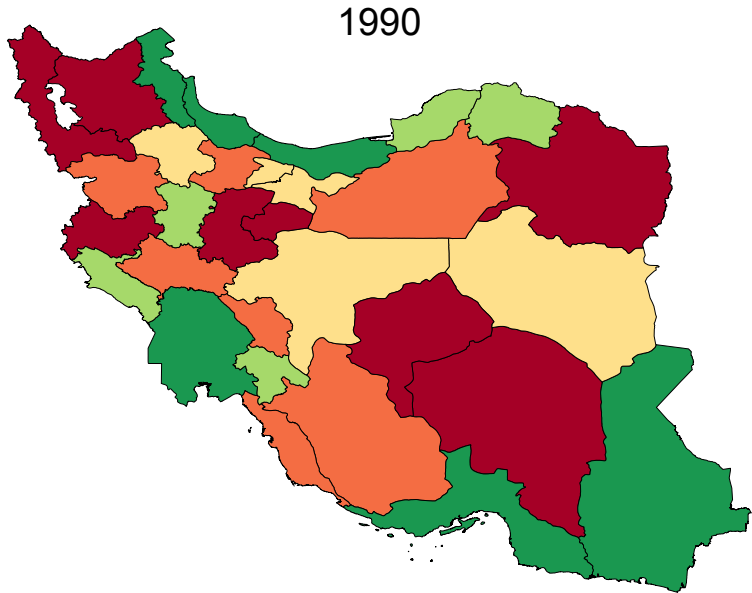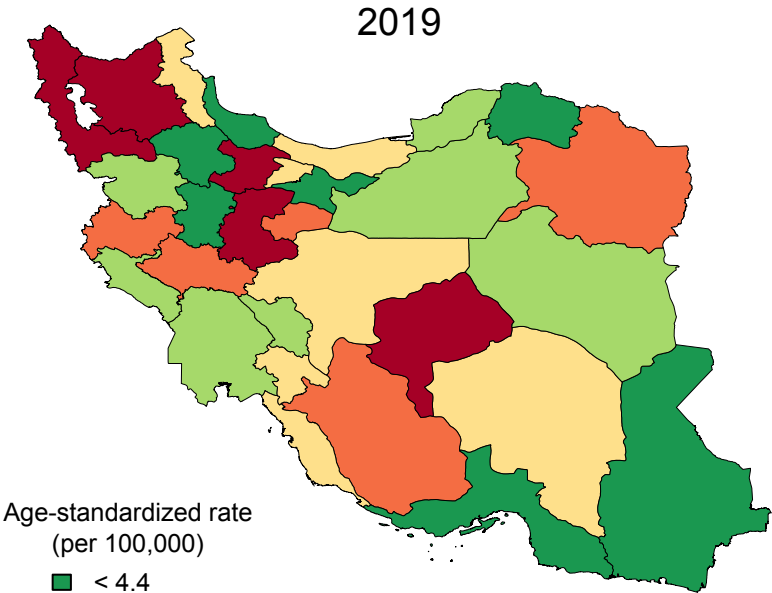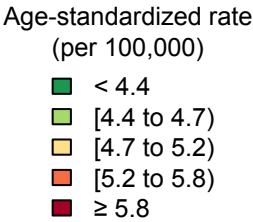

DALYs

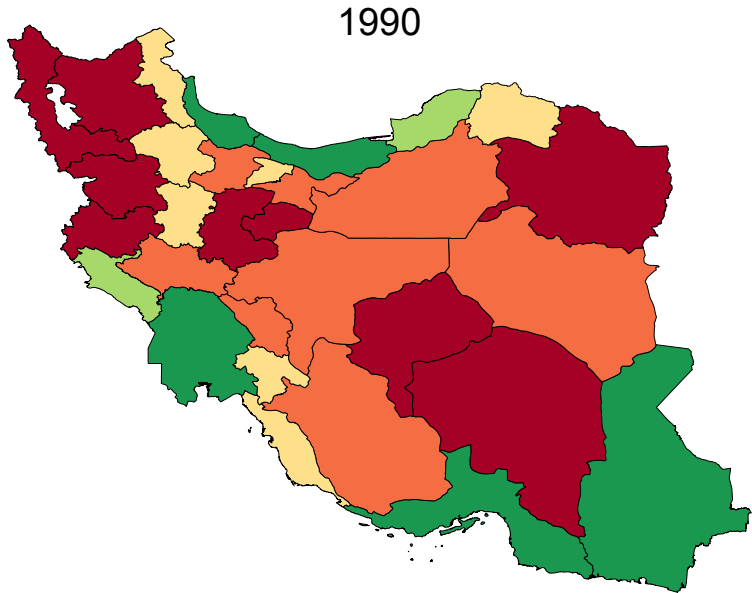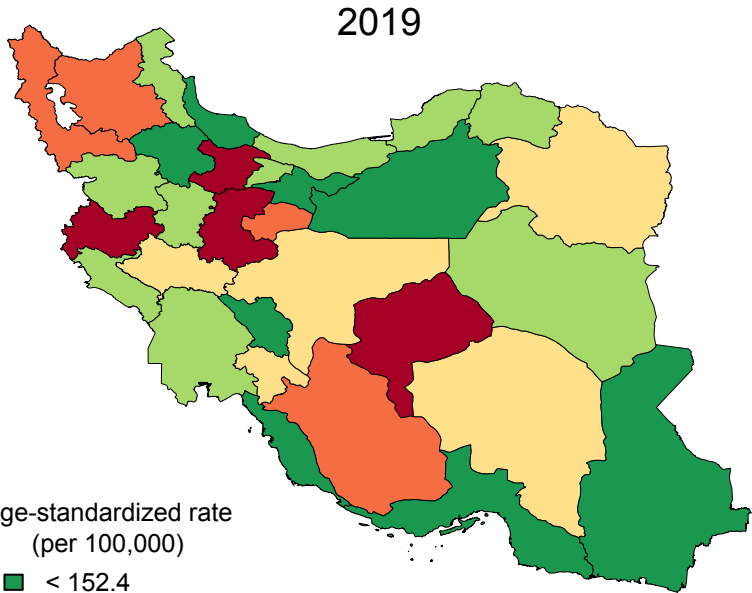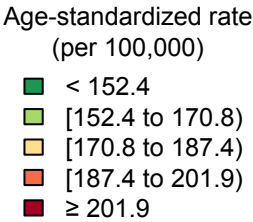

Supplement: Supplementary file 3 — Figure S3. [file CAM4-12-8614-s006.zip › CAM4_5553_Supplementary Figure 3B.pdf]

**Both**

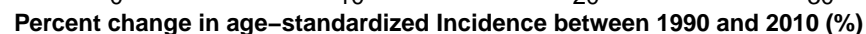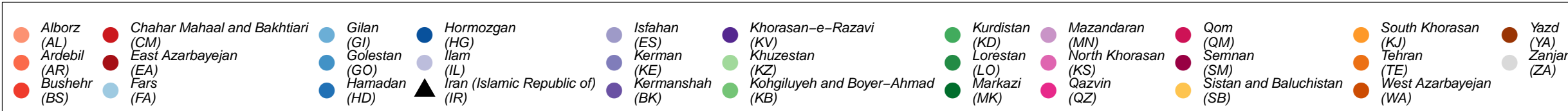

Supplement: Supplementary file 4 — Figure S4. [file CAM4-12-8614-s001.zip › CAM4_5553_Supplementary Figure 4A.pdf]

Supplementary Figure 4B

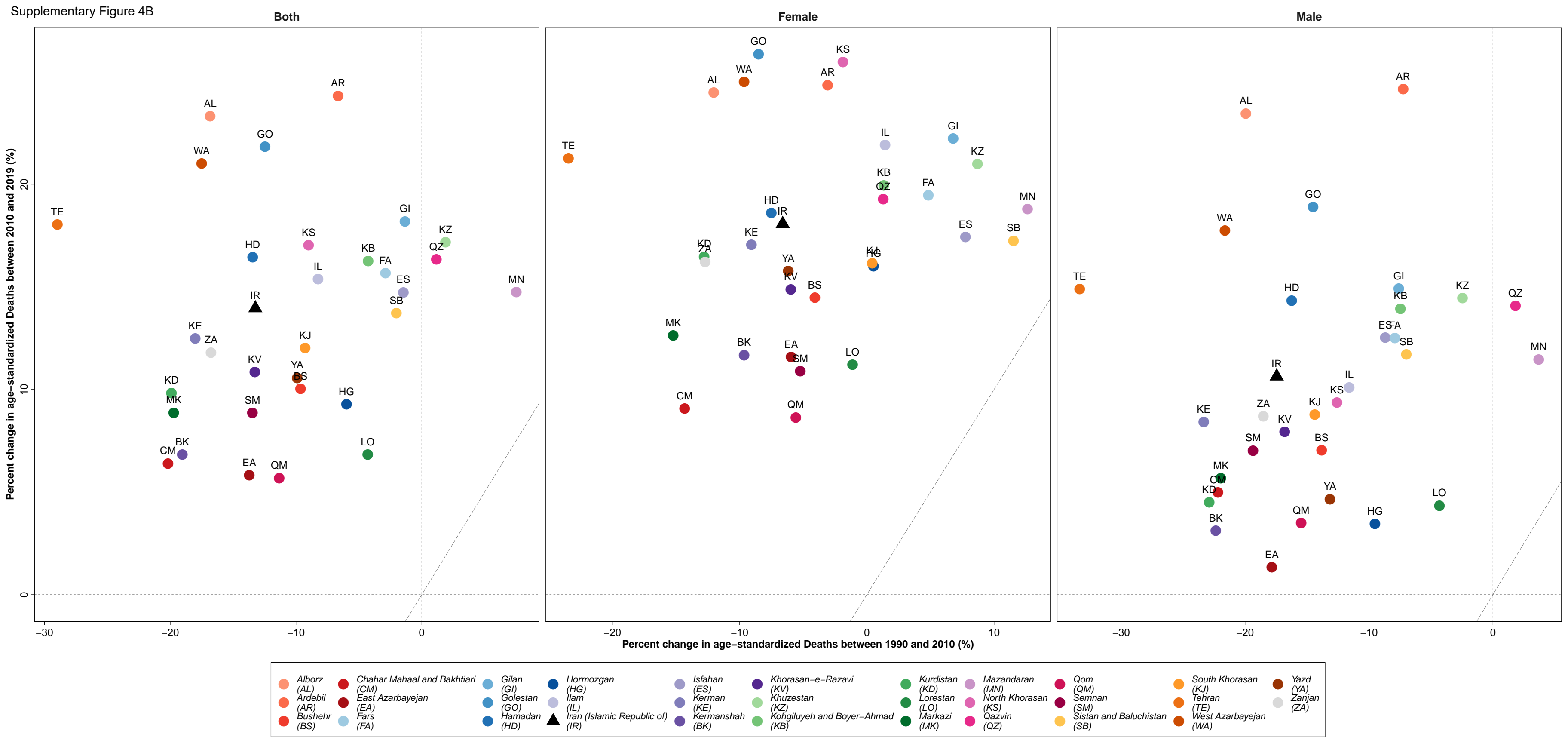

Supplement: Supplementary file 4 — Figure S4. [file CAM4-12-8614-s001.zip › CAM4_5553_Supplementary Figure 4B.pdf]

Supplementary Figure 6

Both

Female

Male

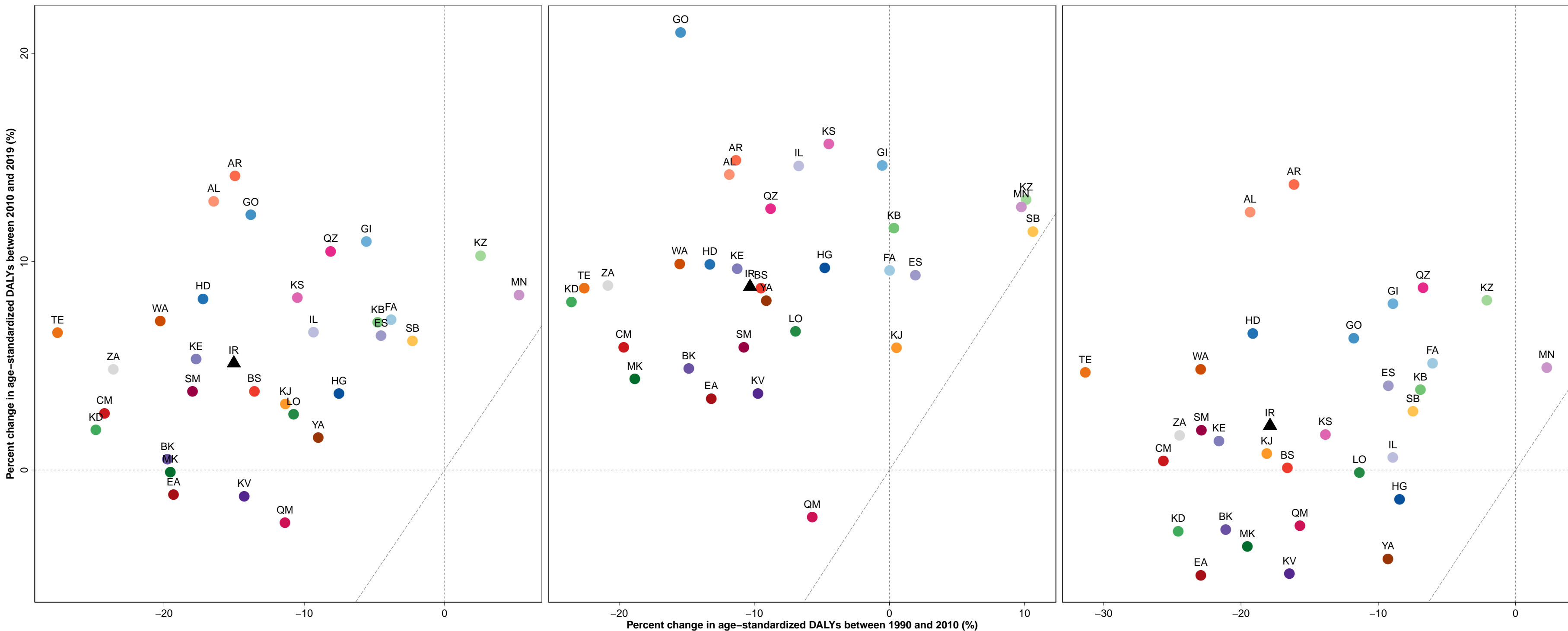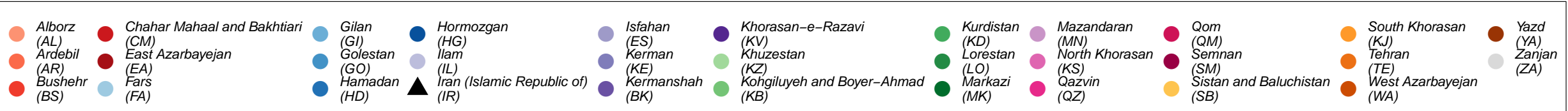

Supplement: Supplementary file 6 — Figure S6. [file CAM4-12-8614-s007.pdf]
