# Supplementary material for: National and subnational burden of brain and central nervous system cancers in Iran, 1990–2019: Results from the global burden of disease study 2019
Source: Cancer Med. 2023 Jan 9;12(7):8614–28. doi: 10.1002/cam4.5553 (PMC10134290; doi:10.1002/cam4.5553)

Supplementary Figure 3A

Incidence

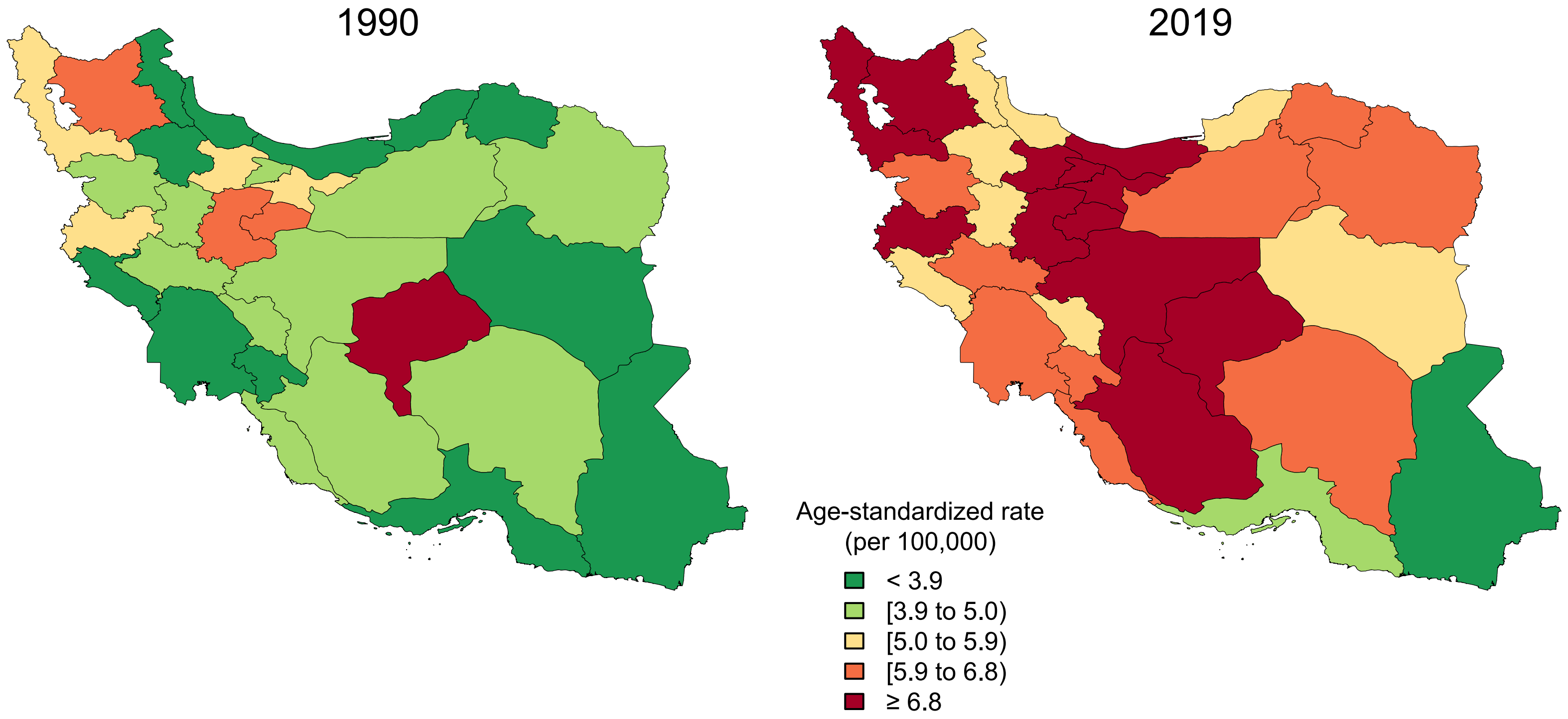

Prevalence

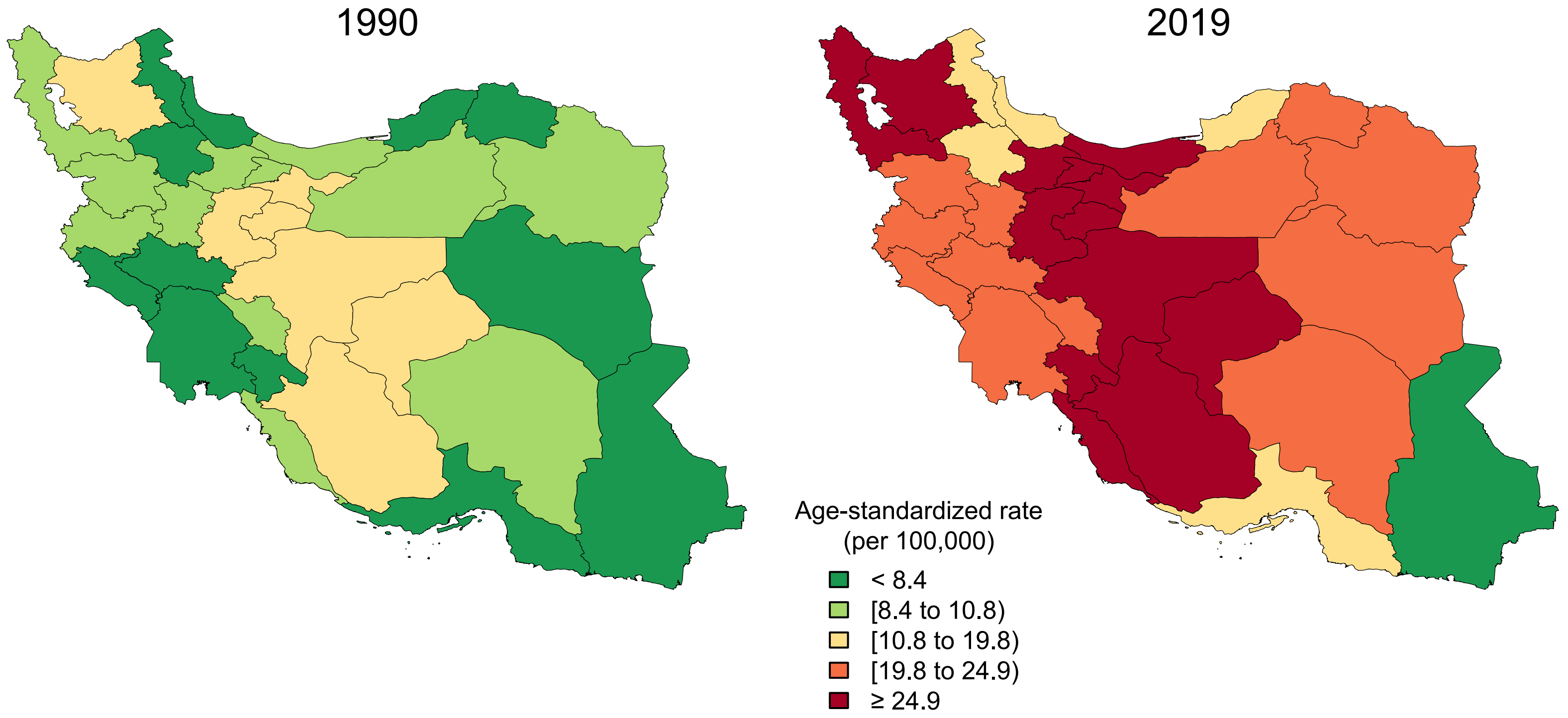

Deaths

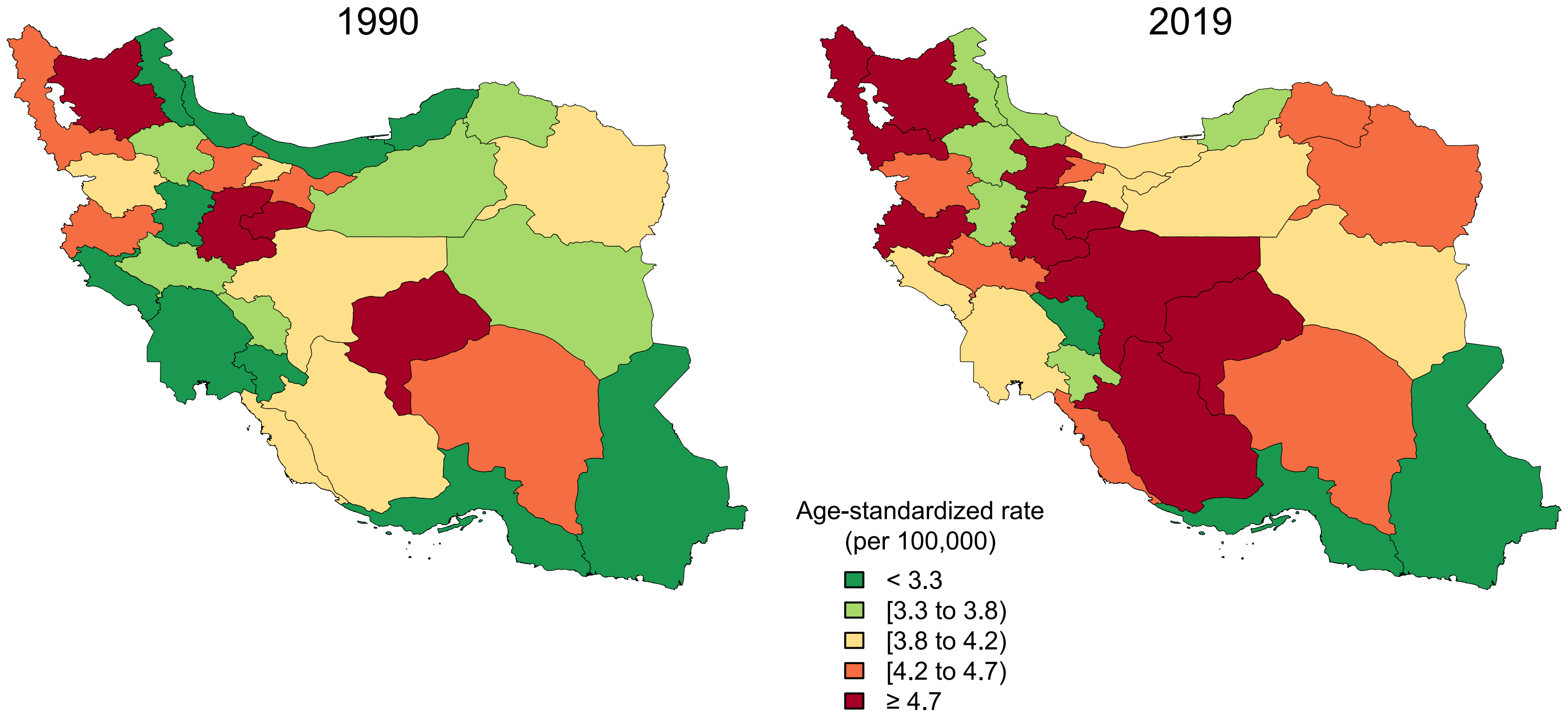

DALYs

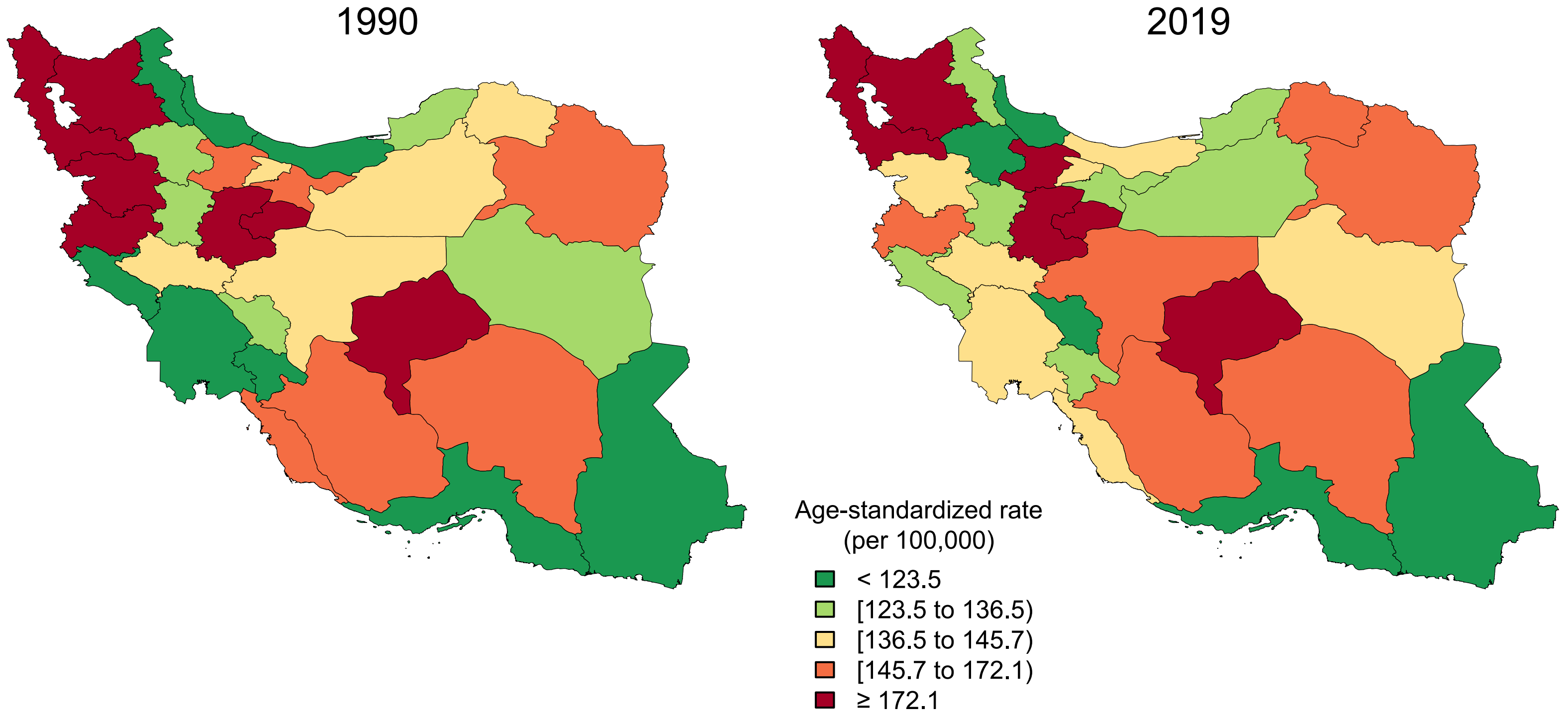

Supplement: Supplementary file 3 — Figure S3. [file CAM4-12-8614-s006.zip › CAM4_5553_Supplementary Figure 3A.pdf]
