# Supplementary material for: National and subnational burden of brain and central nervous system cancers in Iran, 1990–2019: Results from the global burden of disease study 2019
Source: Cancer Med. 2023 Jan 9;12(7):8614–28. doi: 10.1002/cam4.5553 (PMC10134290; doi:10.1002/cam4.5553)
Supplement: Supplementary file 5 — Figure S5. [file CAM4-12-8614-s005.pdf]

Supplementary Figure 5

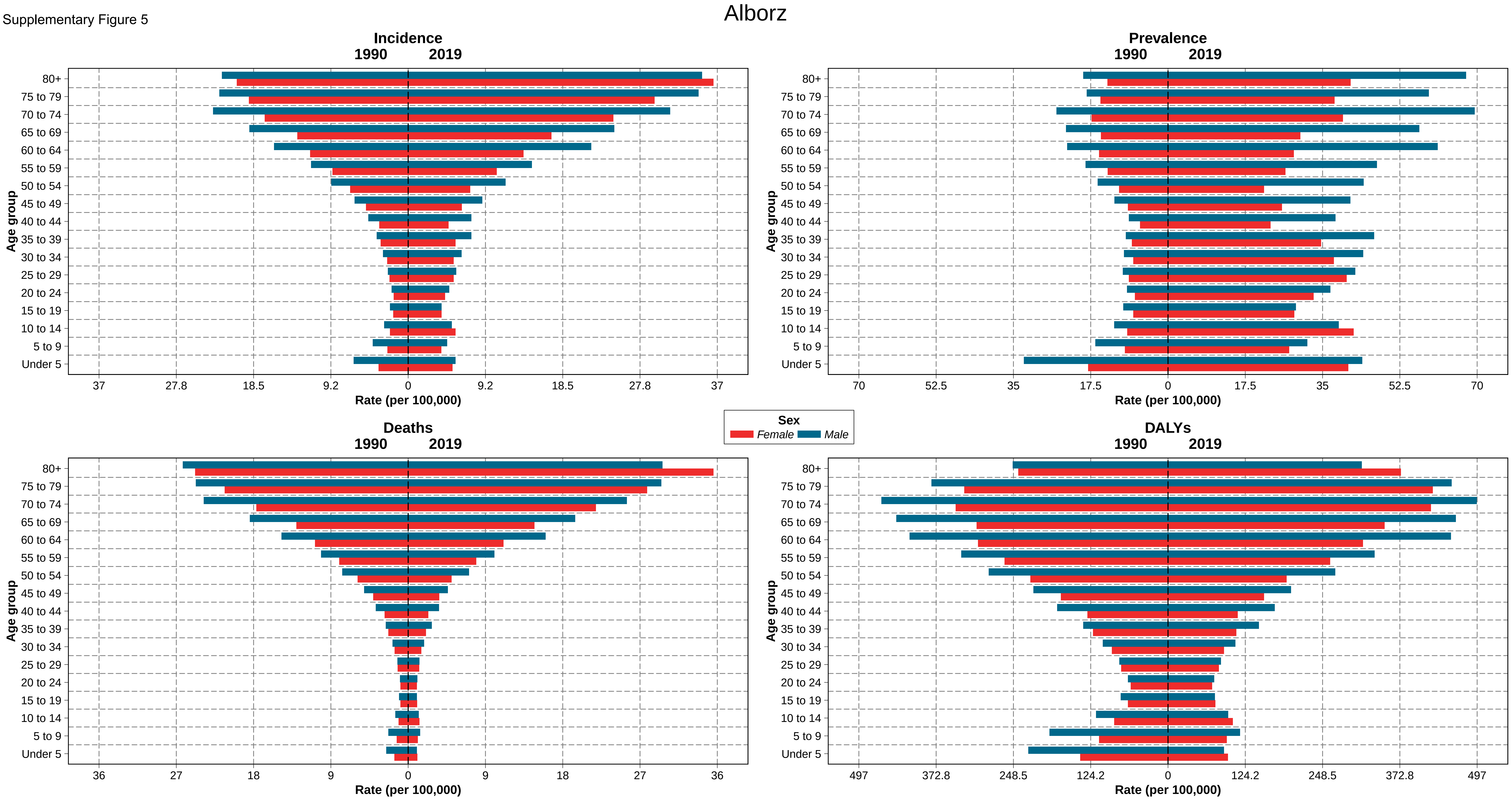

Ardebil

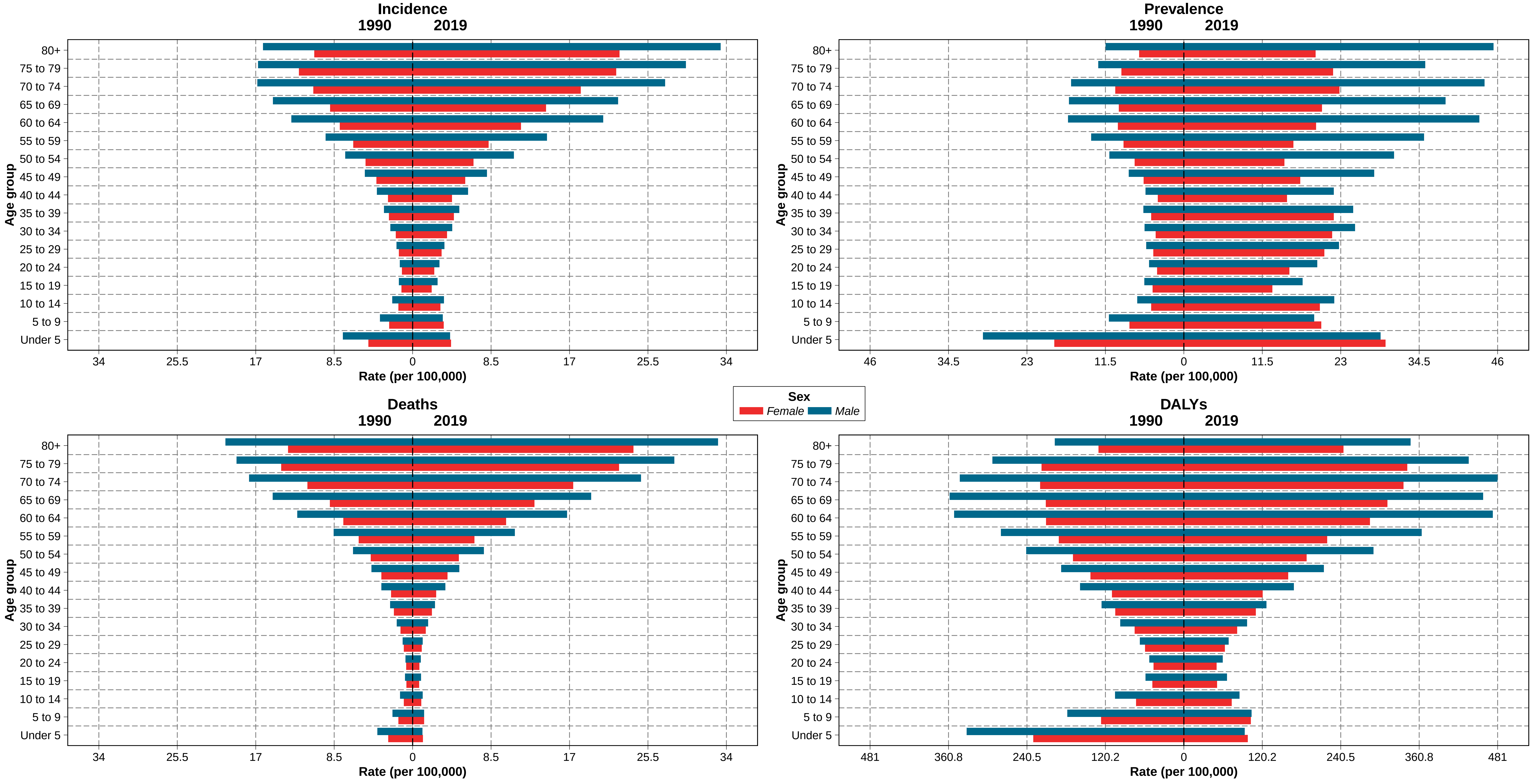

Incidence
1990
2019
Prevalence
1990
2019
Deaths
1990
2019
DALYs
1990
2019
Age group
80+
75 to 79
70 to 74
65 to 69
60 to 64
55 to 59
50 to 54
45 to 49
40 to 44
35 to 39
30 to 34
25 to 29
20 to 24
15 to 19
10 to 14
5 to 9
Under 5
34 25.5 17 8.5 0 8.5 17 25.5 34
46 34.5 23 11.5 0 11.5 23 34.5 46
481 360.8 240.5 120.2 0 120.2 240.5 360.8 481
Rate (per 100,000)
Sex
Female
Male

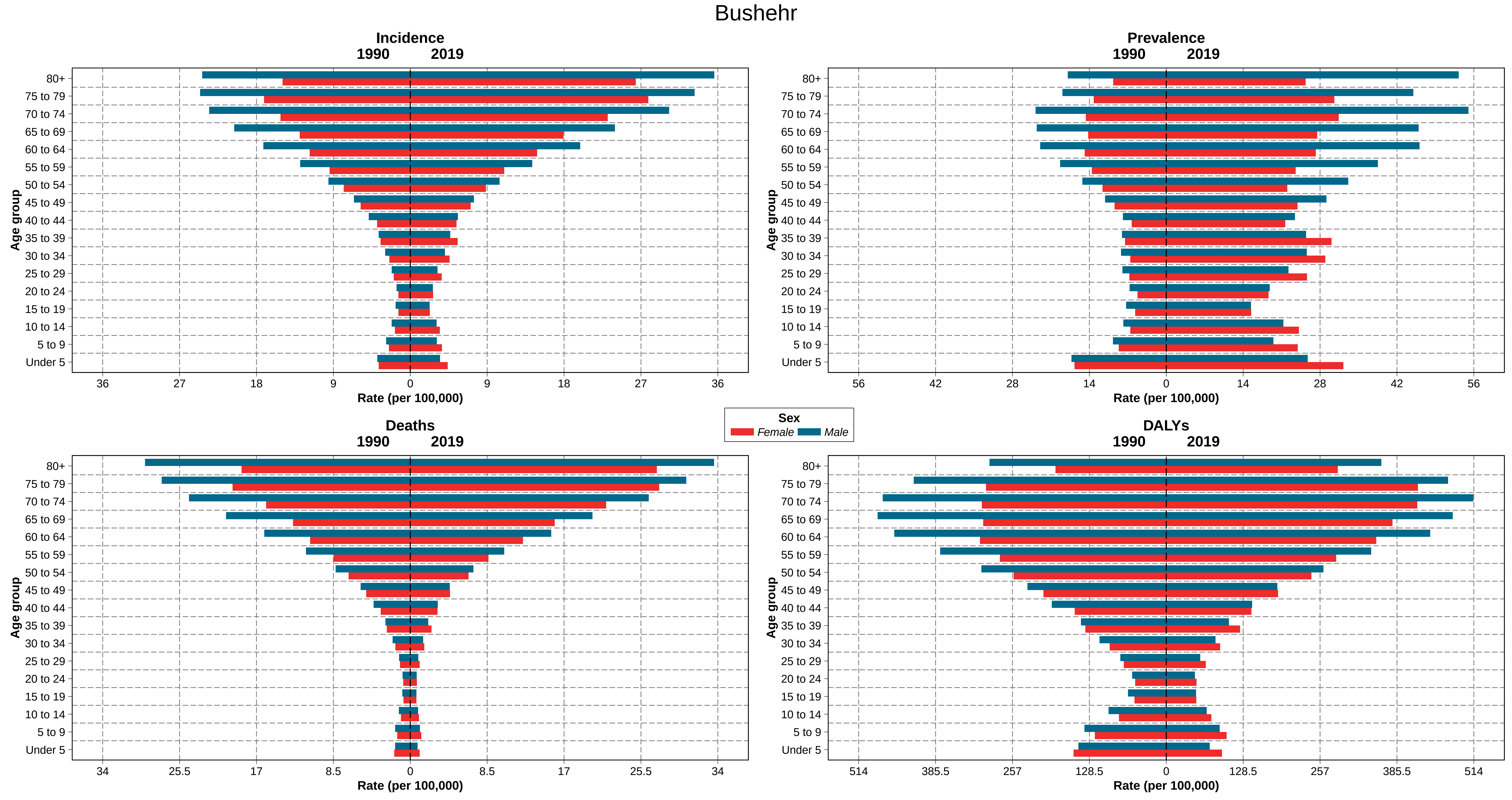

Bushehr
Incidence
1990
2019
Prevalence
1990
2019
Deaths
1990
2019
DALYs
1990
2019
Age group
80+
75 to 79
70 to 74
65 to 69
60 to 64
55 to 59
50 to 54
45 to 49
40 to 44
35 to 39
30 to 34
25 to 29
20 to 24
15 to 19
10 to 14
5 to 9
Under 5
36 27 18 9 0 9 18 27 36
56 42 28 14 0 14 28 42 56
34 25.5 17 8.5 0 8.5 17 25.5 34
514 385.5 257 128.5 0 128.5 257 385.5 514
Rate (per 100,000)
Sex
Female
Male

Chahar Mahaal and Bakhtiari

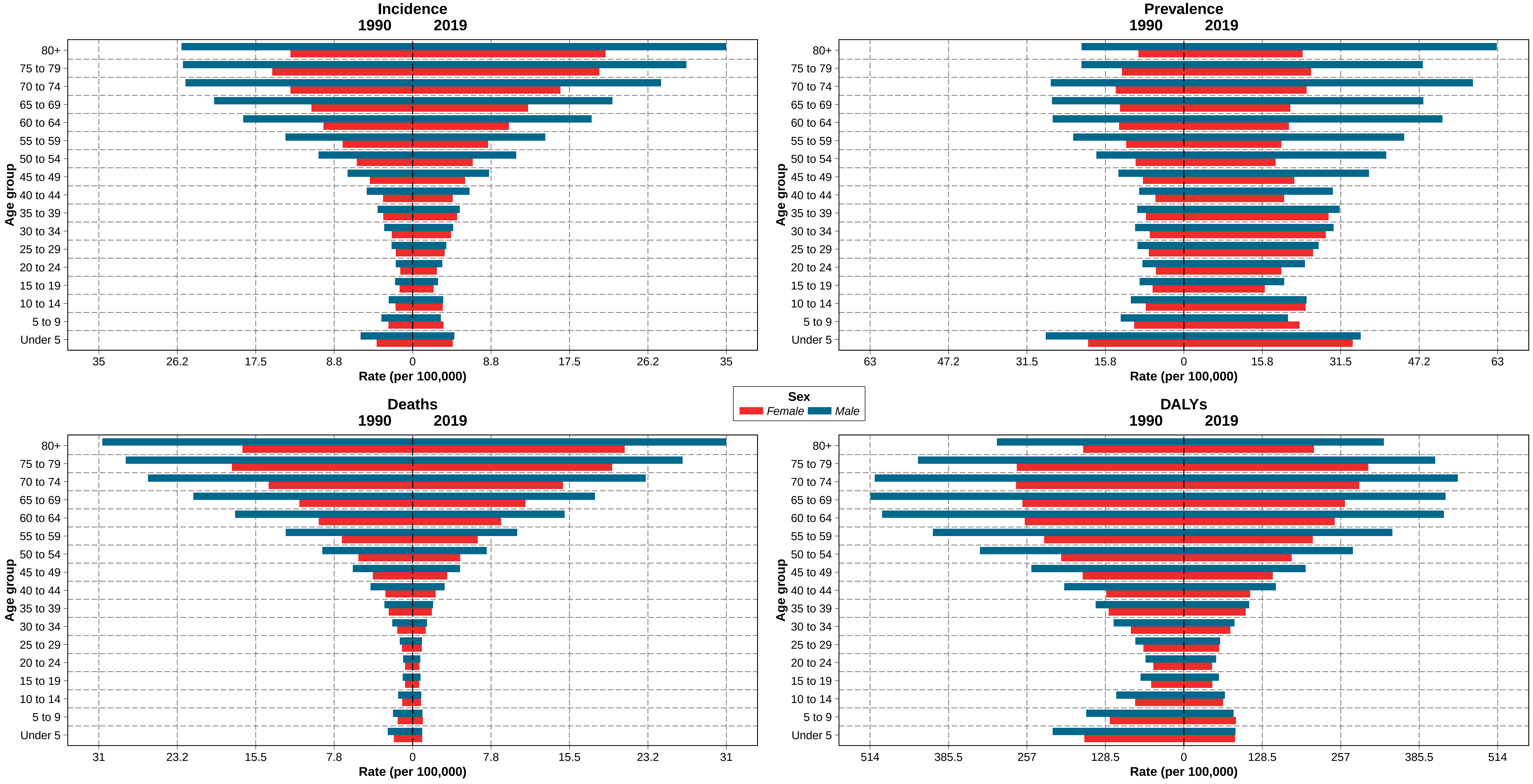

East Azarbayejan

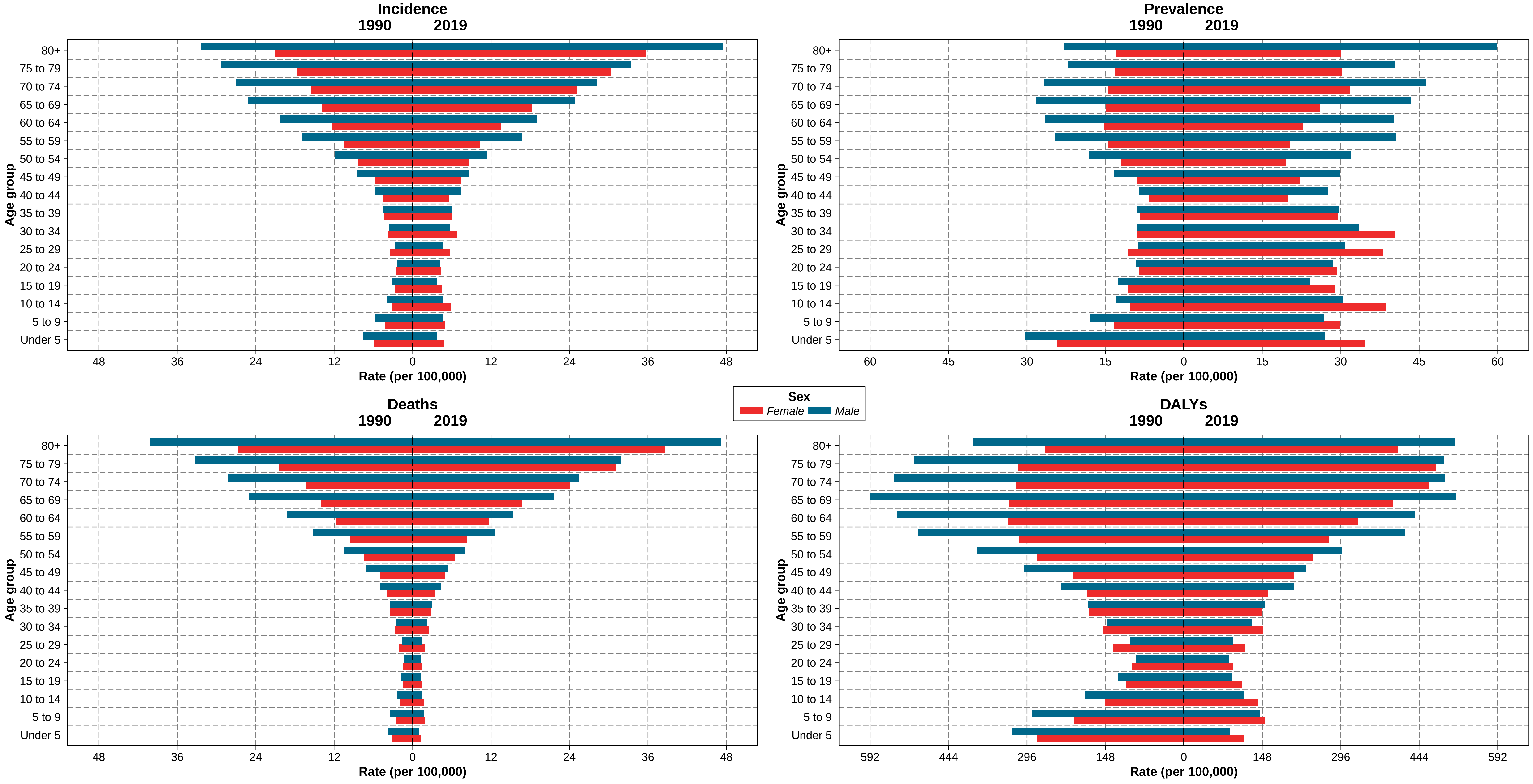

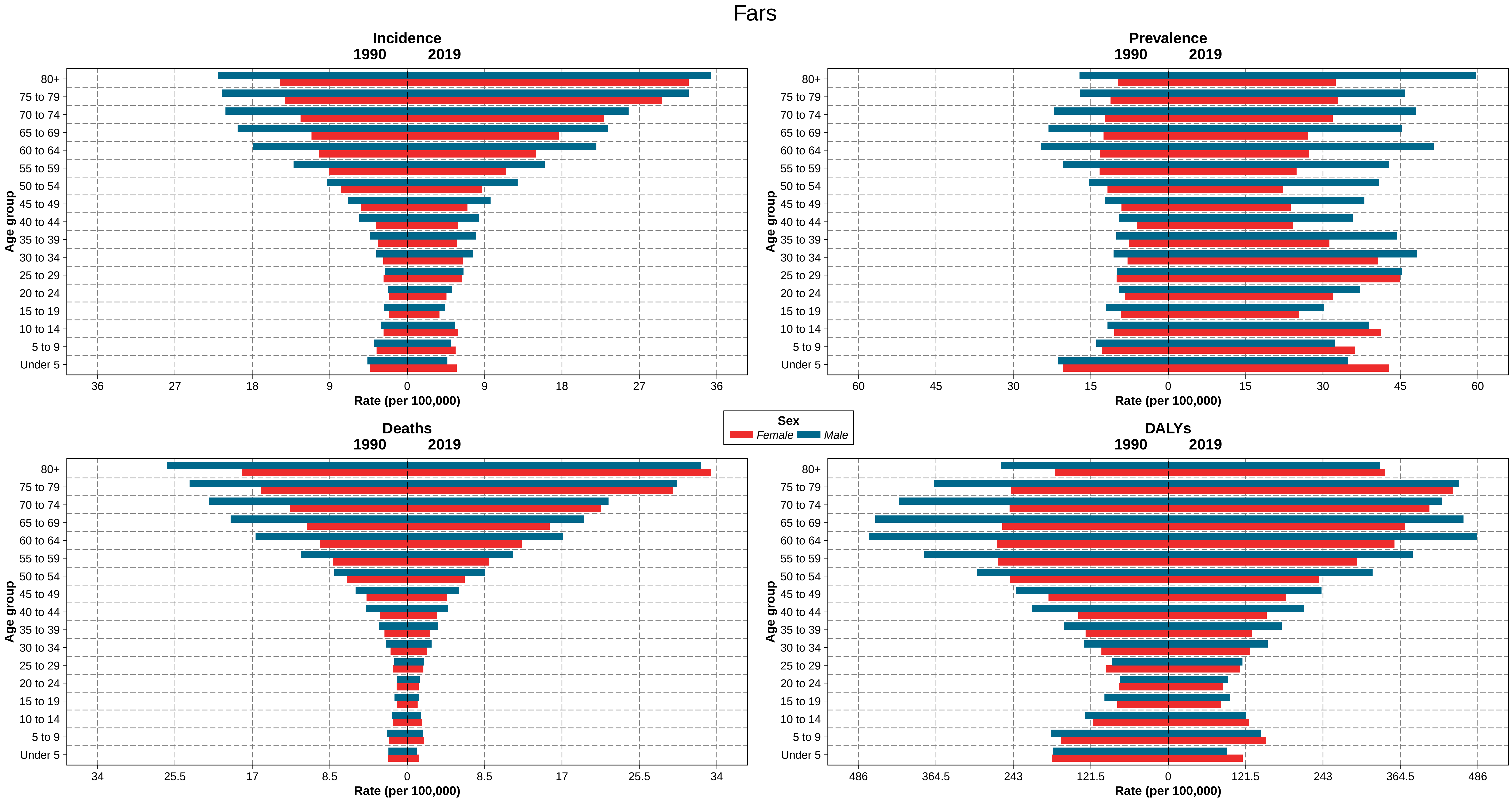
Fars
Incidence
1990
2019
Prevalence
1990
2019
Deaths
1990
2019
DALYs
1990
2019
Age group
80+
75 to 79
70 to 74
65 to 69
60 to 64
55 to 59
50 to 54
45 to 49
40 to 44
35 to 39
30 to 34
25 to 29
20 to 24
15 to 19
10 to 14
5 to 9
Under 5
36 27 18 9 0 9 18 27 36
60 45 30 15 0 15 30 45 60
34 25.5 17 8.5 0 8.5 17 25.5 34
486 364.5 243 121.5 0 121.5 243 364.5 486
Rate (per 100,000)
Sex
Female
Male

Gilan

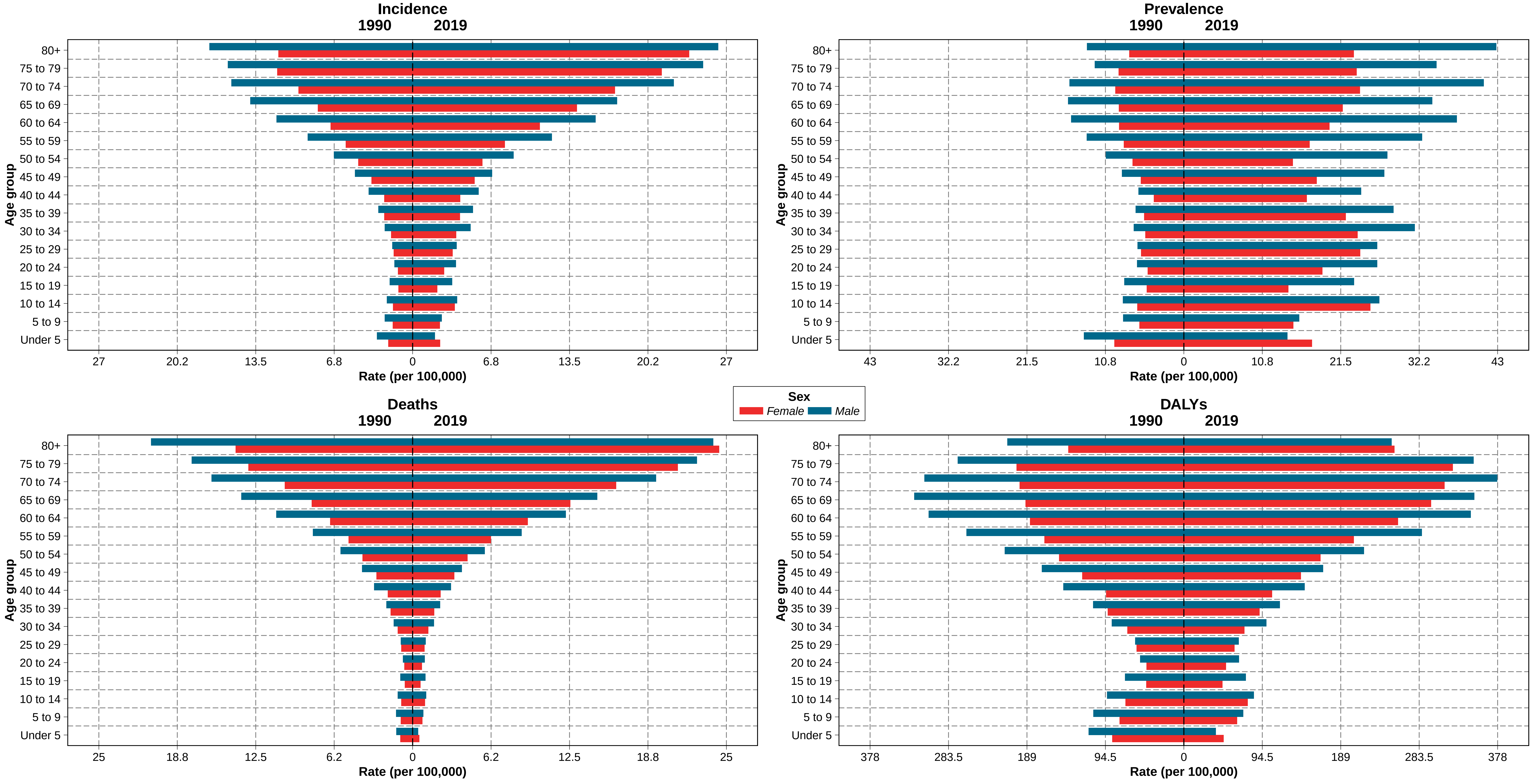

Incidence
1990
2019
Prevalence
Deaths
DALYs
Age group
80+
75 to 79
70 to 74
65 to 69
60 to 64
55 to 59
50 to 54
45 to 49
40 to 44
35 to 39
30 to 34
25 to 29
20 to 24
15 to 19
10 to 14
5 to 9
Under 5
Rate (per 100,000)
27 20.2 13.5 6.8 0 6.8 13.5 20.2 27
43 32.2 21.5 10.8 0 10.8 21.5 32.2 43
25 18.8 12.5 6.2 0 6.2 12.5 18.8 25
378 283.5 189 94.5 0 94.5 189 283.5 378
Sex
Female
Male

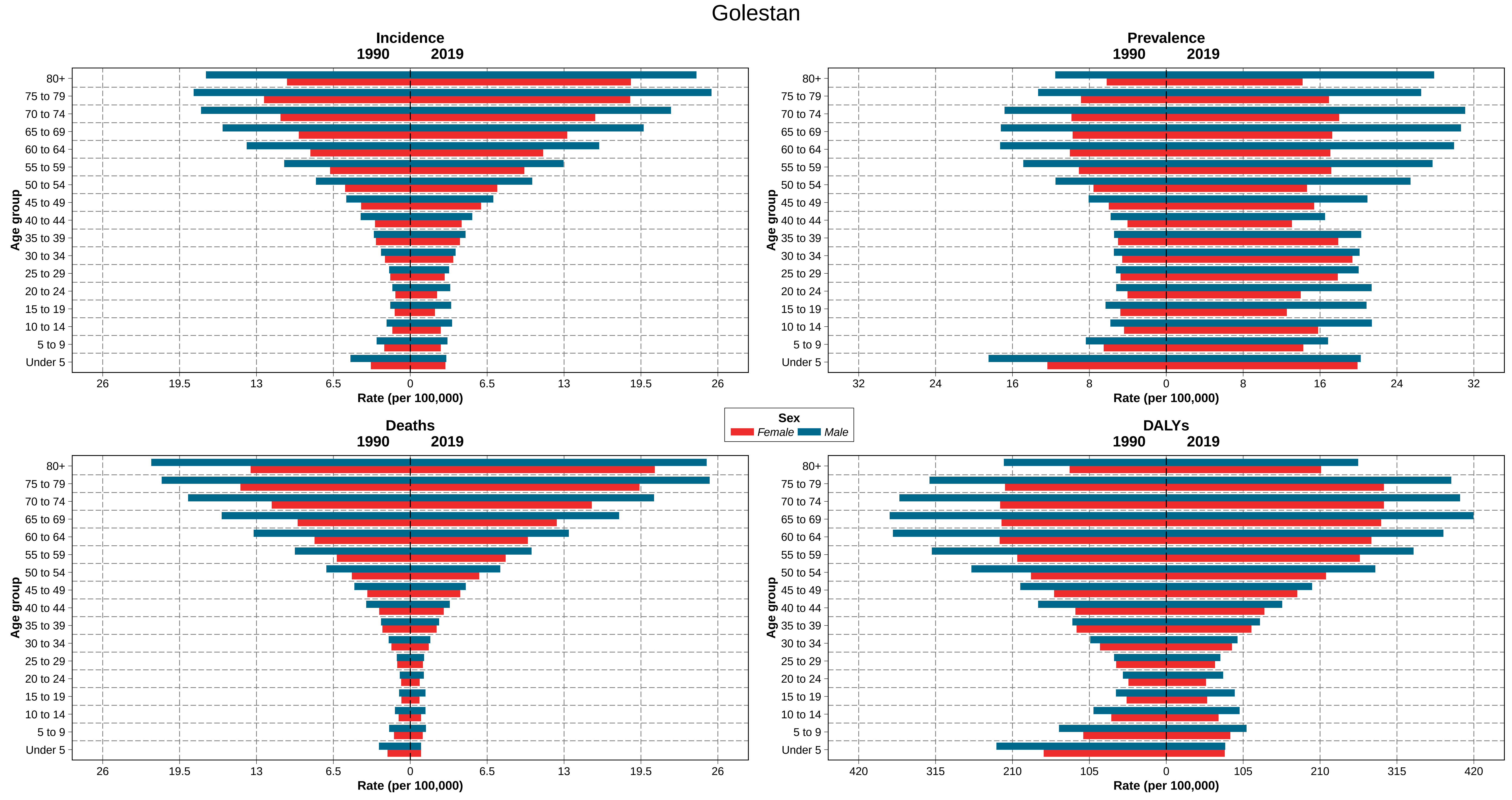
Golestan
Incidence
Prevalence
Deaths
DALYs
1990
2019
Age group
80+
75 to 79
70 to 74
65 to 69
60 to 64
55 to 59
50 to 54
45 to 49
40 to 44
35 to 39
30 to 34
25 to 29
20 to 24
15 to 19
10 to 14
5 to 9
Under 5
26
19.5
13
6.5
0
32
24
16
8
420
315
210
105
Rate (per 100,000)
Sex
Female
Male

Hamadan

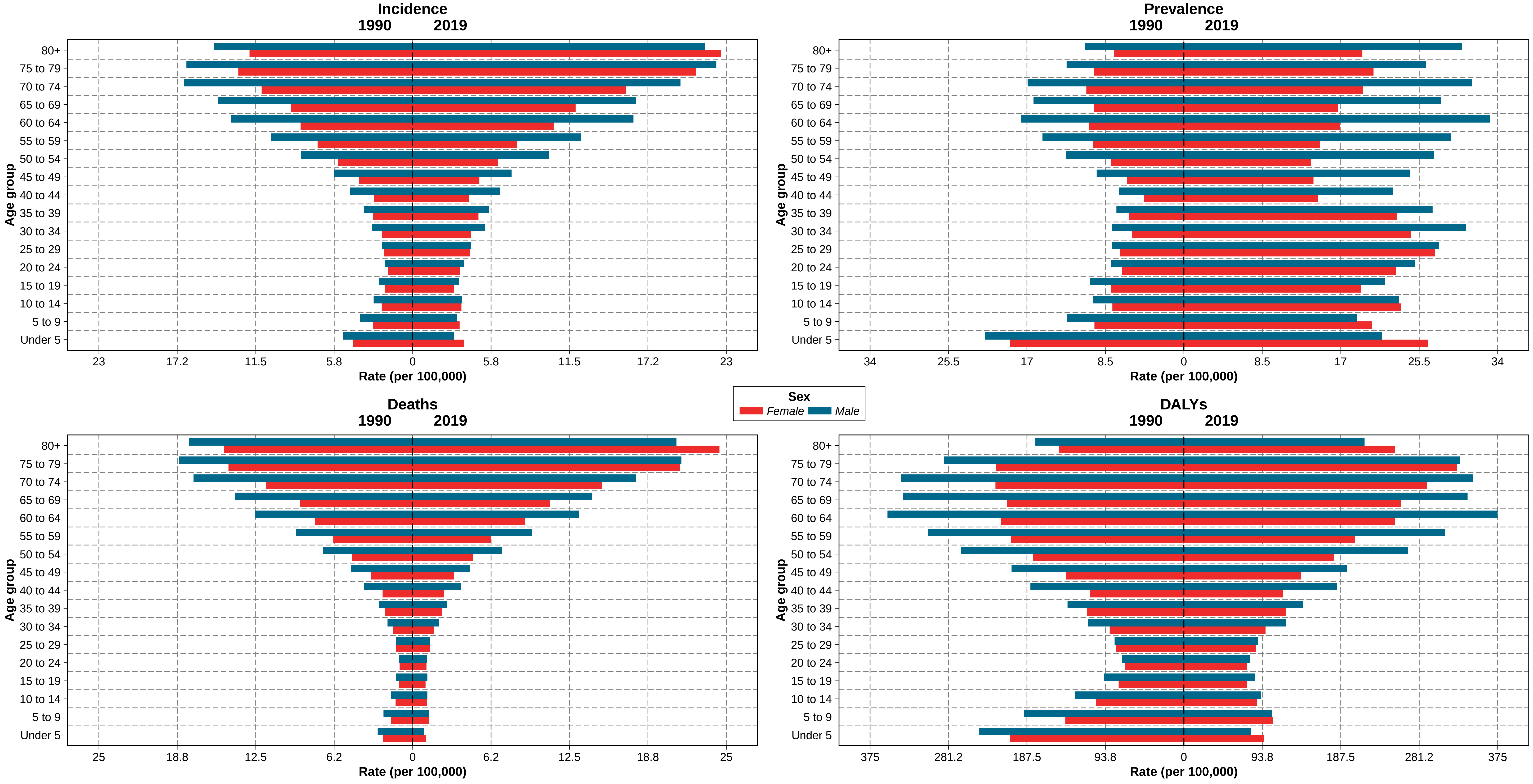

Incidence
1990 2019
Prevalence
1990 2019
Deaths
1990 2019
DALYs
1990 2019
Age group
80+
75 to 79
70 to 74
65 to 69
60 to 64
55 to 59
50 to 54
45 to 49
40 to 44
35 to 39
30 to 34
25 to 29
20 to 24
15 to 19
10 to 14
5 to 9
Under 5
23 17.2 11.5 5.8 0 5.8 11.5 17.2 23
34 25.5 17 8.5 0 8.5 17 25.5 34
25 18.8 12.5 6.2 0 6.2 12.5 18.8 25
375 281.2 187.5 93.8 0 93.8 187.5 281.2 375
Rate (per 100,000)
Sex
Female
Male

Hormozgan

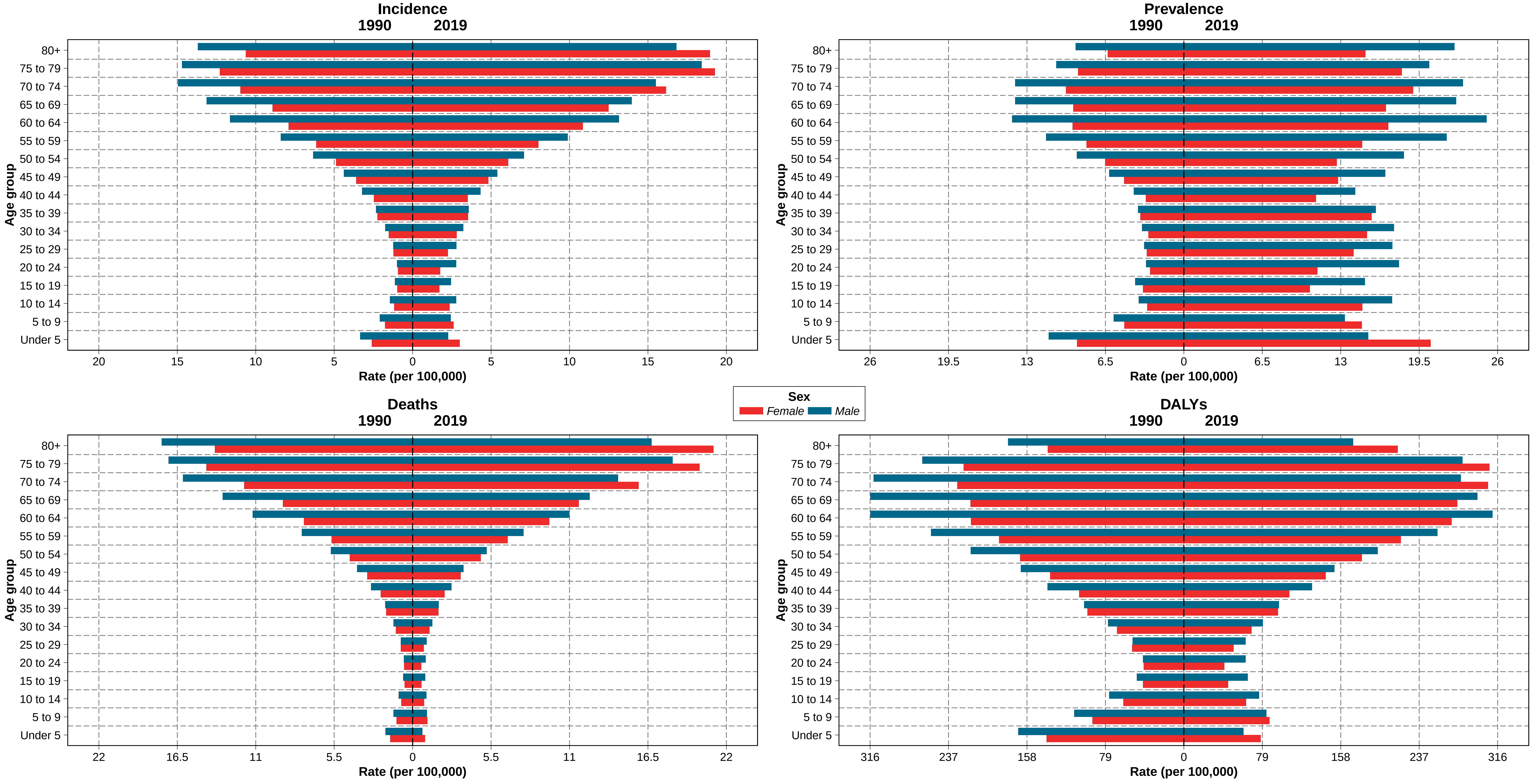

Incidence
1990 2019
80+
75 to 79
70 to 74
65 to 69
60 to 64
55 to 59
50 to 54
45 to 49
40 to 44
35 to 39
30 to 34
25 to 29
20 to 24
15 to 19
10 to 14
5 to 9
Under 5
Age group
20 15 10 5 0 5 10 15 20
Rate (per 100,000)
Prevalence
1990 2019
26 19.5 13 6.5 0 6.5 13 19.5 26
Rate (per 100,000)
Sex
Female
Male
Deaths
1990 2019
22 16.5 11 5.5 0 5.5 11 16.5 22
Rate (per 100,000)
DALYs
1990 2019
316 237 158 79 0 79 158 237 316
Rate (per 100,000)

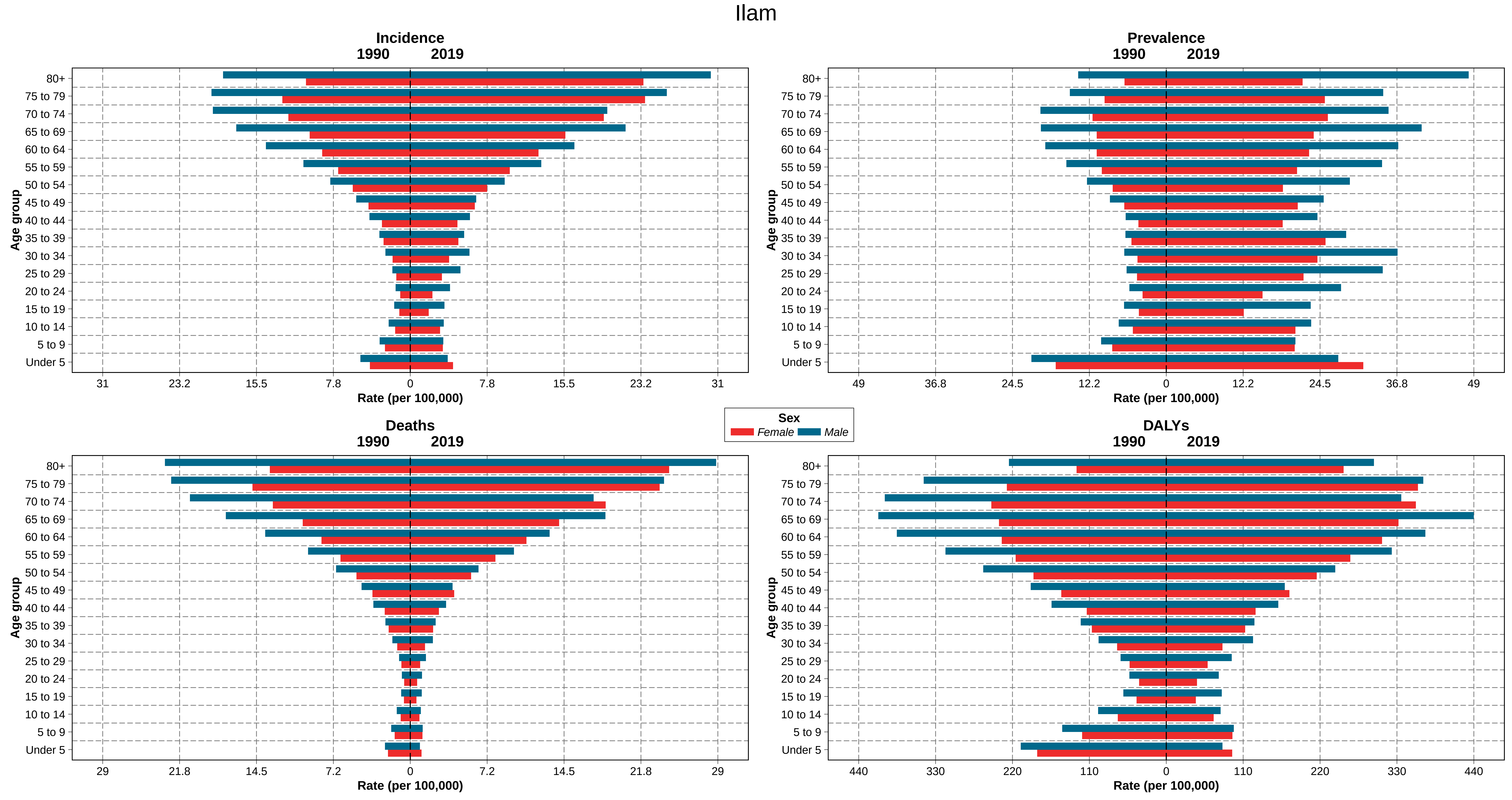
Ilam
Incidence
1990
2019
Prevalence
1990
2019
Deaths
1990
2019
DALYs
1990
2019
Age group
80+
75 to 79
70 to 74
65 to 69
60 to 64
55 to 59
50 to 54
45 to 49
40 to 44
35 to 39
30 to 34
25 to 29
20 to 24
15 to 19
10 to 14
5 to 9
Under 5
Rate (per 100,000)
31
23.2
15.5
7.8
0
7.8
15.5
23.2
31
49
36.8
24.5
12.2
0
12.2
24.5
36.8
49
29
21.8
14.5
7.2
0
7.2
14.5
21.8
29
440
330
220
110
0
110
220
330
440
Sex
Female
Male

Isfahan

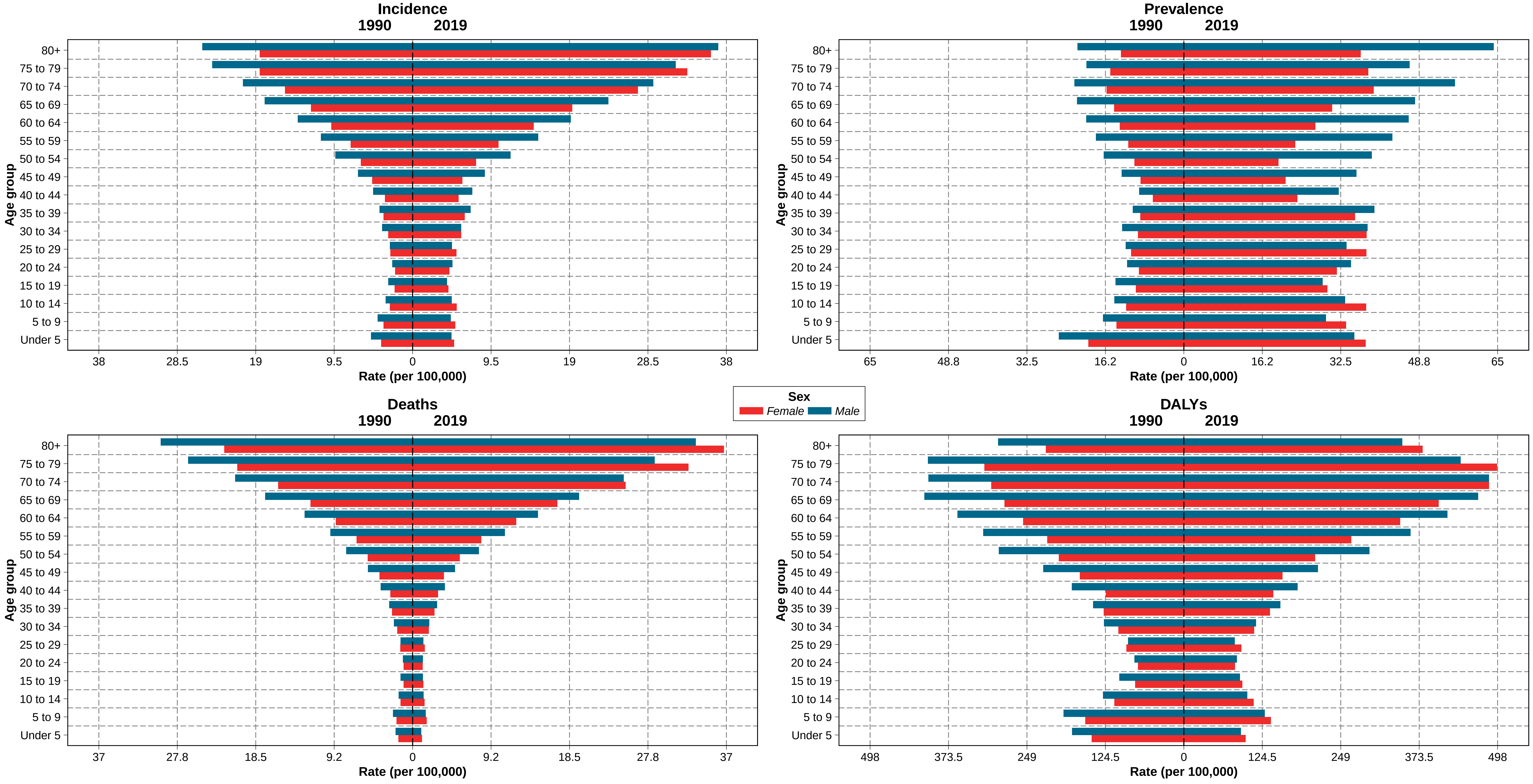

Incidence
1990 2019
80+
75 to 79
70 to 74
65 to 69
60 to 64
55 to 59
50 to 54
45 to 49
40 to 44
35 to 39
30 to 34
25 to 29
20 to 24
15 to 19
10 to 14
5 to 9
Under 5
Age group
38 28.5 19 9.5 0 9.5 19 28.5 38
Rate (per 100,000)
Prevalence
1990 2019
65 48.8 32.5 16.2 0 16.2 32.5 48.8 65
Rate (per 100,000)
Sex
Female Male
Deaths
1990 2019
37 27.8 18.5 9.2 0 9.2 18.5 27.8 37
Rate (per 100,000)
DALYs
1990 2019
498 373.5 249 124.5 0 124.5 249 373.5 498
Rate (per 100,000)

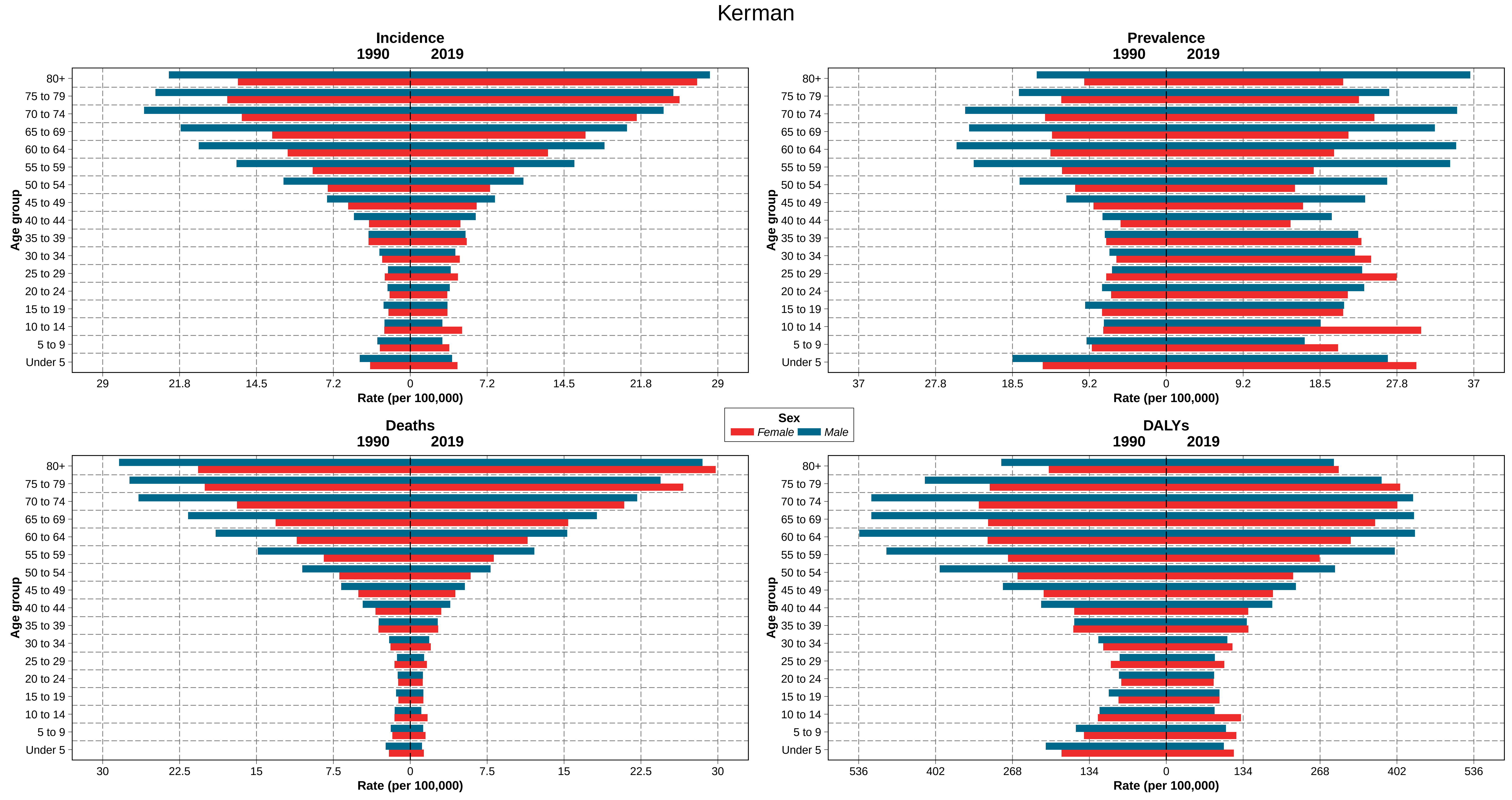

Kerman
Incidence
1990
2019
Prevalence
1990
2019
Deaths
1990
2019
DALYs
1990
2019
80+
75 to 79
70 to 74
65 to 69
60 to 64
55 to 59
50 to 54
45 to 49
40 to 44
35 to 39
30 to 34
25 to 29
20 to 24
15 to 19
10 to 14
5 to 9
Under 5
Age group
29 21.8 14.5 7.2 0 7.2 14.5 21.8 29
37 27.8 18.5 9.2 0 9.2 18.5 27.8 37
30 22.5 15 7.5 0 7.5 15 22.5 30
536 402 268 134 0 134 268 402 536
Rate (per 100,000)
Sex
Female
Male

Kermanshah

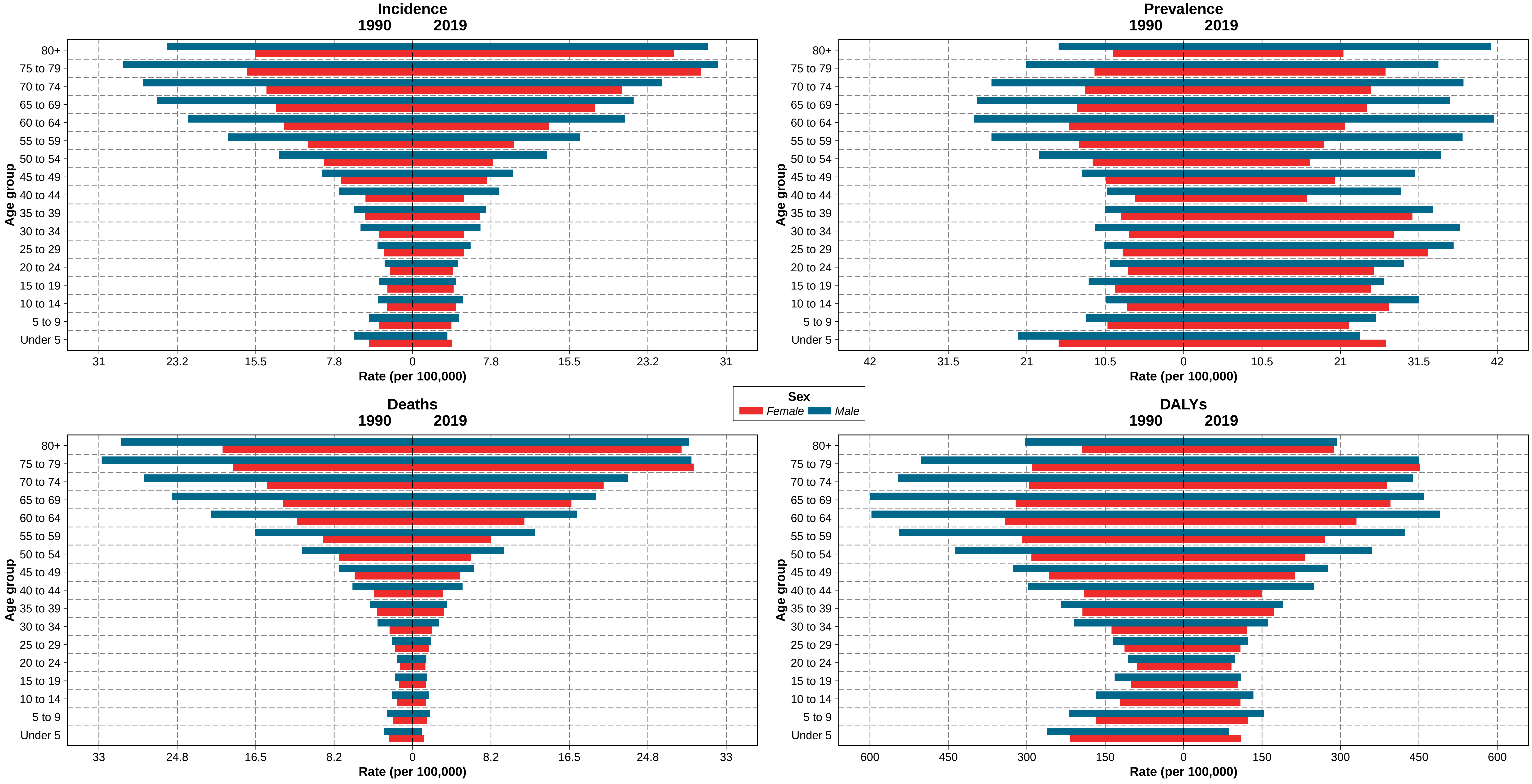
Incidence
1990 2019
Prevalence
1990 2019
Deaths
1990 2019
DALYs
1990 2019
Age group
80+
75 to 79
70 to 74
65 to 69
60 to 64
55 to 59
50 to 54
45 to 49
40 to 44
35 to 39
30 to 34
25 to 29
20 to 24
15 to 19
10 to 14
5 to 9
Under 5
31 23.2 15.5 7.8 0 7.8 15.5 23.2 31
42 31.5 21 10.5 0 10.5 21 31.5 42
33 24.8 16.5 8.2 0 8.2 16.5 24.8 33
600 450 300 150 0 150 300 450 600
Rate (per 100,000)
Sex
Female
Male

Khorasan−e−Razavi

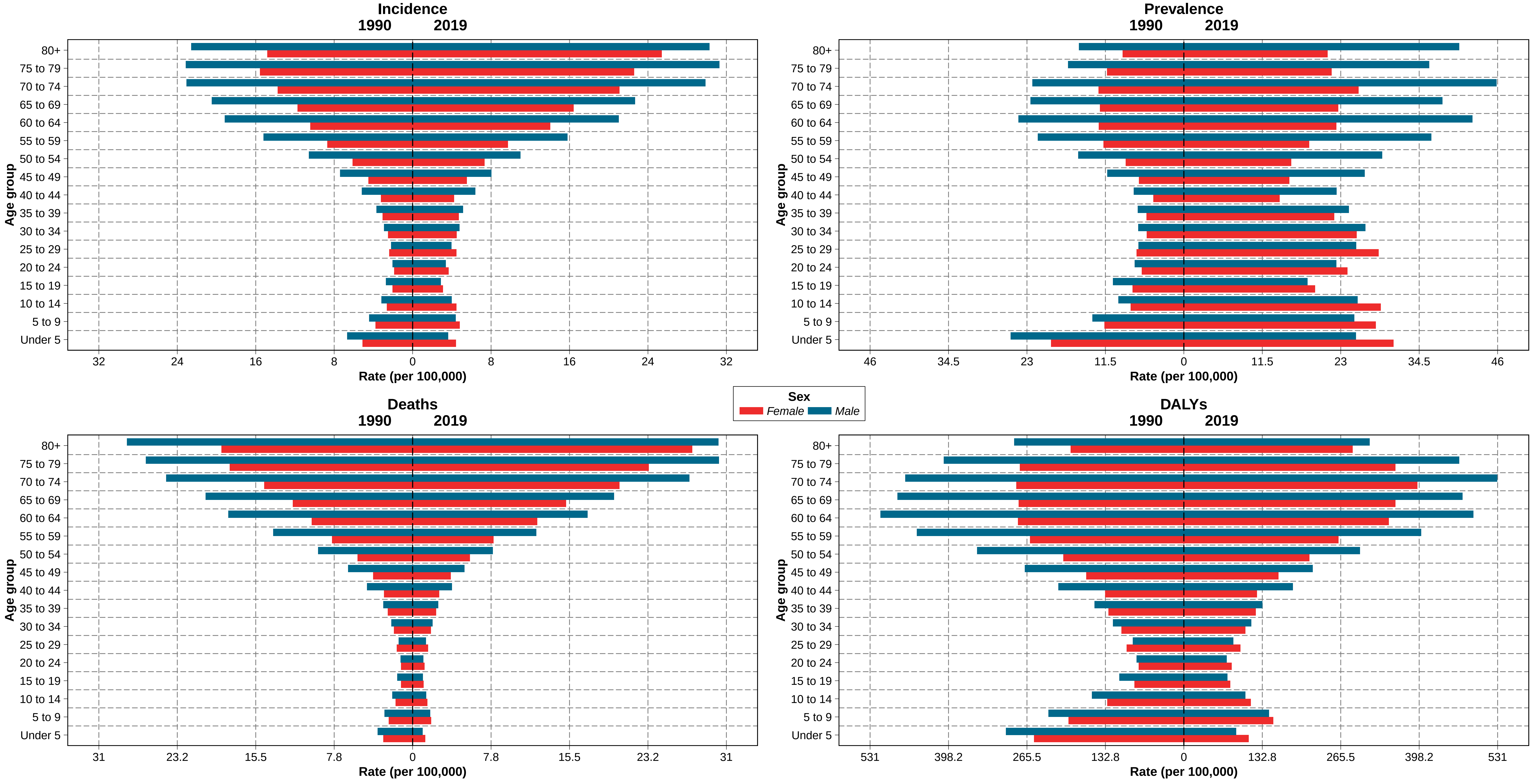

Khuzestan

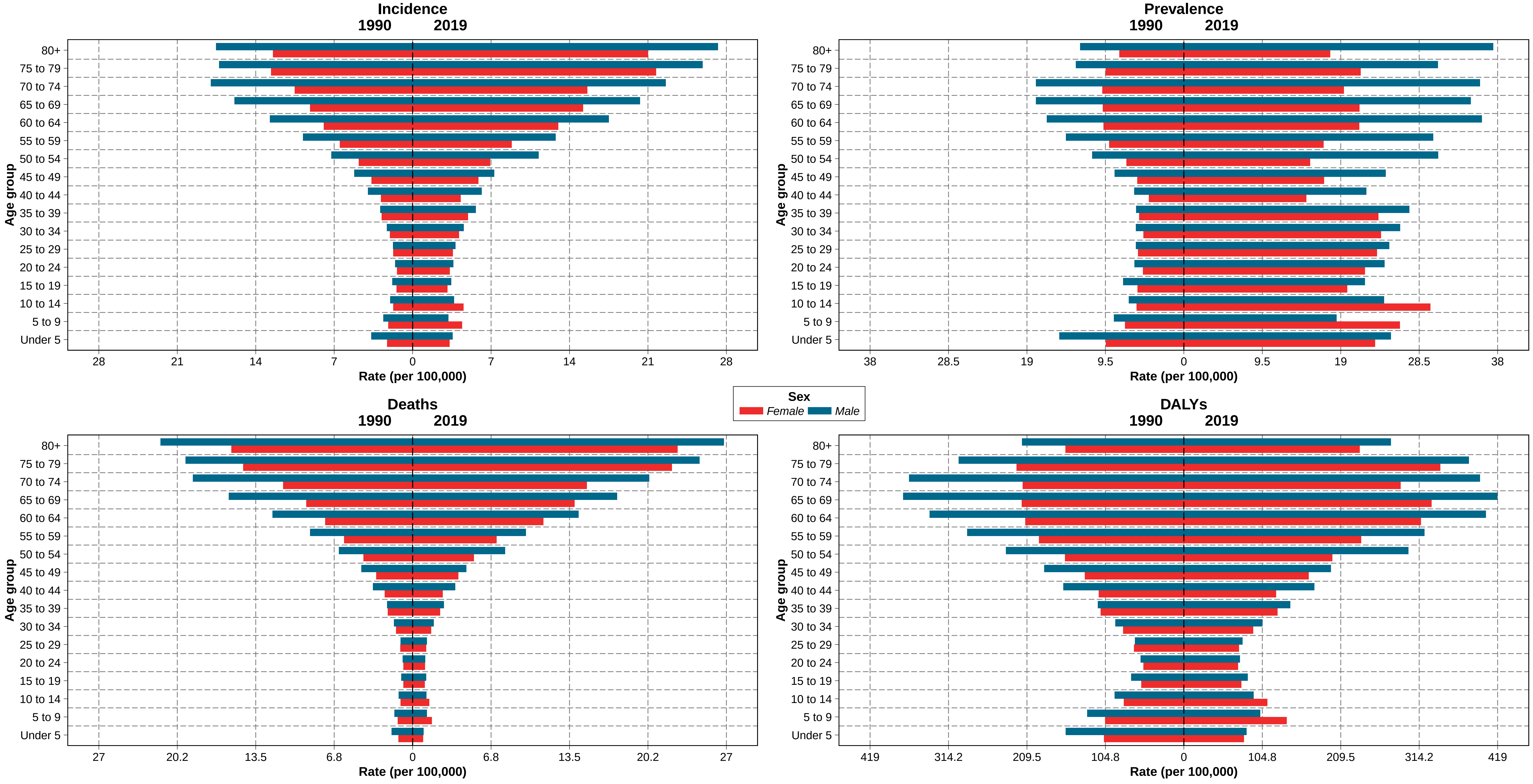
Incidence
1990
2019
80+
75 to 79
70 to 74
65 to 69
60 to 64
55 to 59
50 to 54
45 to 49
40 to 44
35 to 39
30 to 34
25 to 29
20 to 24
15 to 19
10 to 14
5 to 9
Under 5
Age group
28 21 14 7 0 7 14 21 28
Rate (per 100,000)
Prevalence
1990
2019
38 28.5 19 9.5 0 9.5 19 28.5 38
Rate (per 100,000)
Sex
Female
Male
Deaths
1990
2019
27 20.2 13.5 6.8 0 6.8 13.5 20.2 27
Rate (per 100,000)
DALYs
1990
2019
419 314.2 209.5 104.8 0 104.8 209.5 314.2 419
Rate (per 100,000)

Kohgiluyeh and Boyer−Ahmad

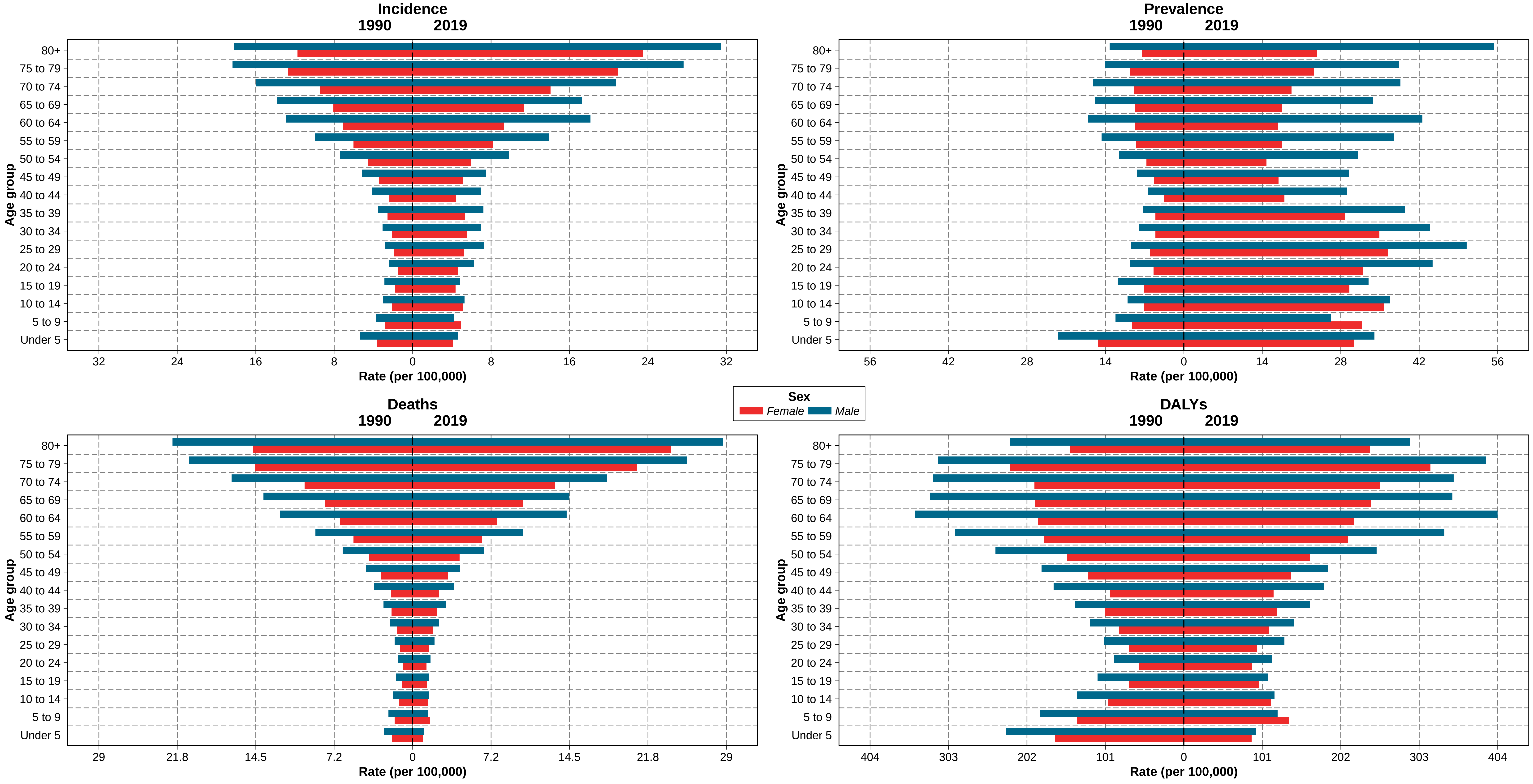

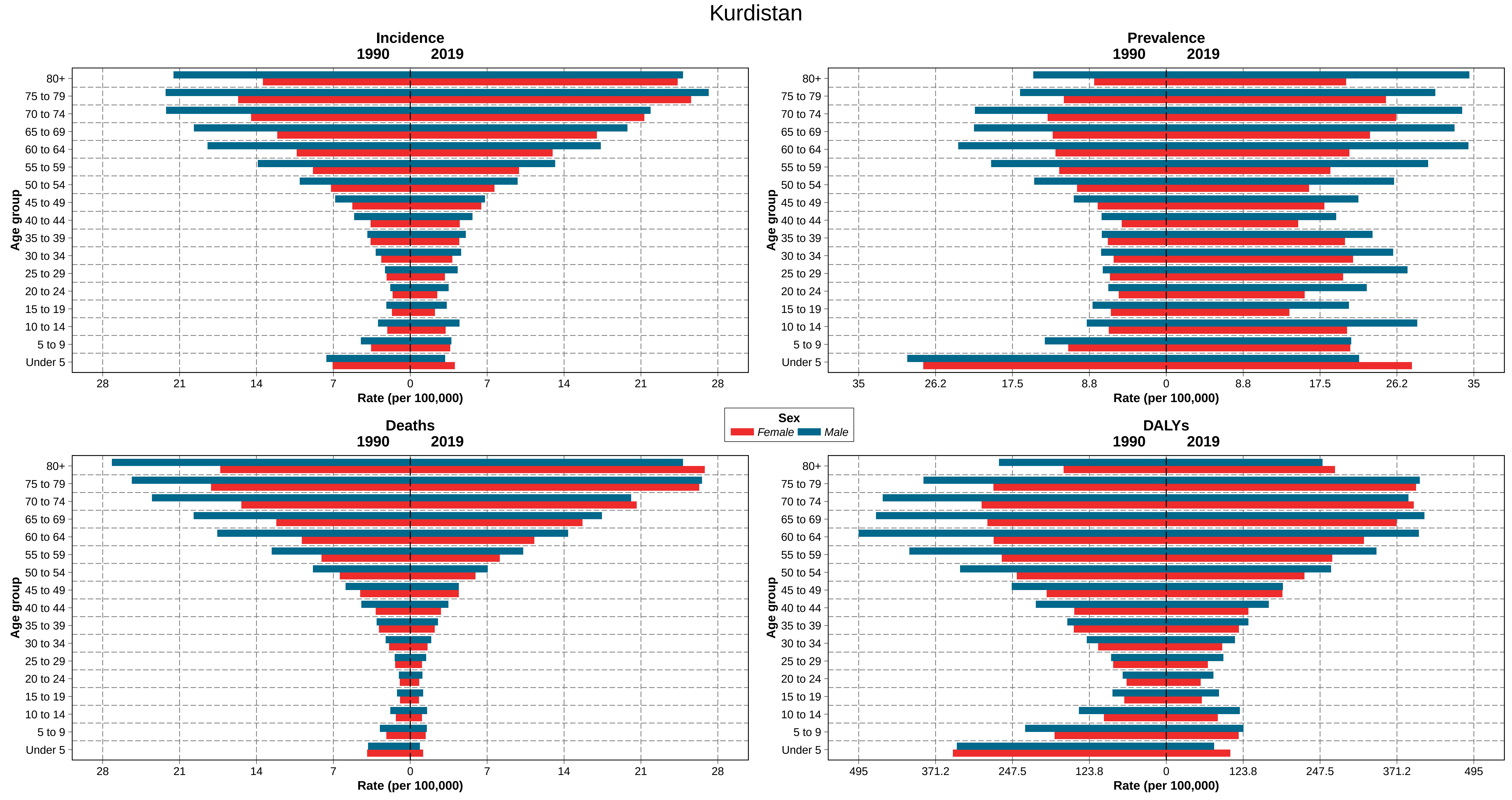
Kurdistan
Incidence
1990
2019
Prevalence
Deaths
DALYs
Age group
80+
75 to 79
70 to 74
65 to 69
60 to 64
55 to 59
50 to 54
45 to 49
40 to 44
35 to 39
30 to 34
25 to 29
20 to 24
15 to 19
10 to 14
5 to 9
Under 5
28
21
14
7
0
35
26.2
17.5
8.8
495
371.2
247.5
123.8
Rate (per 100,000)
Sex
Female
Male

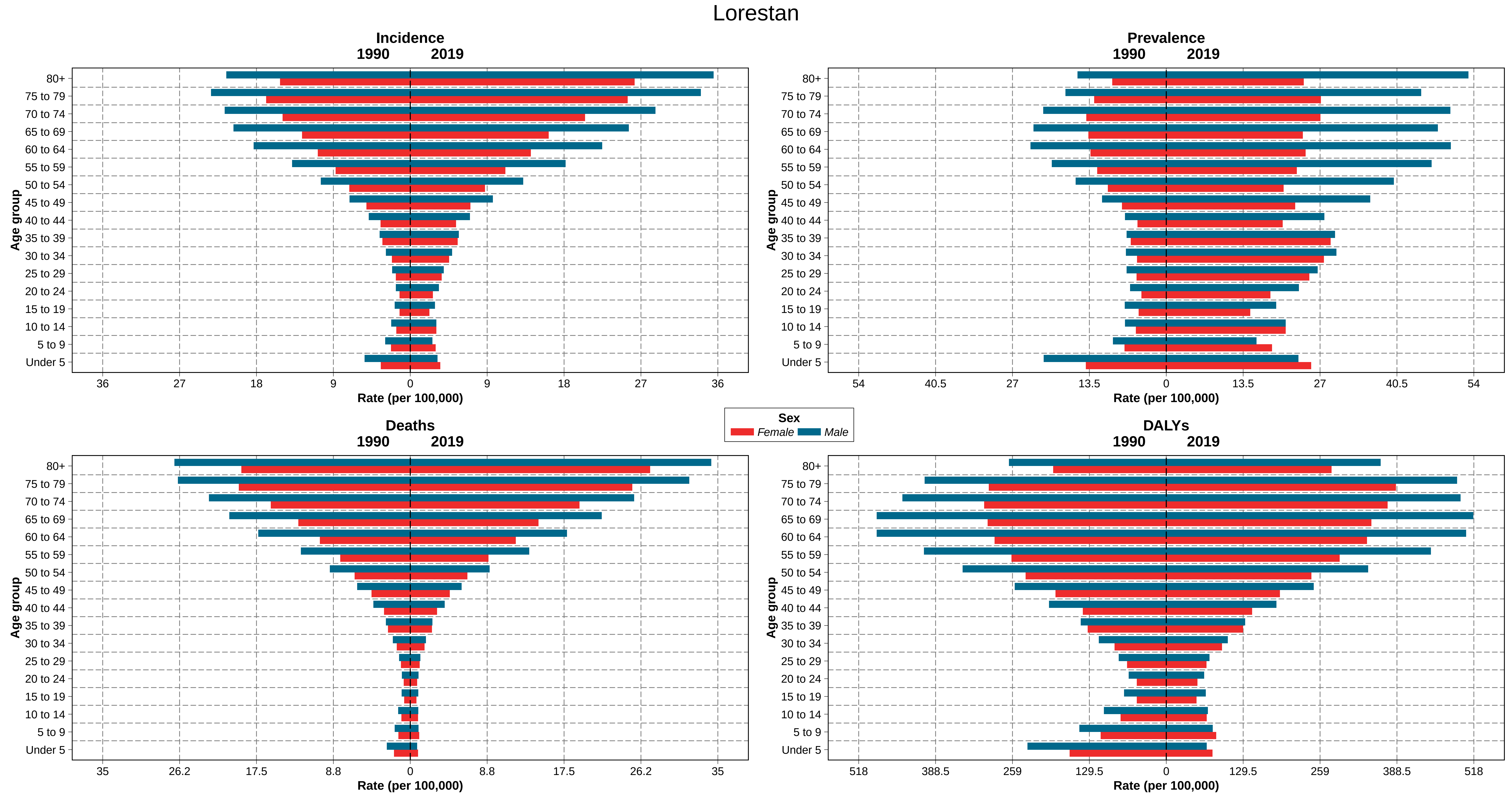

Lorestan
Incidence
1990
2019
Prevalence
1990
2019
Deaths
1990
2019
DALYs
1990
2019
Age group
80+
75 to 79
70 to 74
65 to 69
60 to 64
55 to 59
50 to 54
45 to 49
40 to 44
35 to 39
30 to 34
25 to 29
20 to 24
15 to 19
10 to 14
5 to 9
Under 5
36 27 18 9 0 9 18 27 36
54 40.5 27 13.5 0 13.5 27 40.5 54
35 26.2 17.5 8.8 0 8.8 17.5 26.2 35
518 388.5 259 129.5 0 129.5 259 388.5 518
Rate (per 100,000)
Sex
Female
Male

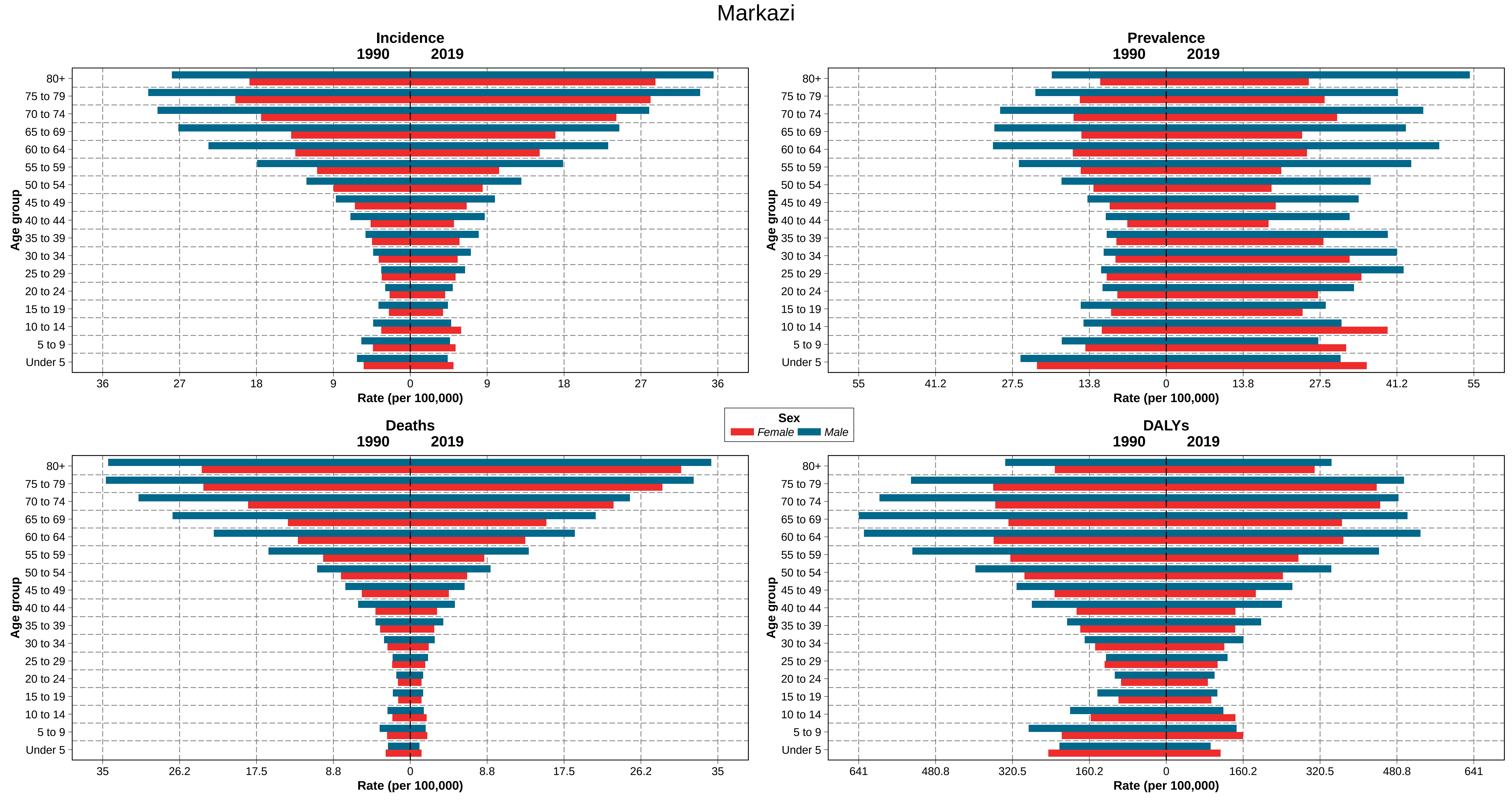
Markazi
Incidence
1990 2019
Prevalence
1990 2019
Deaths
1990 2019
DALYs
1990 2019
Age group
80+
75 to 79
70 to 74
65 to 69
60 to 64
55 to 59
50 to 54
45 to 49
40 to 44
35 to 39
30 to 34
25 to 29
20 to 24
15 to 19
10 to 14
5 to 9
Under 5
36 27 18 9 0 9 18 27 36
55 41.2 27.5 13.8 0 13.8 27.5 41.2 55
35 26.2 17.5 8.8 0 8.8 17.5 26.2 35
641 480.8 320.5 160.2 0 160.2 320.5 480.8 641
Rate (per 100,000)
Sex
Female
Male

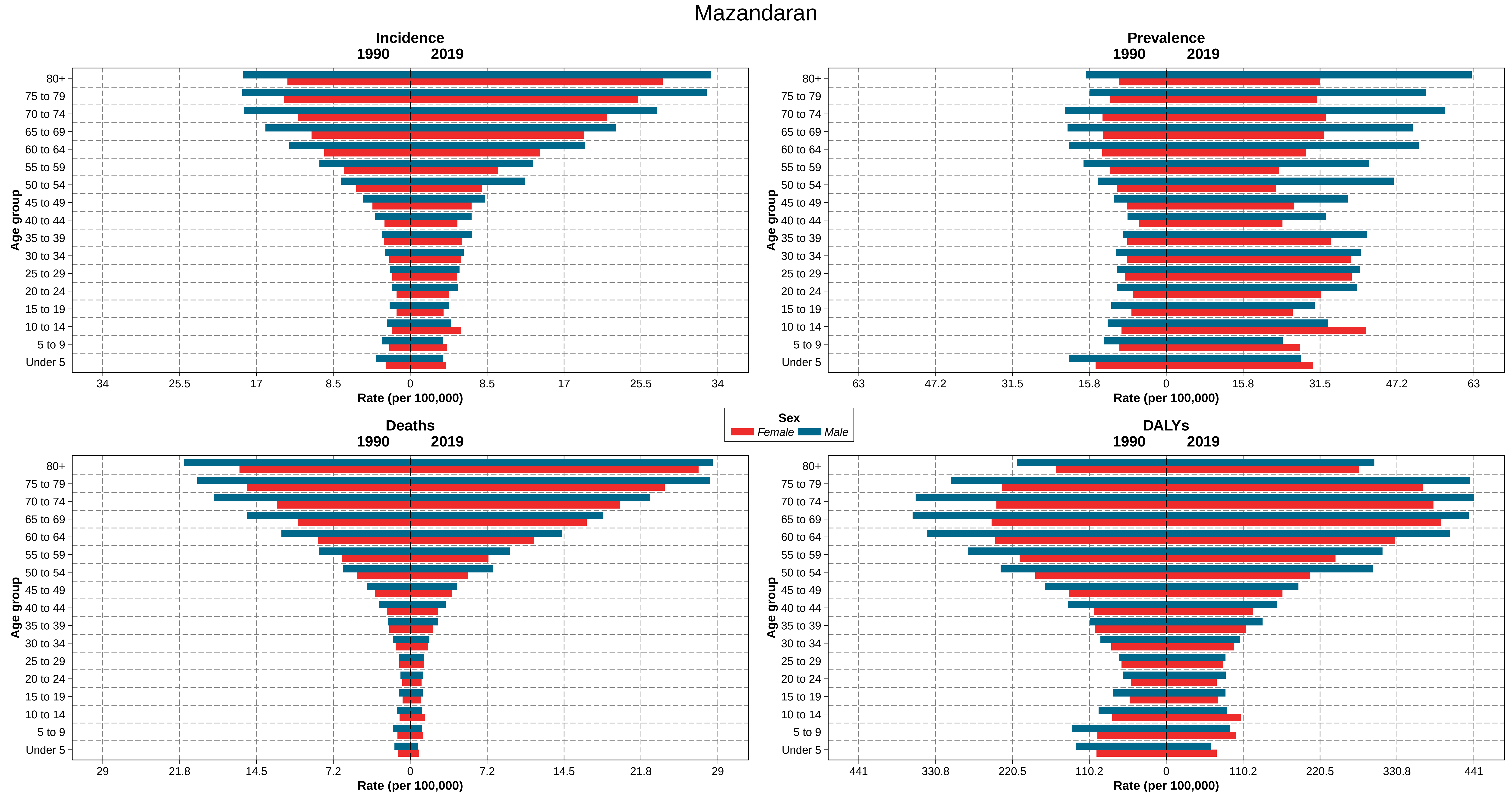

Mazandaran
Incidence
1990
2019
Prevalence
1990
2019
Deaths
1990
2019
DALYs
1990
2019
Age group
80+
75 to 79
70 to 74
65 to 69
60 to 64
55 to 59
50 to 54
45 to 49
40 to 44
35 to 39
30 to 34
25 to 29
20 to 24
15 to 19
10 to 14
5 to 9
Under 5
Rate (per 100,000)
34
25.5
17
8.5
0
8.5
17
25.5
34
63
47.2
31.5
15.8
0
15.8
31.5
47.2
63
29
21.8
14.5
7.2
0
7.2
14.5
21.8
29
441
330.8
220.5
110.2
0
110.2
220.5
330.8
441
Sex
Female
Male

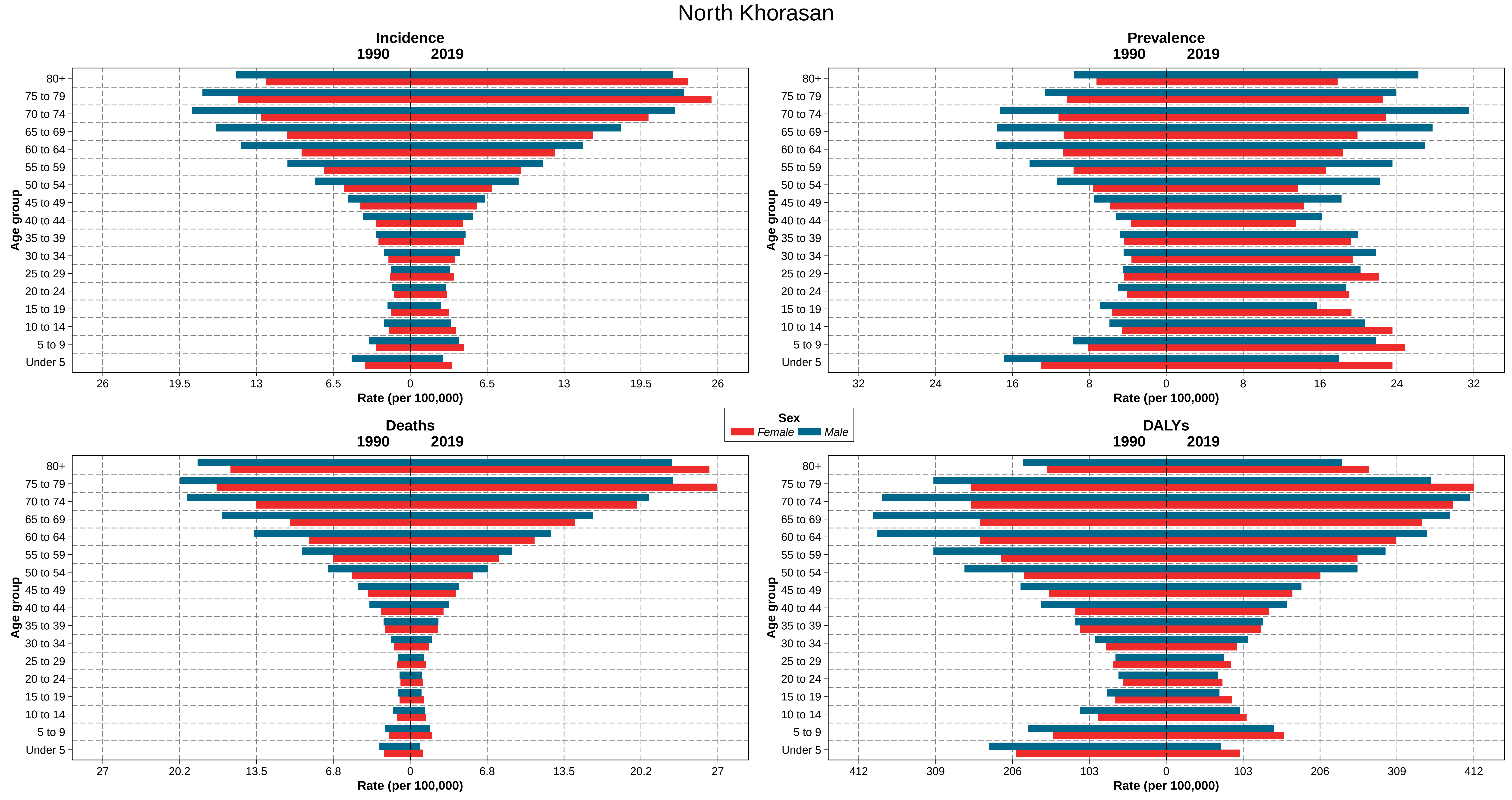
North Khorasan
Incidence
1990
2019
Prevalence
1990
2019
Deaths
1990
2019
DALYs
1990
2019
Age group
80+
75 to 79
70 to 74
65 to 69
60 to 64
55 to 59
50 to 54
45 to 49
40 to 44
35 to 39
30 to 34
25 to 29
20 to 24
15 to 19
10 to 14
5 to 9
Under 5
26 19.5 13 6.5 0 6.5 13 19.5 26
32 24 16 8 0 8 16 24 32
27 20.2 13.5 6.8 0 6.8 13.5 20.2 27
412 309 206 103 0 103 206 309 412
Rate (per 100,000)
Sex
Female
Male

Qazvin

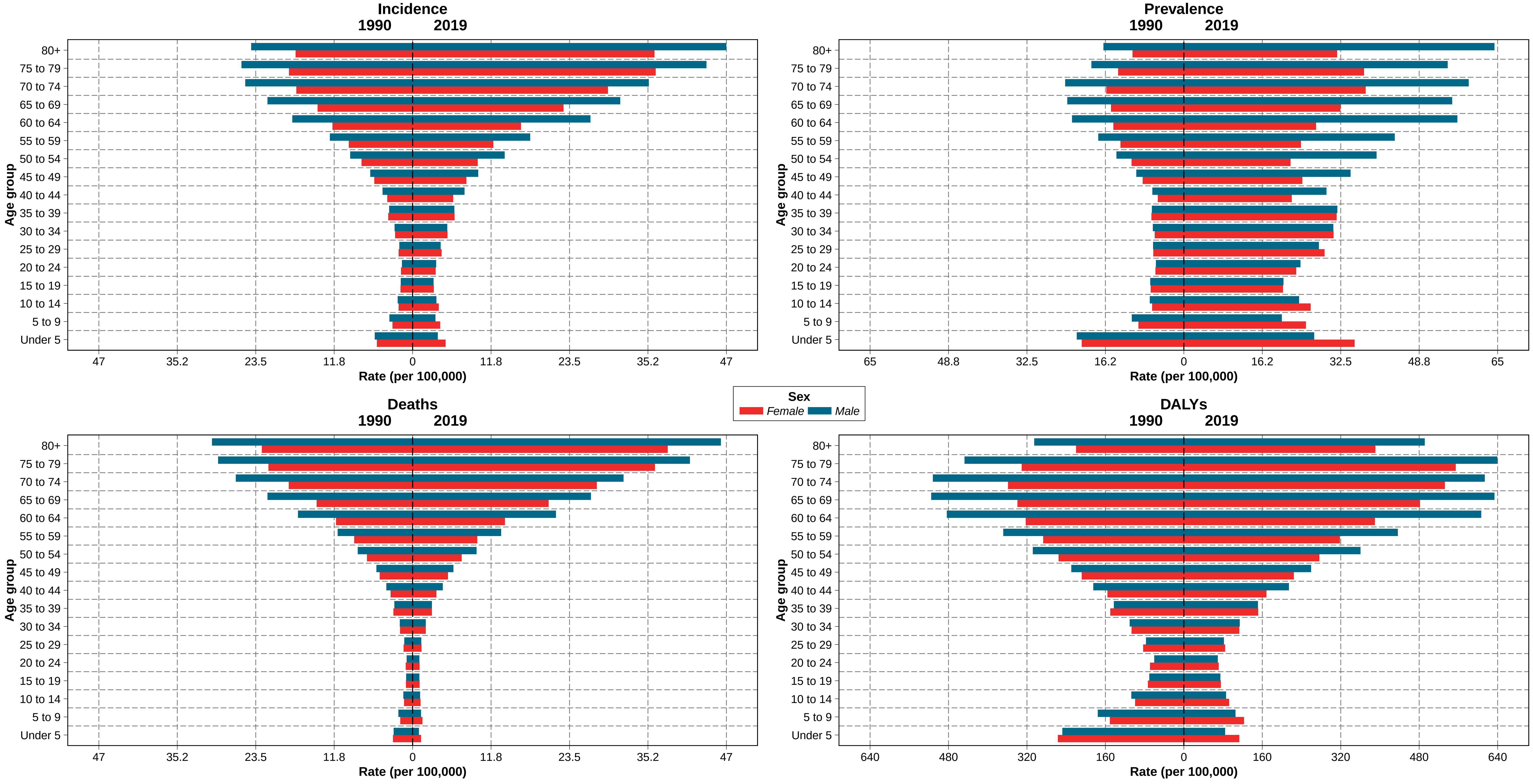
Incidence
1990
2019
Prevalence
1990
2019
Deaths
1990
2019
DALYs
1990
2019
Age group
80+
75 to 79
70 to 74
65 to 69
60 to 64
55 to 59
50 to 54
45 to 49
40 to 44
35 to 39
30 to 34
25 to 29
20 to 24
15 to 19
10 to 14
5 to 9
Under 5
47 35.2 23.5 11.8 0 11.8 23.5 35.2 47
65 48.8 32.5 16.2 0 16.2 32.5 48.8 65
640 480 320 160 0 160 320 480 640
Rate (per 100,000)
Sex
Female
Male

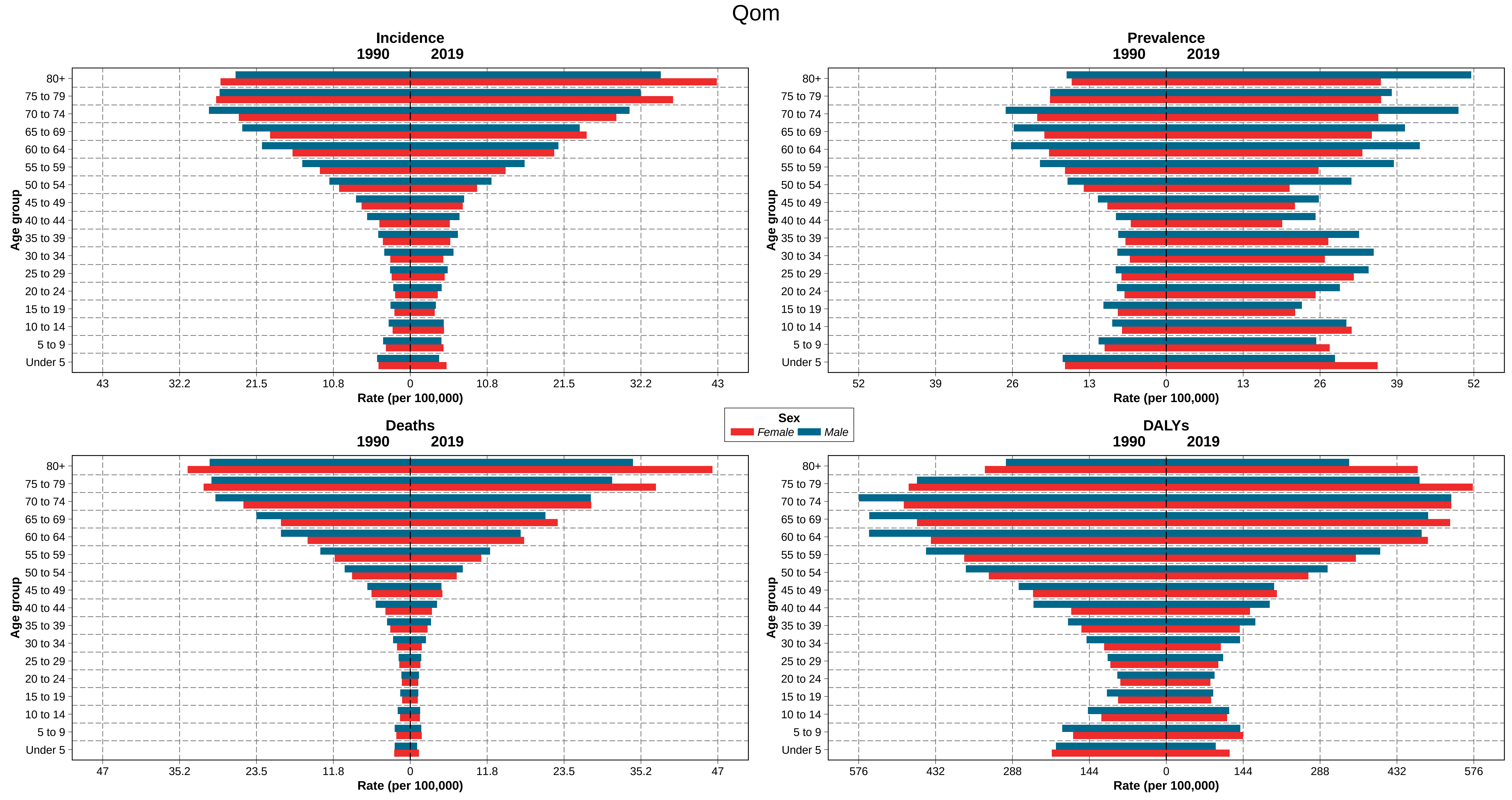

Qom
Incidence
1990 2019
Prevalence
1990 2019
Deaths
1990 2019
DALYs
1990 2019
Sex
Female
Male
Age group
80+
75 to 79
70 to 74
65 to 69
60 to 64
55 to 59
50 to 54
45 to 49
40 to 44
35 to 39
30 to 34
25 to 29
20 to 24
15 to 19
10 to 14
5 to 9
Under 5
Rate (per 100,000)
43 32.2 21.5 10.8 0 10.8 21.5 32.2 43
52 39 26 13 0 13 26 39 52
47 35.2 23.5 11.8 0 11.8 23.5 35.2 47
576 432 288 144 0 144 288 432 576

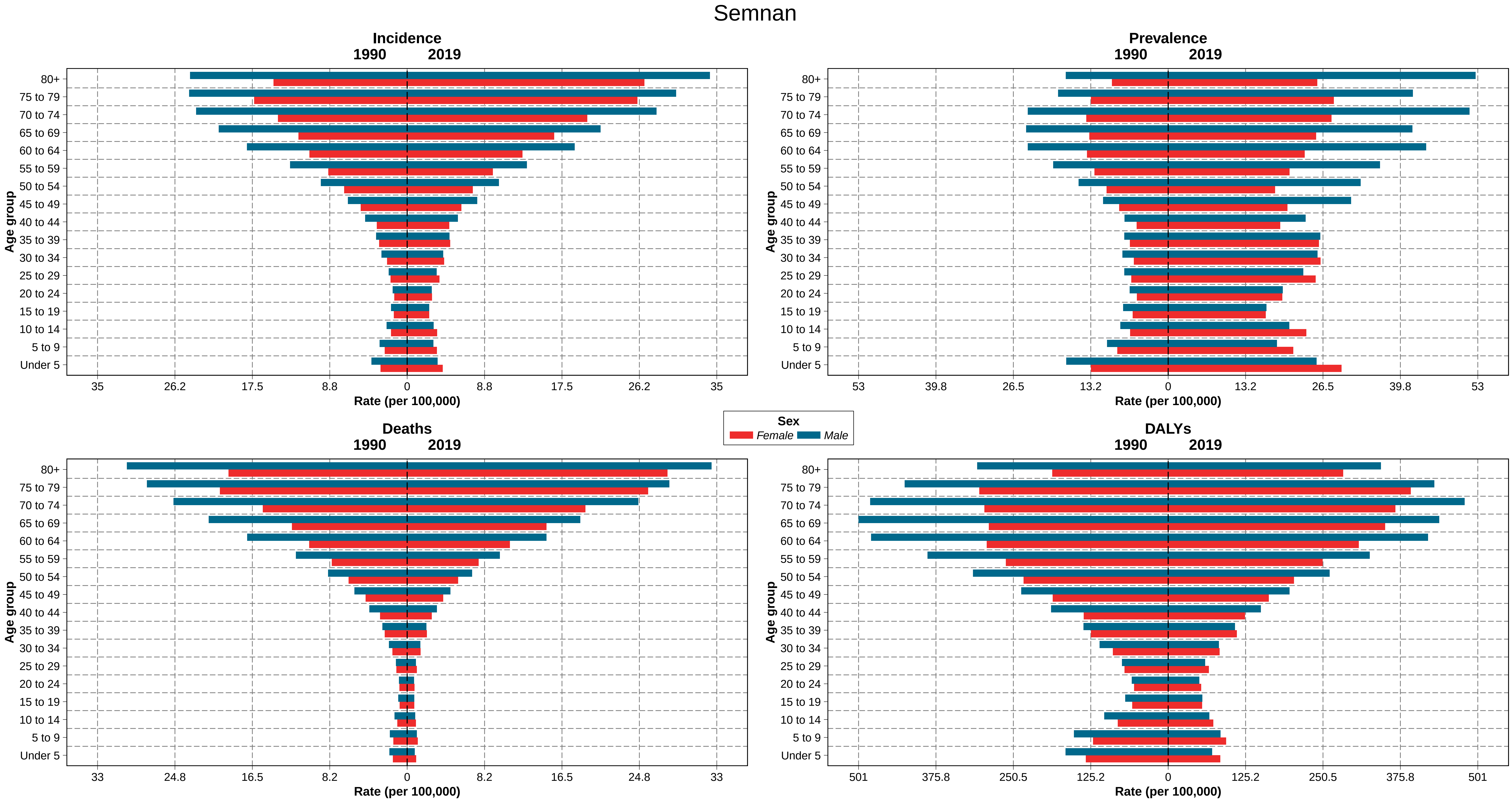
Semnan
Incidence
1990
2019
Prevalence
1990
2019
Deaths
1990
2019
DALYs
1990
2019
Age group
80+
75 to 79
70 to 74
65 to 69
60 to 64
55 to 59
50 to 54
45 to 49
40 to 44
35 to 39
30 to 34
25 to 29
20 to 24
15 to 19
10 to 14
5 to 9
Under 5
35 26.2 17.5 8.8 0 8.8 17.5 26.2 35
53 39.8 26.5 13.2 0 13.2 26.5 39.8 53
33 24.8 16.5 8.2 0 8.2 16.5 24.8 33
501 375.8 250.5 125.2 0 125.2 250.5 375.8 501
Rate (per 100,000)
Sex
Female
Male

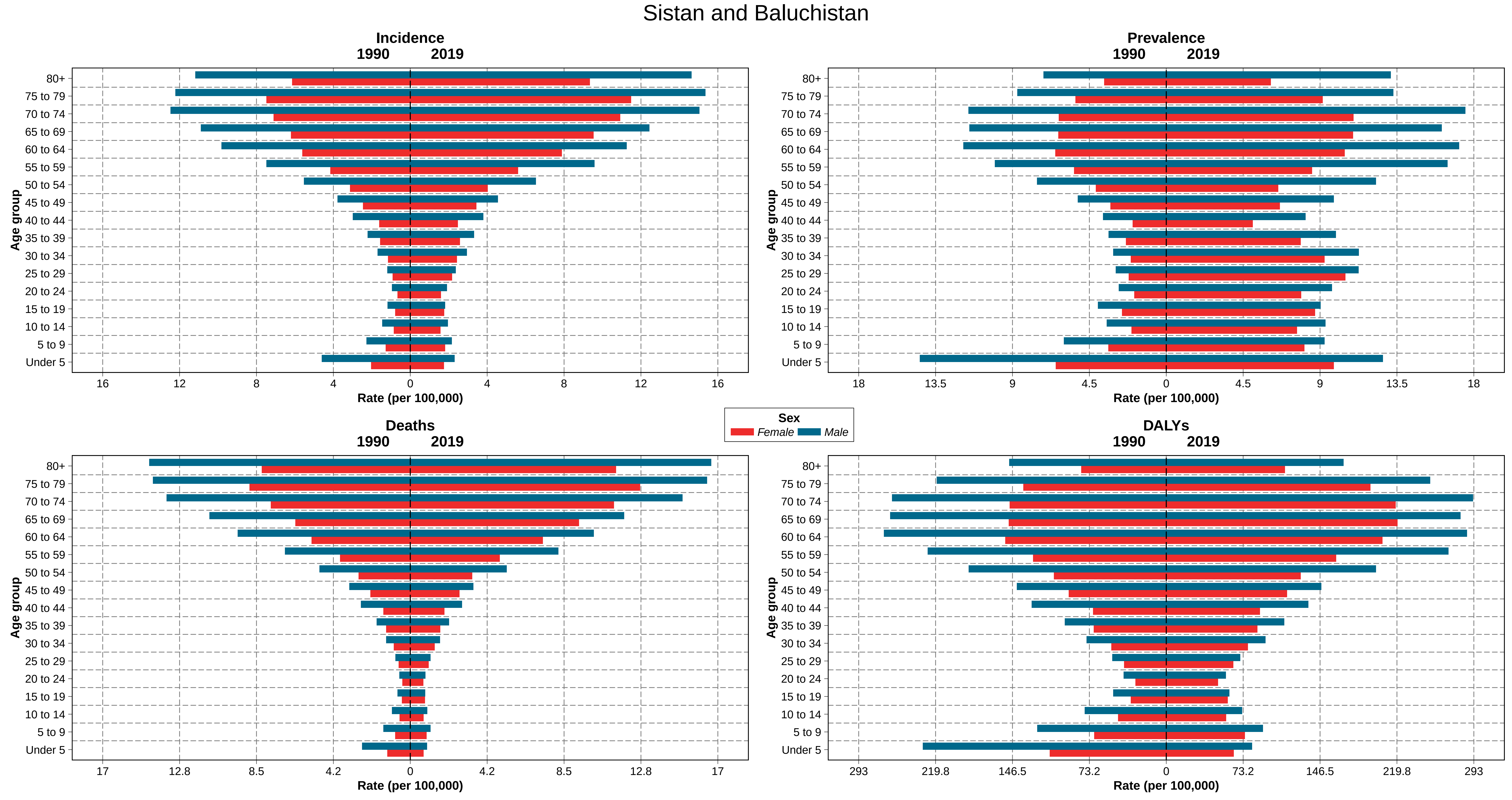

Sistan and Baluchistan
Incidence
Prevalence
Deaths
DALYs
1990
2019
Age group
80+
75 to 79
70 to 74
65 to 69
60 to 64
55 to 59
50 to 54
45 to 49
40 to 44
35 to 39
30 to 34
25 to 29
20 to 24
15 to 19
10 to 14
5 to 9
Under 5
16 12 8 4 0 4 8 12 16
18 13.5 9 4.5 0 4.5 9 13.5 18
17 12.8 8.5 4.2 0 4.2 8.5 12.8 17
293 219.8 146.5 73.2 0 73.2 146.5 219.8 293
Rate (per 100,000)
Sex
Female
Male

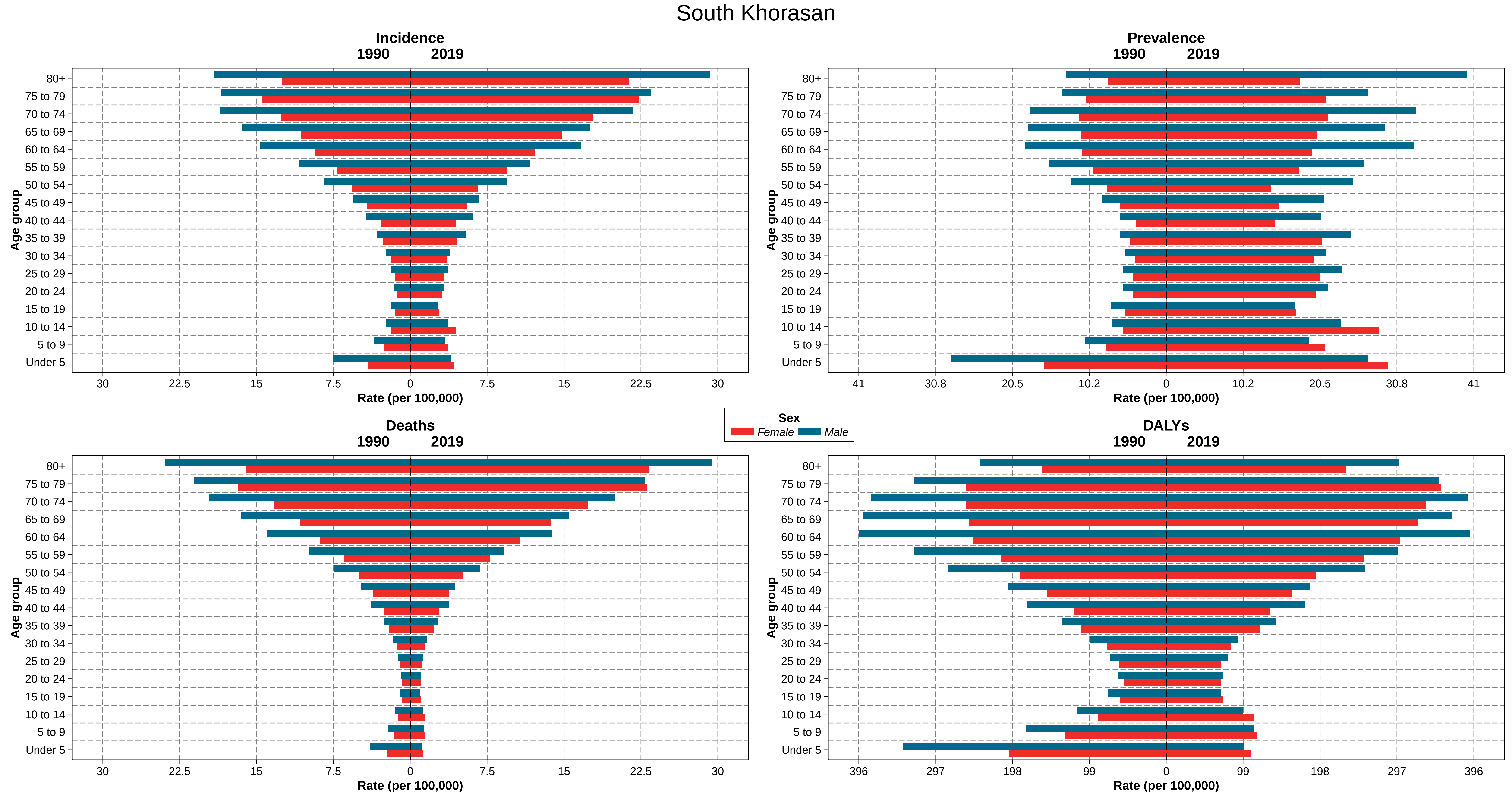
South Khorasan
Incidence
1990
2019
Prevalence
1990
2019
Deaths
1990
2019
DALYs
1990
2019
Age group
80+
75 to 79
70 to 74
65 to 69
60 to 64
55 to 59
50 to 54
45 to 49
40 to 44
35 to 39
30 to 34
25 to 29
20 to 24
15 to 19
10 to 14
5 to 9
Under 5
30 22.5 15 7.5 0 7.5 15 22.5 30
41 30.8 20.5 10.2 0 10.2 20.5 30.8 41
396 297 198 99 0 99 198 297 396
Rate (per 100,000)
Sex
Female
Male

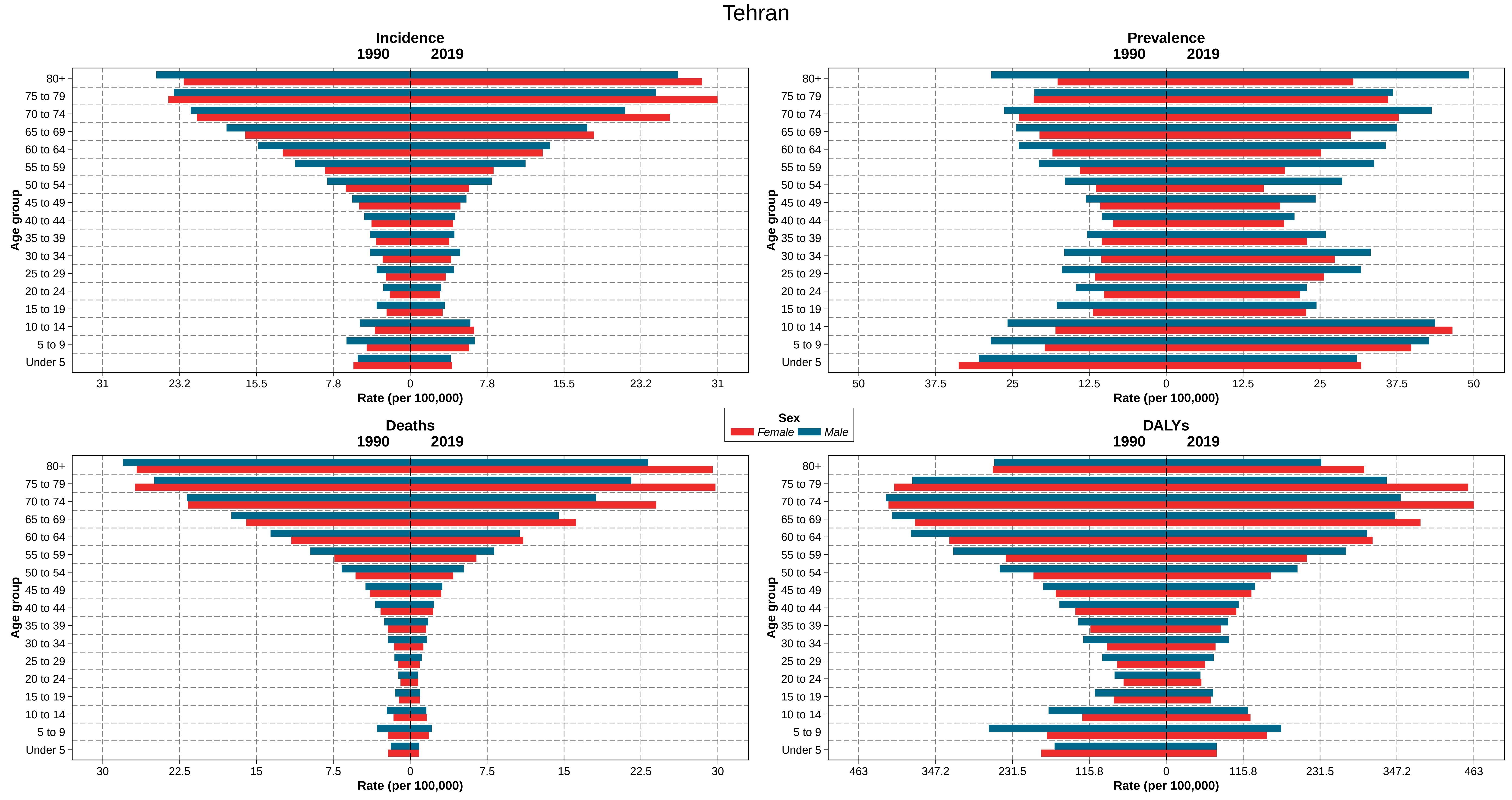

Tehran
Incidence
1990 2019
Prevalence
1990 2019
Deaths
1990 2019
DALYs
1990 2019
Age group
80+
75 to 79
70 to 74
65 to 69
60 to 64
55 to 59
50 to 54
45 to 49
40 to 44
35 to 39
30 to 34
25 to 29
20 to 24
15 to 19
10 to 14
5 to 9
Under 5
31 23.2 15.5 7.8 0 7.8 15.5 23.2 31
50 37.5 25 12.5 0 12.5 25 37.5 50
30 22.5 15 7.5 0 7.5 15 22.5 30
463 347.2 231.5 115.8 0 115.8 231.5 347.2 463
Rate (per 100,000)
Sex
Female
Male

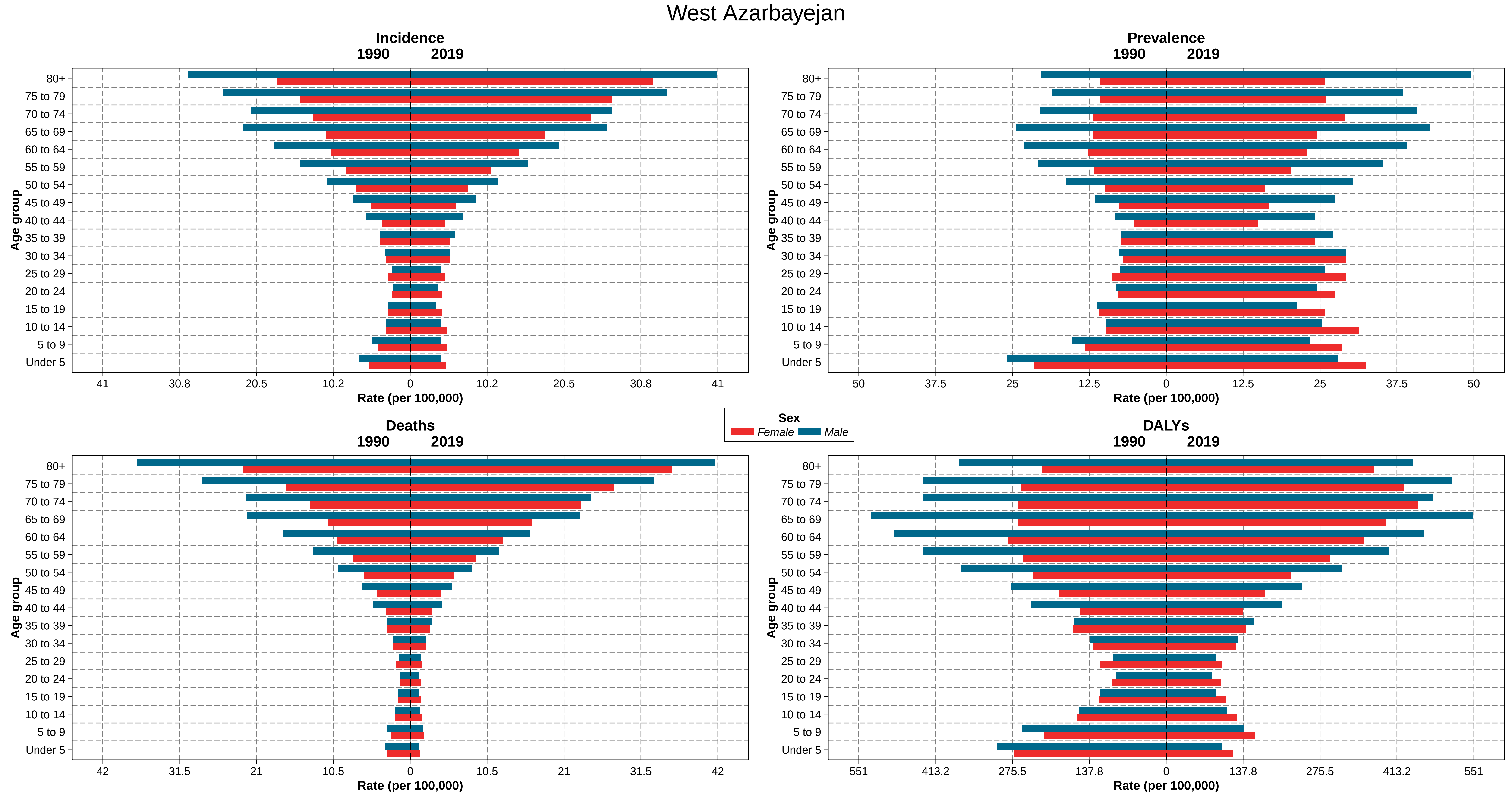
West Azarbayejan
Incidence
1990
2019
Prevalence
1990
2019
Deaths
1990
2019
DALYs
1990
2019
Age group
80+
75 to 79
70 to 74
65 to 69
60 to 64
55 to 59
50 to 54
45 to 49
40 to 44
35 to 39
30 to 34
25 to 29
20 to 24
15 to 19
10 to 14
5 to 9
Under 5
41 30.8 20.5 10.2 0 10.2 20.5 30.8 41
50 37.5 25 12.5 0 12.5 25 37.5 50
42 31.5 21 10.5 0 10.5 21 31.5 42
551 413.2 275.5 137.8 0 137.8 275.5 413.2 551
Rate (per 100,000)
Sex
Female
Male

Yazd

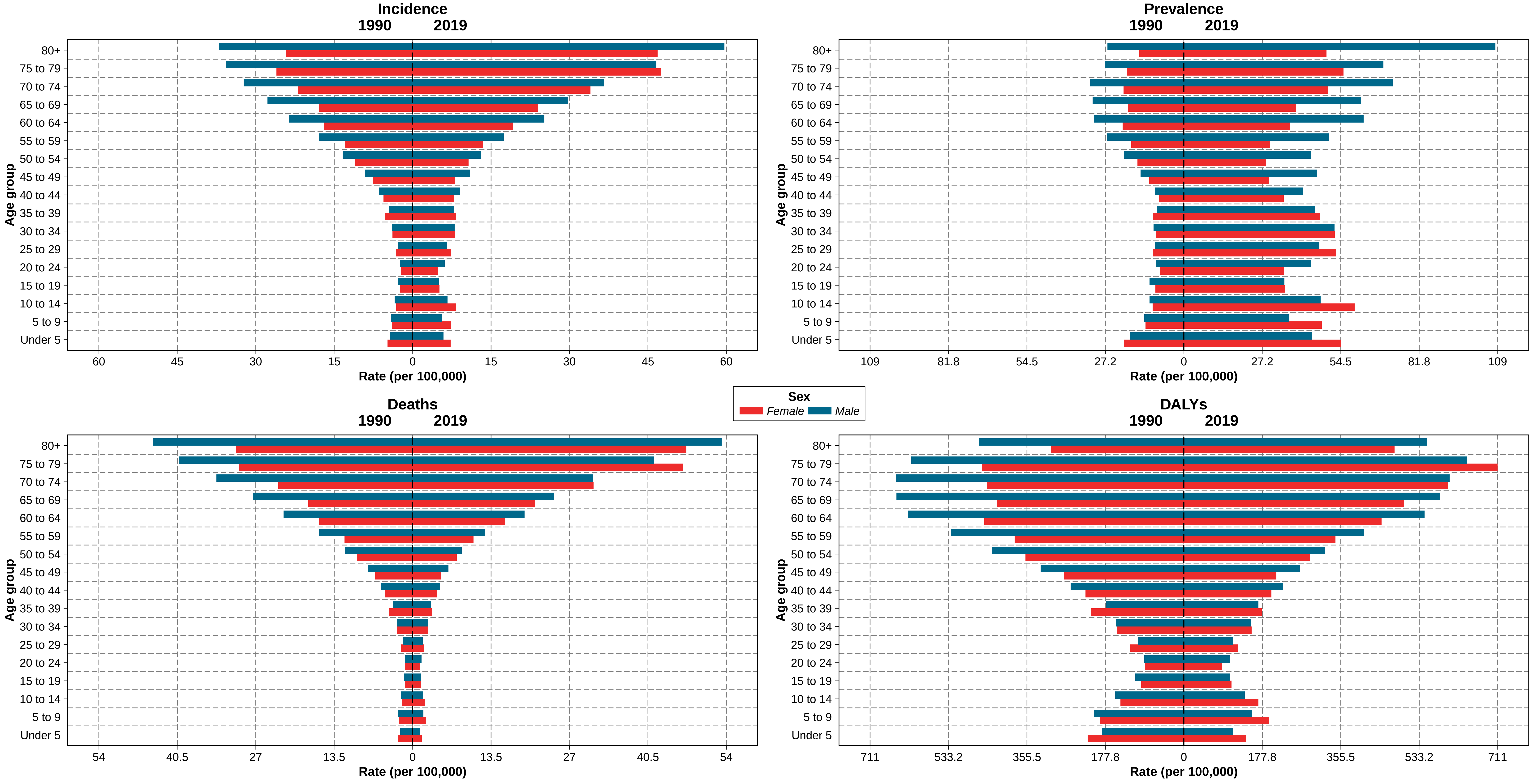
Incidence
1990
2019
Prevalence
Deaths
DALYs
Age group
80+
75 to 79
70 to 74
65 to 69
60 to 64
55 to 59
50 to 54
45 to 49
40 to 44
35 to 39
30 to 34
25 to 29
20 to 24
15 to 19
10 to 14
5 to 9
Under 5
60 45 30 15 0 15 30 45 60
109 81.8 54.5 27.2 0 27.2 54.5 81.8 109
54 40.5 27 13.5 0 13.5 27 40.5 54
711 533.2 355.5 177.8 0 177.8 355.5 533.2 711
Rate (per 100,000)
Sex
Female
Male

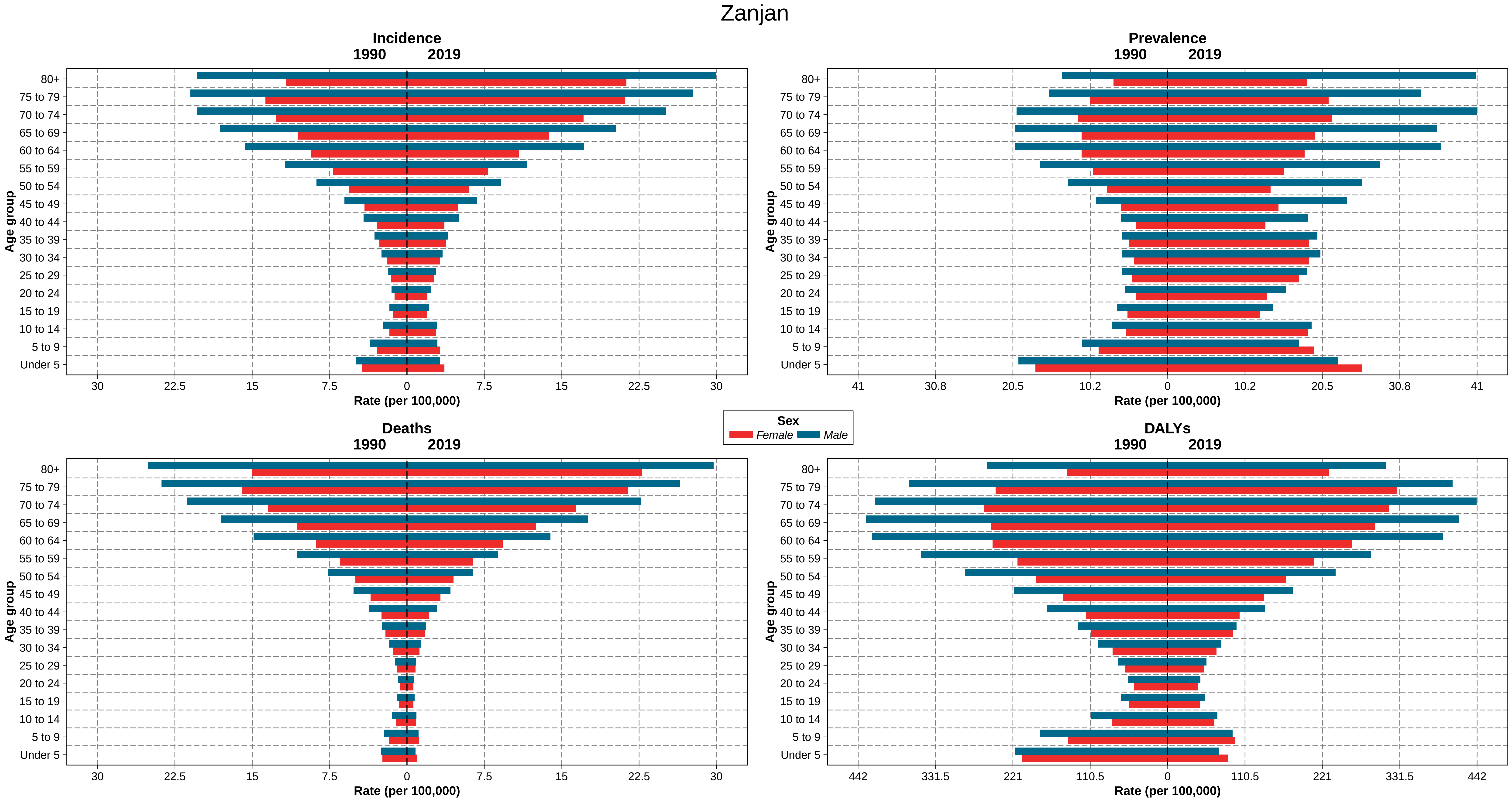
Zanjan
Incidence
Prevalence
Deaths
DALYs
1990
2019
Age group
80+
75 to 79
70 to 74
65 to 69
60 to 64
55 to 59
50 to 54
45 to 49
40 to 44
35 to 39
30 to 34
25 to 29
20 to 24
15 to 19
10 to 14
5 to 9
Under 5
Rate (per 100,000)
30 22.5 15 7.5 0 7.5 15 22.5 30
41 30.8 20.5 10.2 0 10.2 20.5 30.8 41
442 331.5 221 110.5 0 110.5 221 331.5 442
Sex
Female
Male
