# Supplementary material for: National and subnational burden of brain and central nervous system cancers in Iran, 1990–2019: Results from the global burden of disease study 2019
Source: Cancer Med. 2023 Jan 9;12(7):8614–28. doi: 10.1002/cam4.5553 (PMC10134290; doi:10.1002/cam4.5553)
Supplement: Supplementary file 7 — Table S1. [file CAM4-12-8614-s009.pdf]

Supplementary Table 1

Title	Time period covered	Coverage type	Geography	Data type	Secondary data type	Series or system	Contributors	Authors	Journal	Volume	Publication year
Cancer Occurrence in Ardabil: Results of a Population-Based Cancer Registry from Iran	01/1996 to 12/1999	Subnational	Iran (Islamic Republic of), ArdabÄ«l	Scientific literature				Sadjadi A, Malekzadeh R, Derakhshan MH, Sepehr A, Nouraie M, Sotoudeh M, Yazdanbod A, Shokoohi B, Mashayekhi A, Arshi S, Majidpour A, Babaei M, Mosavi A, Mohagheghi MMA, Alimohammadian M	Int J Cancer	107	2003
Declining incidence of esophageal cancer in the Turkmen Plain, eastern part of the Caspian Littoral of Iran: A retrospective cancer surveillance	01/1996 to 12/2000	Subnational	Iran (Islamic Republic of), GolestÄn	Scientific literature				Semnani S, Sadjadi A, Fahimi S, Nouraie M, Naeimi M, Kabir J, Fakheri H, Saadatnia H, Ghavamnasiri MR, Malekzadeh R	Cancer Detect Prev	30	2006
Iran - Ardabil Cancer Registry Extracts 1985-2008	01/1985 to 12/2008	Subnational	Iran (Islamic Republic of), ArdabÄ«l	Disease registry			Digestive Diseases Research Center (Iran), Ardabil University of Medical Sciences, International Agency for Research on Cancer (IARC)				
Iran - Golestan Cancer Registry Incidence Data 2006-2008	01/2006 to 12/2008	Subnational	Iran (Islamic Republic of), GolestÄn	Disease registry			Center for Disease Control and Prevention, Ministry of Health and Medical Education (Iran), Digestive Diseases Research Center (Iran), Golestan University of Medical Sciences (Iran)				
Iran National Cancer Registry 2000	01/2000 to 12/2000	Country	Iran (Islamic Republic of)	Disease registry	Subnationally representative	Iran National Cancer Registry	Center for Disease Control and Prevention, Ministry of Health and Medical Education (Iran)				

Supplementary Table 1

Title	Time period covered	Coverage type	Geography	Data type	Secondary data type	Series or system	Contributors	Authors	Journal	Volume	Publication year
Iran National Cancer Registry 2001	01/2001 to 12/2001	Country	Iran (Islamic Republic of)	Disease registry	Subnationally representative	Iran National Cancer Registry	Center for Disease Control and Prevention, Ministry of Health and Medical Education (Iran)				
Iran National Cancer Registry 2002	01/2002 to 12/2002	Country	Iran (Islamic Republic of)	Disease registry	Subnationally representative	Iran National Cancer Registry	Center for Disease Control and Prevention, Ministry of Health and Medical Education (Iran)				
Iran National Cancer Registry 2003	01/2003 to 12/2003	Country	Iran (Islamic Republic of)	Disease registry	Subnationally representative	Iran National Cancer Registry	Center for Disease Control and Prevention, Ministry of Health and Medical Education (Iran)				
Iran National Cancer Registry 2004	01/2004 to 12/2004	Country	Iran (Islamic Republic of)	Disease registry	Subnationally representative	Iran National Cancer Registry	Center for Disease Control and Prevention, Ministry of Health and Medical Education (Iran)				
Iran National Cancer Registry 2005	01/2005 to 12/2005	Country	Iran (Islamic Republic of)	Disease registry	Subnationally representative	Iran National Cancer Registry	Center for Disease Control and Prevention, Ministry of Health and Medical Education (Iran)				
Iran National Cancer Registry 2006	01/2006 to 12/2006	Country	Iran (Islamic Republic of)	Disease registry	Subnationally representative	Iran National Cancer Registry	Center for Disease Control and Prevention, Ministry of Health and Medical Education (Iran)				
Iran National Cancer Registry 2007	01/2007 to 12/2007	Country	Iran (Islamic Republic of)	Disease registry	Subnationally representative	Iran National Cancer Registry	Center for Disease Control and Prevention, Ministry of Health and Medical Education (Iran)				
Iran National Cancer Registry 2008	01/2008 to 12/2008	Country	Iran (Islamic Republic of)	Disease registry	Subnationally representative	Iran National Cancer Registry	Center for Disease Control and Prevention, Ministry of Health and Medical Education (Iran)				
Iran National Cancer Registry 2009	01/2009 to 12/2009	Country	Iran (Islamic Republic of)	Disease registry	Subnationally representative	Iran National Cancer Registry	Center for Disease Control and Prevention, Ministry of Health and Medical Education (Iran)				
Iran National Cancer Registry 2010	01/2010 to 12/2010	Country	Iran (Islamic Republic of)	Disease registry	Subnationally representative	Iran National Cancer Registry	Center for Disease Control and Prevention, Ministry of Health and Medical Education (Iran)				
Iran National Cancer Registry Report 2003-2004	03/2003 to 02/2004	Country	Iran (Islamic Republic of)	Disease registry		Iran National Cancer Registry	Center for Disease Control and Prevention, Ministry of Health and Medical Education (Iran)				

Supplementary Table 1

Title	Time period covered	Coverage type	Geography	Data type	Secondary data type	Series or system	Contributors	Authors	Journal	Volume	Publication year
Iran National Cancer Registry Report 2004-2005	03/2004 to 02/2005	Country	Iran (Islamic Republic of)	Disease registry		Iran National Cancer Registry	Ministry of Health and Medical Education (Iran)				
Iran National Cancer Registry Report 2005-2006	03/2005 to 02/2006	Country	Iran (Islamic Republic of)	Disease registry		Iran National Cancer Registry	Center for Disease Control and Prevention, Ministry of Health and Medical Education (Iran)				
Iran National Cancer Registry Report 2006-2007	03/2006 to 02/2007	Country	Iran (Islamic Republic of)	Disease registry		Iran National Cancer Registry	Center for Disease Control and Prevention, Ministry of Health and Medical Education (Iran)				
Iran National Cancer Registry Report 2008-2009	03/2008 to 02/2009	Country	Iran (Islamic Republic of)	Disease registry		Iran National Cancer Registry	Center for Disease Control and Prevention, Ministry of Health and Medical Education (Iran)				
Iran National Cancer Registry Report 2009-2010	03/2009 to 03/2010	Country	Iran (Islamic Republic of)	Disease registry		Iran National Cancer Registry	Center for Disease Control and Prevention, Ministry of Health and Medical Education (Iran)				
