# Supplementary material for: National and subnational burden of brain and central nervous system cancers in Iran, 1990–2019: Results from the global burden of disease study 2019
Source: Cancer Med. 2023 Jan 9;12(7):8614–28. doi: 10.1002/cam4.5553 (PMC10134290; doi:10.1002/cam4.5553)
Supplement: Supplementary file 8 — Table S2. [file CAM4-12-8614-s008.docx]

| **Region** | **1990** | | | | |  | |  | **2019** | | |  | | |
| --- | --- | --- | --- | --- | --- | --- | --- | --- | --- | --- | --- | --- | --- | --- |
|  | **YLL** | | | **YLD** | | | |  | **YLL** | | | **YLD** | | |
|  | **T** | **F** | **M** | **T** | **F** | | **M** |  | **T** | **F** | **M** | **T** | **F** | **M** |
| **Iran (Islamic Republic of)** | 173.3 (101.1–221.7) | 149.1 (69.7–244.3) | 195.6 (102.8–272.4) | 1.8 (1.0–2.6) | 1.5 (0.7–2.7) | | 2.1 (1.0–3.3) |  | 153.5 (80.7–183.6) | 144.4 (74.7–181.2) | 162.5 (71.8–208.2) | 2.9 (1.3–4.3) | 2.6 (1.2–3.9) | 3.2 (1.2–4.9) |
| **Alborz** | 163.6 (87.8–232.7) | 139.9 (65.9–265.2) | 185.2 (88.1–300.5) | 1.9 (0.9–2.8) | 1.5 (0.7–2.9) | | 2.1 (0.9–3.8) |  | 152.5 (75.6–196) | 139.2 (66.4–183.5) | 165.6 (62.9–232.8) | 3.7 (1.6–5.6) | 3.1 (1.4–4.8) | 4.2 (1.4–6.7) |
| **Ardebil** | 149.0 (99.0–225.4) | 122.4 (63.5–244.8) | 173 (97.2–281.9) | 1.4 (0.8–2.1) | 1.1 (0.5–2.3) | | 1.7 (0.9–2.8) |  | 143.4 (85.3–178.1) | 123.7 (76.6–156.1) | 163.6 (78.6–220.8) | 2.5 (1.3–3.7) | 2.1 (1.1–3.2) | 3.0 (1.2–4.7) |
| **Bushehr** | 162.2 (86.3–219) | 144.4 (61.4–242.4) | 179.1 (85.4–264.9) | 1.7 (0.8–2.5) | 1.4 (0.6–2.5) | | 1.9 (0.8–3) |  | 144.2 (76.1–182.5) | 140.9 (73.7–189.8) | 148.0 (66.2–204.5) | 2.8 (1.3–4.2) | 2.6 (1.2–4.0) | 3.0 (1.2–4.9) |
| **Chahar Mahaal and Bakhtiari** | 166.2 (93.4–231.3) | 130.4 (54.2–227.7) | 198.4 (94.5–313.1) | 1.8 (0.9–2.7) | 1.4 (0.6–2.5) | | 2.2 (0.9–3.6) |  | 127.9 (67.6–166.4) | 109.8 (58.1–160.2) | 146.4 (63.4–214.7) | 2.8 (1.3–4.3) | 2.3 (1.1–3.9) | 3.4 (1.3–5.6) |
| **East Azarbayejan** | 231.5 (106.5–326.1) | 199 (69–338.3) | 260.7 (102.8–406.1) | 2.2 (1.0–3.4) | 1.8 (0.6–3.3) | | 2.5 (0.9–4.4) |  | 179.9 (89.9–237.4) | 167.2 (76.5–231.3) | 192.5 (84.7–276.7) | 3.3 (1.4–5.0) | 3.1 (1.2–5.2) | 3.5 (1.2–5.9) |
| **Fars** | 176.2 (96.1–237.5) | 154 (64.7–250.8) | 196.9 (94.7–290.7) | 1.8 (1.0–2.7) | 1.6 (0.6–2.7) | | 2.1 (1.0–3.5) |  | 179.9 (89.9–237.4) | 167.2 (76.5–231.3) | 192.5 (84.7–276.7) | 3.7 (1.6–5.7) | 3.2 (1.3–5.3) | 4.1 (1.5–6.7) |
| **Gilan** | 114.6 (84.4–158.7) | 94.7 (56.4–149.4) | 134.5 (90.2–205) | 1.2 (0.8–1.8) | 1.0 (0.5–1.7) | | 1.5 (0.9–2.4) |  | 119 (79.9–143.1) | 107.1 (71.3–139.2) | 131.0 (71.4–173.5) | 2.4 (1.4–3.4) | 2.0 (1.1–3.1) | 2.8 (1.3–4.1) |
| **Golestan** | 146.2 (92.5–208.1) | 122.5 (67.1–204.7) | 168.5 (90.3–273.6) | 1.3 (0.8–2.0) | 1.1 (0.6–1.9) | | 1.6 (0.8–2.6) |  | 140.6 (96.1–169.2) | 124.6 (83.9–156.5) | 157 (87.5–204.3) | 2.2 (1.3–3.2) | 1.9 (1.1–2.8) | 2.5 (1.2–3.9) |
| **Hamadan** | 157.1 (102–224) | 133.8 (64–231.4) | 178.8 (96.7–289.4) | 1.5 (0.9–2.3) | 1.3 (0.6–2.3) | | 1.8 (0.9–3) |  | 139.6 (88.1–172.3) | 126.5 (77.5–163.8) | 152.8 (84.3–207.1) | 2.5 (1.3–3.7) | 2.2 (1.1–3.4) | 2.8 (1.3–4.3) |
| **Hormozgan** | 119.4 (65.5–191.2) | 105.4 (49.2–195.6) | 131.6 (69.5–241) | 1.1 (0.6–1.7) | 0.9 (0.4–1.7) | | 1.2 (0.6–2.2) |  | 113.7 (82.8–138.9) | 109.4 (70.4–144.4) | 117.8 (77.6–157.4) | 1.8 (1.2–2.6) | 1.7 (1.0–2.6) | 2.0 (1.1–3.0) |
| **Ilam** | 145 (88.9–195.4) | 120 (53.4–209.2) | 165.3 (88.2–245.8) | 1.5 (0.8–2.2) | 1.2 (0.5–2.0) | | 1.7 (0.9–2.8) |  | 138.8 (81.6–172.2) | 127.3 (75.3–166.7) | 149.9 (73.9–201.1) | 2.7 (1.4–4) | 2.3 (1.2–3.5) | 3.1 (1.4–4.9) |
| **Isfahan** | 165.2 (84.3–225.7) | 144 (64.9–224.7) | 185.3 (84.2–286.3) | 1.9 (0.9–2.8) | 1.6 (0.7–2.7) | | 2.2 (0.9–3.6) |  | 166.3 (73.4–218.4) | 159.2 (67.8–218.8) | 173.2 (61.5–250.5) | 3.5 (1.3–5.4) | 3.1 (1.3–5.0) | 3.8 (1.2–6.3) |
| **Kerman** | 190.5 (101–259.2) | 164.5 (66.4–276.1) | 214.2 (99.2–329.7) | 1.8 (0.9–2.7) | 1.5 (0.6–2.6) | | 2.1 (0.9–3.4) |  | 164.1 (87.9–210) | 159.1 (75.7–213.8) | 169.1 (80.6–238.1) | 2.6 (1.2–4.0) | 2.5 (1.1–3.9) | 2.8 (1.1–4.4) |
| **Kermanshah** | 230.7 (102.3–328.5) | 185.9 (66.1–318.4) | 269.4 (99.9–430.4) | 2.1 (0.9–3.3) | 1.7 (0.6–3.1) | | 2.5 (0.9–4.3) |  | 184.7 (92.7–246.4) | 164.8 (77.5–231.5) | 204.9 (85.4–305.4) | 3.1 (1.3–4.8) | 2.6 (1.1–4.4) | 3.5 (1.3–5.9) |
| **Khorasan–e–Razavi** | 195.9 (105.7–270.6) | 163.2 (65.9–293.6) | 226.1 (100.7–353.3) | 1.8 (0.9–2.7) | 1.4 (0.6–2.6) | | 2.1 (0.9–3.7) |  | 164.6 (88.9–210.7) | 151.6 (78.7–209.6) | 178.1 (76.4–250.6) | 2.8 (1.4–4.2) | 2.5 (1.2–3.9) | 3.1 (1.2–5.1) |
| **Khuzestan** | 131.6 (96.8–178.2) | 111.0 (67.9–175) | 150.7 (96.4–226.6) | 1.3 (0.8–1.9) | 1.1 (0.6–1.8) | | 1.5 (0.9–2.5) |  | 147.7 (87.5–183.2) | 137.2 (80.5–178.8) | 158.3 (77–214) | 2.6 (1.4–3.9) | 2.3 (1.2–3.6) | 2.9 (1.2–4.6) |
| **Kohgiluyeh and Boyer–Ahmad** | 149.4 (92.5–210.6) | 117.7 (54.9–208) | 178.3 (90.8–280.8) | 1.5 (0.8–2.2) | 1.1 (0.5–2.1) | | 1.8 (0.8–3) |  | 150.8 (81.6–197.8) | 130.4 (78.1–178.9) | 170.3 (71.3–247.5) | 3.2 (1.5–4.9) | 2.6 (1.3–4.1) | 3.7 (1.4–6.2) |
| **Kurdistan** | 198.1 (105–286.1) | 171.0 (60.5–339.3) | 221.5 (100.5–354) | 1.9 (1.0–2.9) | 1.5 (0.6–3.2) | | 2.1 (0.9–3.7) |  | 150.7 (81.3–192) | 140.4 (71.8–190) | 160.9 (75.9–225.3) | 2.5 (1.2–3.7) | 2.2 (1.1–3.5) | 2.8 (1.2–4.5) |
| **Lorestan** | 168.9 (93.9–238.3) | 138.2 (61.5–252.1) | 195.6 (94.2–311.4) | 1.7 (0.9–2.5) | 1.3 (0.6–2.5) | | 2.0 (0.9–3.4) |  | 153.4 (77.5–205.4) | 136.1 (63.5–192.9) | 171.6 (73.8–254.5) | 2.8 (1.3–4.4) | 2.4 (1.1–3.9) | 3.3 (1.2–5.5) |
| **Markazi** | 236.9 (104.2–335.1) | 201.5 (69.6–351.5) | 270.9 (101.2–426.9) | 2.3 (1.0–3.7) | 1.9 (0.6–3.4) | | 2.6 (0.9–4.6) |  | 188.8 (77.5–253.7) | 169.4 (70.1–234.6) | 208.1 (69.1–313.1) | 3.4 (1.2–5.4) | 2.9 (1.1–4.8) | 3.9 (1.1–6.7) |
| **Mazandaran** | 130.1 (91.1–168.3) | 112.8 (64.8–180) | 147 (91.4–210.4) | 1.5 (0.9–2.2) | 1.3 (0.7–2.2) | | 1.8 (1.0–2.8) |  | 146.9 (77.4–189.3) | 138 (70.8–184.3) | 155.9 (68–220.1) | 3.4 (1.6–5) | 2.9 (1.4–4.7) | 3.8 (1.4–6) |
| **North Khorasan** | 155.8 (94.3–234.6) | 136.1 (61.5–267.5) | 173.7 (94.9–302.8) | 1.4 (0.8–2.2) | 1.2 (0.5–2.3) | | 1.6 (0.8–2.8) |  | 150 (90.3–184.2) | 149.4 (81.6–195.2) | 151.1 (82.7–199.1) | 2.3 (1.2–3.4) | 2.2 (1.1–3.5) | 2.4 (1.1–3.7) |
| **Qazvin** | 184.3 (94.7–261.3) | 169.9 (60.9–328.7) | 197.4 (90.5–305.2) | 1.8 (0.8–2.7) | 1.6 (0.6–3.2) | | 1.9 (0.8–3.3) |  | 185.6 (72.4–250.2) | 173.1 (64.3–242.4) | 198.5 (65.7–293) | 3.3 (1.2–5.2) | 2.9 (1.0–5) | 3.7 (1.1–6.1) |
| **Qom** | 217.6 (90.9–301.8) | 202.4 (66.7–353) | 231.2 (89.1–367.1) | 2.0 (0.8–3.2) | 1.8 (0.6–3.4) | | 2.2 (0.8–3.9) |  | 186.4 (68.4   \| – \| \| --- \|   251.2) | 185.2 (65.3   \| – \| \| --- \|   260.0) | 188.0 (55.7   \| – \| \| --- \|   282.4) | 3.2 (1.1–5.2) | 3.0 (1.0–5.0) | 3.4 (1.0–5.8) |
| **Semnan** | 162.3 (94.1–218.5) | 137.5 (61.7–229.7) | 186.5 (92.1–283.1) | 1.6 (0.9–2.4) | 1.4 (0.6–2.5) | | 1.9 (0.9–3.1) |  | 136.9 (72.1–172.4) | 128.9 (67.6–171.4) | 145.1 (66.9–196.9) | 2.7 (1.3–3.9) | 2.4 (1.1–3.7) | 3 (1.2–4.7) |
| **Sistan and Baluchistan** | 104.1 (47.2–219.1) | 75.5 (31.3–161.7) | 129.3 (53.9–296.8) | 0.9 (0.4–1.7) | 0.6 (0.3–1.3) | | 1.1 (0.5–2.5) |  | 107.6 (88–143.4) | 92.7 (69.8–133.3) | 122.5 (88.2–172.2) | 1.3 (0.9–1.9) | 1.1 (0.7–1.7) | 1.5 (0.9–2.4) |
| **South Khorasan** | 160.2 (96.7–230.8) | 130.4 (61.5–242.2) | 187.7 (95.5–308.5) | 1.5 (0.9–2.3) | 1.2 (0.6–2.3) | | 1.8 (0.9–3.1) |  | 145.5 (78.9–183.4) | 137.9 (74.3–184.9) | 153.7 (69.1–211) | 2.4 (1.2–3.6) | 2.2 (1–3.5) | 2.7 (1.1–4.1) |
| **Tehran** | 175.8 (80.7–260.6) | 159.6 (79.1–304) | 190.9 (68.3–323.1) | 2.2 (0.9–3.6) | 2.0 (0.8–4.1) | | 2.5 (0.8–4.5) |  | 134.5 (54.4–171.8) | 133.3 (59–173.7) | 135.8 (36.3–193) | 2.9 (1.1–4.4) | 2.7 (1.1–4.1) | 3.2 (0.8–5.2) |
| **West Azarbayejan** | 212.5 (101.7–292) | 184.8 (68–310.8) | 237.7 (100–362.7) | 1.9 (0.9–2.9) | 1.6 (0.6–2.8) | | 2.2 (0.9–3.7) |  | 180.3 (83.8–234.1) | 170.4 (78.9–236) | 190.6 (76.6–271.7) | 3.0 (1.3–4.7) | 2.7 (1.1–4.5) | 3.3 (1.1–5.4) |
| **Yazd** | 233.7 (91.8–334.2) | 216.7 (70.1–369.4) | 251.7 (87.8–397.3) | 2.3 (0.8–3.6) | 2.1 (0.6–3.8) | | 2.5 (0.7–4.5) |  | 213.6 (75.6–296.4) | 210.8 (73.5–304.1) | 215.9 (63.6–338.8) | 4.5 (1.4–7.5) | 4.2 (1.3–7.0) | 4.9 (1.2–8.8) |
| **Zanjan** | 156.2 (100.6–202.6) | 130.6 (65.8–225.9) | 180.2 (98.7–261.1) | 1.5 (0.9–2.2) | 1.2 (0.6–2.2) | | 1.8 (0.9–2.9) |  | 124.1 (71.0–151.4) | 111.7 (63.3–146.4) | 137.2 (62.8–183.3) | 2.2 (1.1–3.3) | 1.9 (1.0–3.0) | 2.5 (1.1–3.9) |

Data in parentheses are 95% Uncertainty Intervals (95% UIs); DALYs= Disability-Adjusted Life Years; YLLs= Years of Life Lost; YLDs= Years Lived with Disability
