# Supplementary material for: National and subnational burden of brain and central nervous system cancers in Iran, 1990–2019: Results from the global burden of disease study 2019
Source: Cancer Med. 2023 Jan 9;12(7):8614–28. doi: 10.1002/cam4.5553 (PMC10134290; doi:10.1002/cam4.5553)
Supplement: Supplementary file 9 — Table S3. [file CAM4-12-8614-s004.docx]

| Location | | New cases | | | | | | Expected new cases in 2019 | | | | | |
| --- | --- | --- | --- | --- | --- | --- | --- | --- | --- | --- | --- | --- | --- |
|  |  | 1990 | | | 2019 | | | Population growth | | | Population growth + Aging | | |
|  |  | T | F | M | T | F | M | T | F | M | T | F | M |
| Iran (Islamic Republic of) | | 2523 | 1050 | 1472 | 5811 | 2653 | 3157 | 3632 | 1522 | 2107 | 4458 | 1869 | 2569 |
| Subnational | Alborz | 62 | 24 | 38 | 238 | 103 | 135 | 121 | 49 | 73 | 158 | 66 | 91 |
|  | Ardebil | 42 | 16 | 26 | 81 | 34 | 47 | 47 | 18 | 28 | 55 | 22 | 32 |
|  | Bushehr | 26 | 11 | 15 | 75 | 35 | 40 | 45 | 19 | 26 | 57 | 24 | 33 |
|  | Chahar Mahaal and Bakhtiari | 30 | 11 | 19 | 63 | 26 | 36 | 41 | 16 | 25 | 52 | 20 | 31 |
|  | East Azarbayejan | 197 | 82 | 115 | 339 | 159 | 180 | 233 | 97 | 136 | 288 | 119 | 166 |
|  | Fars | 163 | 70 | 93 | 432 | 193 | 240 | 221 | 95 | 126 | 277 | 116 | 160 |
|  | Gilan | 71 | 29 | 42 | 172 | 75 | 96 | 79 | 32 | 47 | 112 | 45 | 66 |
|  | Golestan | 44 | 18 | 26 | 105 | 46 | 58 | 64 | 26 | 38 | 78 | 33 | 45 |
|  | Hamadan | 66 | 27 | 39 | 110 | 49 | 61 | 68 | 29 | 39 | 83 | 34 | 48 |
|  | Hormozgan | 24 | 10 | 14 | 77 | 36 | 41 | 49 | 21 | 29 | 55 | 24 | 31 |
|  | Ilam | 15 | 6 | 9 | 37 | 16 | 21 | 20 | 8 | 12 | 25 | 10 | 15 |
|  | Isfahan | 171 | 72 | 99 | 446 | 205 | 241 | 234 | 101 | 103 | 298 | 125 | 172 |
|  | Kerman | 82 | 35 | 48 | 211 | 99 | 112 | 147 | 61 | 86 | 177 | 73 | 102 |
|  | Kermanshah | 89 | 34 | 55 | 158 | 69 | 89 | 104 | 41 | 63 | 140 | 55 | 83 |
|  | Khorasan-e-Razavi | 228 | 93 | 135 | 450 | 204 | 245 | 322 | 131 | 191 | 365 | 148 | 215 |
|  | Khuzestan | 97 | 40 | 57 | 292 | 132 | 160 | 148 | 61 | 87 | 182 | 75 | 106 |
|  | Kohgiluyeh and Boyer-Ahmad | 18 | 7 | 11 | 50 | 21 | 30 | 27 | 10 | 17 | 30 | 11 | 18 |
|  | Kurdistan | 60 | 25 | 35 | 104 | 47 | 57 | 80 | 33 | 47 | 93 | 39 | 54 |
|  | Lorestan | 59 | 23 | 36 | 123 | 54 | 69 | 67 | 26 | 41 | 90 | 37 | 52 |
|  | Markazi | 72 | 30 | 43 | 129 | 55 | 73 | 86 | 35 | 51 | 109 | 44 | 64 |
|  | Mazandaran | 92 | 39 | 53 | 284 | 129 | 155 | 120 | 51 | 70 | 166 | 71 | 94 |
|  | North Khorasan | 23 | 10 | 13 | 50 | 25 | 26 | 31 | 13 | 18 | 37 | 16 | 21 |
|  | Qazvin | 41 | 18 | 23 | 109 | 50 | 59 | 57 | 25 | 31 | 72 | 32 | 29 |
|  | Qom | 36 | 16 | 20 | 105 | 50 | 56 | 66 | 30 | 36 | 84 | 38 | 46 |
|  | Semnan | 20 | 8 | 12 | 49 | 22 | 26 | 31 | 13 | 18 | 38 | 16 | 22 |
|  | Sistan and Baluchistan | 34 | 11 | 22 | 87 | 37 | 50 | 68 | 23 | 45 | 69 | 24 | 44 |
|  | South Khorasan | 28 | 11 | 17 | 50 | 23 | 27 | 35 | 13 | 21 | 38 | 15 | 22 |
|  | Tehran | 442 | 192 | 250 | 956 | 459 | 479 | 724 | 322 | 401 | 882 | 394 | 485 |
|  | West Azarbayejan | 119 | 51 | 68 | 250 | 116 | 134 | 174 | 75 | 99 | 203 | 85 | 116 |
|  | Yazd | 40 | 18 | 22 | 120 | 57 | 63 | 67 | 30 | 36 | 82 | 36 | 46 |
|  | Zanjan | 33 | 14 | 20 | 60 | 26 | 33 | 41 | 17 | 24 | 50 | 20 | 29 |
